# Individualized prediction of psychiatric readmissions for patients with major depressive disorder: a 10-year retrospective cohort study

**DOI:** 10.1038/s41398-022-01937-7

**Published:** 2022-04-23

**Authors:** Ting Zhu, Jingwen Jiang, Yao Hu, Wei Zhang

**Affiliations:** 1 West China Biomedical Big Data Center, West China Hospital, Sichuan University, Chengdu, China; 2 Med-X Center for Informatics, Sichuan University, Chengdu, China; 3 Mental Health Center of West China Hospital, Sichuan University, Chengdu, China

**Keywords:** Depression, Scientific community

## Abstract

Patients with major depressive disorder (MDD) are at high risk of psychiatric readmission while the factors associated with such adverse illness trajectories and the impact of the same factor at different follow-up times remain unclear. Based on machine learning (ML) approaches and real-world electronic medical records (EMR), we aimed to predict individual psychiatric readmission within 30, 60, 90, 180, and 365 days of an initial major depression hospitalization. In addition, we examined to what extent our prediction model could be made interpretable by quantifying and visualizing the features that drive the predictions at different follow-up times. By identifying 13,177 individuals discharged from a hospital located in western China between 2009 and 2018 with a recorded diagnosis of MDD, we established five prediction-modeling cohorts with different follow-up times. Four different ML models were trained with features extracted from the EMR, and explainable methods (SHAP and Break Down) were utilized to analyze the contribution of each of the features at both population-level and individual-level. The model showed a performance on the holdout testing dataset that decreased over follow-up time after discharge: AUC 0.814 (0.758–0.87) within 30 days, AUC 0.780 (0.728–0.833) within 60 days, AUC 0.798 (0.75–0.846) within 90 days, AUC 0.740 (0.687–0.794) within 180 days, and AUC 0.711 (0.676–0.747) within 365 days. Results add evidence that markers of depression severity and symptoms (recurrence of the symptoms, combination of key symptoms, the number of core symptoms and physical symptoms), along with age, gender, type of payment, length of stay, comorbidity, treatment patterns such as the use of anxiolytics, antipsychotics, antidepressants (especially Fluoxetine, Clonazepam, Olanzapine, and Alprazolam), physiotherapy, and psychotherapy, and vital signs like pulse and SBP, may improve prediction of psychiatric readmission. Some features can drive the prediction towards readmission at one follow-up time and towards non-readmission at another. Using such a model for decision support gives the clinician dynamic information of the patient’s risk of psychiatric readmission and the specific features pulling towards readmission. This finding points to the potential of establishing personalized interventions that change with follow-up time.

## Introduction

Major depressive disorder (MDD) is becoming the leading cause of mental health related disease burden globally, affecting an estimated 300 million people worldwide [1]. MDD is a chronic, relapsing, and burdensome disease that can lead to hospitalization [2]. Among MDD patients, rehospitalization is a substantial concern, and the post-hospitalization period is recognized as a high-risk period for outcomes such as suicide and relapse. The absolute risk of suicide after first hospital contact is 3.8% among females and 6.7% among males [3]. Relapse rates of MDD in specialized mental healthcare settings are high (60% after 5 years, 67% after 10, and 85% after 15) [4]. 11 to 30% of patients with MDD reach readmission with initial treatment, even after 12 months [5, 6]. Psychiatric readmission rates have been proposed as a negative quality of care indicator for inpatient psychiatric services. Early hospital readmission has been identified as a preventable driver of healthcare costs and a key quality metric for Medicare [7]. Although the fact that rehospitalization is a preventable outcome, this phenomenon is limited understood because a challenge in reducing readmission risk is the difficulty in identifying individuals at greatest risk, who might benefit from personalized interventions [8]. Evidence from clinical trials and research suggests that increasing the likelihood of intervention by a community-based institution is valuable to reduce readmission risk [9]. Considering psychiatric rehospitalization may be a major reflection of disease relapse or acute exacerbation, individualized early detection of psychiatric rehospitalization risk is critical for the mental and physical well-beings of patients with high risk. Research studies that demonstrated how effective and individualized prediction of psychiatric readmission risk of MDD patients could be realized are needed, to help timely inform intervention strategies to prevent readmissions and further reduce healthcare costs.

Various efforts have been made to predict psychiatric rehospitalization within 7 days [10], 30 days [11, 12], 90 days [13], 180 days [14] or 2 years [4] post-discharge. Interestingly, the predictive risk factors of readmission over a longer period appear to differ from those over the shorter period [15, 16]. Although a few high-performance models were reported in studies where the cohorts only track for less than 5 years [2, 17–19], prediction accuracy was limited when applied to a follow-up of more than 5 years. Many risk factors of readmission (such as a greater impairment in self-care, more severe symptoms, more persistent illnesses, treatment patterns, bad adherence to anti-depression medications, biomarkers of neuroimaging, blood, and sleep, etc.) have been identified [4, 20]. Such findings have been beneficial in constructing clinical evidence of readmission. However, few studies in psychiatry have attempted to develop clinically actionable and explainable prediction rules [21, 22] based on validated prediction outcomes and large size of sample [4], and little research has examined the contribution of the same risk factor to readmission prediction models with different (short and long) follow-up times. To facilitate this, a longitudinal, multivariate sample of clinically diagnosed inpatients is required. Electronic medical records (EMR) provide high fidelity heterogeneous data of clinically diagnosed inpatients’ information, yet the utilization of this data to predict MDD readmission risk remains insufficient. Modelling techniques that prognosticate individually, rather than at the group level are needed given the heterogeneous nature of MDD and its illness trajectories [4].

While univariate and multivariate analyses [23, 24] such as binary logistic regression [25, 26] or survival analysis using cox proportional hazards regression [27–29] have been the dominant approaches in revealing the different roles of individual-level and population-level factors, reasons for readmission could be associated to a complex network. The above methods do not guarantee predictive optimality and do not guarantee parsimony in a data analysis-independent manner [30]. Zheng et al. [31] highlighted the implementation of advanced machine learning (ML) technology and the utilization of a high-dimensional dataset containing comprehensive clinical profile of patients as two future directions. Compared with traditional statistical modeling methods, ML methods are more flexible to model a variety of clinical qualitative and quantitative data through computer learning [32]. Recent work has shown that ML models are well suited to MDD relapse prediction with a multimodal panel containing structure imaging, blood-biomarker, clinical, medication type and sleep quality predictors [4].

We found three limits in the previous studies. First, the cohort of the study population has a small number of patients, while we establish a 10-year retrospective cohort including about 13,000 patients. Second, most studies used only one of the structured or the non-structured data (deriving information from narrative notes relies on natural language processing) [7]. In our study, we comprehensively incorporated all types of risk factors in real-world EMR data and then validated the utility of EMR data for MDD psychiatric readmission prediction. Third, a recent study employing a combination of clinical and biomarker predictors from different modalities has shown moderate success (AUC of 0.6774) for ML-based MDD readmission prediction [4]. Notably, no studies have applied explainable ML models to readmission prediction, and the individual-level risk factors of psychiatric readmission for the same patient at different follow-up times remain poorly understood.

**Fig. 1:**
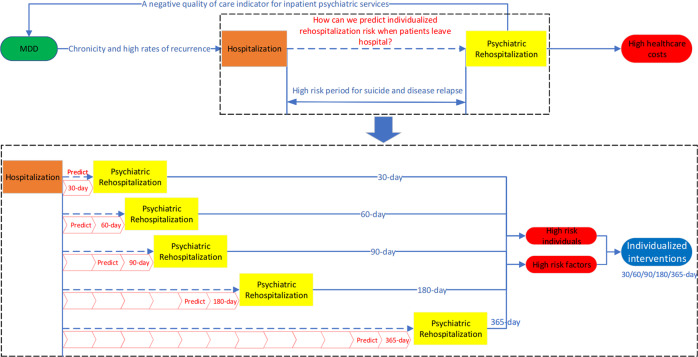
Study overview.

Therefore, using a large, longitudinal EMR routinely captured by the study hospital, this research aims to explore the feasibility of predicting psychiatric readmission within 30, 60, 90, 180, and 365 days after the initial major depression hospitalization by identifying critical risk factors that lead to readmission (Fig. 1), in which both the traditional univariate analyses and explainable ML methods are employed to combine multiple sources of patients’ information.

## Subjects and methods

### Data source

The sample was drawn from the EMR system of West China Hospital (WCH), Sichuan University. WCH is one of the largest single-site hospitals in the world and a leading medical center of West China, treating complicated and severe cases. The Health Information System (HIS) was gradually adopted in WCH in late 2008, through which physicians kept reports of admissions, progresses, and discharges, entered orders, requested, and viewed results of laboratory tests and examinations. Nurses and faculties from clinical laboratories also managed patients and controlled the quality of clinical practice via the HIS and the Laboratory Information System (LIS), respectively. In 2009, an EMR system integrated with the HIS and the LIS was adopted in all departments throughout the hospital, which was set as the starting time of our data extraction.

### Study subjects

**Fig. 2:**
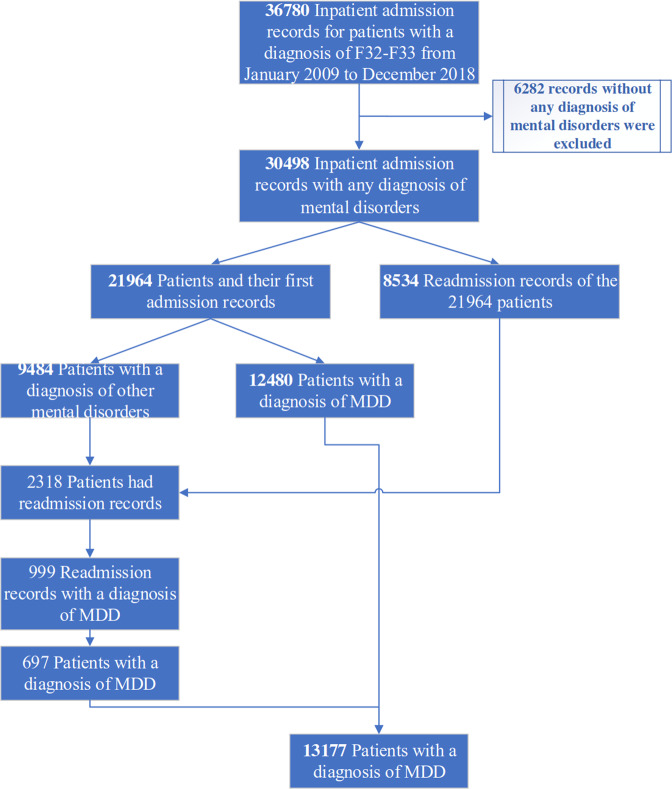
Flow diagram of the subject inclusion/exclusion process.

The aim of this study was to predict the risk of an MDD individual psychiatric readmission within 30, 60, 90, 180, and 365 days based on medical records of the index admission. The index admission was defined as the initial inpatient record of major depression for each patient. The initial sample comes from a research database created by extracting information from the EMR of WCH, containing 36,780 admission records of 21,964 inpatients with a diagnosis name including the words either “depression”, “mania”, or “bipolar disorder”, who were hospitalized and then discharged at least once between January 1, 2009 and December 31, 2018. At baseline, a sample contained 13,177 MDD patients were recruited as the study population and each patient’s index admission record was extracted for analyses (Supplementary Information 1.1). No other exclusion criteria were applied except for data-cleaning considerations. Fig. 2 shows the process of data extraction.

We used the presence of an F32 or F33 ICD-10 code in either the main or supplementary position to signify an MDD admission. To note, the ICD-10 code present in the main diagnostic position indicates the principal condition the patient is being treated for, whilst the supplementary codes signify active comorbidities that have contributed to the overall episode [27]. In such setting, the psychiatric rehospitalization risk of all MDD patients who were hospitalized both for an acute episode of MDD and not for an acute episode of MDD could be predicted and the prediction tool can be utilized in a wider range of scenarios.

### Measures

#### Definition of psychiatric readmission

**Fig. 3:**
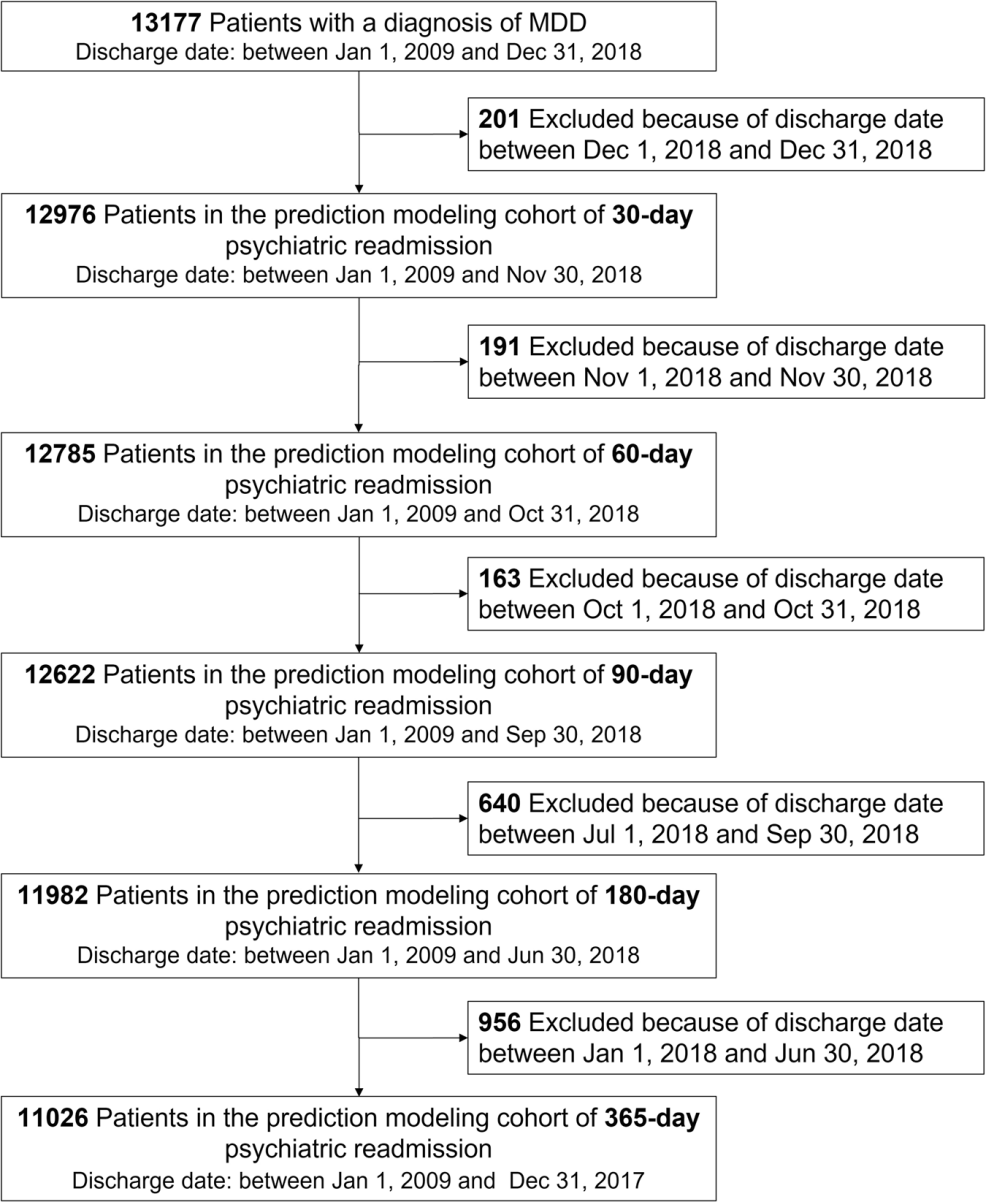
Cohort establishment for the 30-, 60-, 90-, 180-, and 365-day psychiatric readmission prediction.

The psychiatric readmission measure in this study was defined as a binary indicator of whether an individual had another admission record within 30 days (60, 90, 180, and 365 days) after the initial MDD hospitalization, with a principal psychiatric diagnosis ICD-10 code in the range F00-F99. For example, the prediction-modeling cohort of 30-day psychiatric readmission, cases in the cohort referred to patients whose discharge date of the index admission located between January 2009 and November 2018 (Fig. 3). For each patient, follow-up began at the date of discharge, and ended at the earliest instance of 30-day psychiatric readmission (if at all), or the 30-day follow-up end date after discharge. Notably, patients whose discharge date of the index admission located between December 1, 2018 and December 31, 2018 were removed from the initial cohort, since the follow-up cannot cover a whole month (Supplementary Information 1.2).

#### Variables and features

Following the Andersen sociobehavioral model [33, 34] and other previous studies, the determinants of readmission included predisposing factors, including age, sex (male/female), marital status, and race ethnicity; enabling factors, including whether the patient had a diagnosis of a medical comorbidity, source of payment, primary psychiatric diagnosis, severity of symptoms and length of stay of the index admission [10].

In our study, the database consists of a representative set of items to record all information generated during a patient’s hospitalization. Various categories of features were extracted from the original medical records. Specifically, sociodemographic information includes age at admission, gender, marital status, job, ethnicity, source of payment, and province of hometown. Basic information about hospitalization like month/seasonality/year at admission, specialty care unit that the patient lived, whether transferred to other units, diagnoses, severity of MDD, whether the diagnosis of a patient is a recurrent MDD, whether the patient had a diagnosis of a medical comorbidity that increases the relative risks of psychiatric readmission (other mental disorders, endocrine diseases, nervous diseases, digestive diseases, circulatory diseases, respiratory diseases, and cancer), the number of comorbidity in each disease system, the number of surgery, how many admissions before the index admission, length of stay, vital signs based on basic body check at admission (subcutaneous bleeding, sickly look, facial expression, nutrition, cooperation, consciousness, gait, body position, body temperature, pulse, breath, systolic and diastolic blood pressure), and other information that is typically used for billing purposes were collected. In addition, some information about past illness history and life behavior of a patient was also recorded and extracted, including histories of surgery, allergy, blood transfusion, medication use, smoking, alcoholism. By analyzing doctors’ order dataset, features of treatment patterns of each patient including inpatient medication prescriptions (the type of drug and usage frequency of common antidepressant, anxiolytic, antipsychotic, and anti-side-effect treatments), physiotherapy patterns (modified electroconvulsive therapy, biofeedback therapy, transcranial magnetic stimulation, and electroencephalographic biofeedback therapy), and psychotherapy patterns were captured and extracted from the EMR system.

Unstructured data like narrative notes (chief complaint) to record patients’ main symptoms and current mood states was processed by removing all digits (duration of the specific symptom) and punctuation. The text data was split into words by jieba package of R (Version 3.6.1 for Windows). By adding some stop words and medical dictionaries, meaningless words were deleted, and some medical nouns were prevented from being separated. The term frequency scores were used to identify the most informative words in patients’ chief complaints and these words were referred to as features of patients’ main symptoms and current mood states (mood-down, bad sleep, loss of interest, flustered, worry, tension, upset, headache, dizziness, physical discomfort, fatigue, suicide ideation, self-harm, hallucination, less activity, chest tightness, afraid, irritability, fidget, slow response, recurrence and worsen of symptoms). The value of each of the symptom feature depends on whether the chief complaint of this patient includes the above key words or not, particularly, 1 for including the specific word and 0 for not including the specific word (Supplementary Information 1.3).

Overall, 232 features were recruited into the original data pool (Supplementary Information 1.4). The details of recruited features were concluded in Table S1. Drugs, physiotherapies, and psychotherapies used in the antidepressant therapy were summarized in Table S2. Furthermore, we analyzed the quality of our raw data. The results were presented in Table S3 and S4. The data were checked for missing values, and missing values were filled with data in the EMR. The outliers of each record were detected and were removed before the start of the analysis.

### 2.4 ML models

Data analyses and ML algorithms were implemented using RStudio (Version 3.6.1 for Windows). The process of model development and evaluation were accomplished in three phases.

#### Model building

We trained four different ML models such as a multivariate binary logistic regression (LR) model, a Radial Basis Function Kernel two-class support vector machine (SVM) model, a random forest (RF) model and an extreme gradient boosting (XGBoost, XGB) model to predict 30-, 60-, 90-, 180-, and 365-day psychiatric readmissions for patients with MDD, respectively. LR is a statistical approach that presumes a linear relation between log-odds of the predictor variables and the outcome [35]. The expected value of the outcome is fit to the predictors and the regression function is a sigmoid function that can take a real number and transforms it into a value between 0 and 1. LR has been the dominant approach to binary outcome prediction in clinical medicine for decades. SVM generates hyperplanes to separate the data into different regions using a radial kernel function, which enlarges the feature space to accommodate as many data points as possible. RF is an ensemble algorithm that combine numerous independently sampled decision trees via bootstrapping to optimize predictive accuracy. Gradient boosting is a well-known ML technique for classification and regression problems and produces a prediction model in the form of an ensemble of weak prediction models, which are typically decision trees. The XGB algorithm builds the model in a stepwise fashion, like other boosting methods, but is more general than many approaches because it allows the optimization of an arbitrary differentiable loss function. These approaches were selected because they have strong theoretical foundations and vary in complexity.

To assess the prediction performance of ML models on a different dataset and to ensure the unbiased approximation of the model’s generalizability to new patients, each prediction-modeling cohort was split into multiple training and testing datasets according to the period obtained of the data respectively (Supplementary Information 1.5 and Table S5). The initial train-test splits were 1950 patients (15.03% of the 30-day cohort), 1759 patients (13.76% of the 60-day cohort), 1596 patients (12.64% of the 90-day cohort), 956 patients (7.98% of the 180-day cohort), and 2023 patients (18.35% of the 365-day cohort), respectively, for testing of our final models. All performance metrics were derived from the testing datasets.

For each train-test split of each prediction-modeling cohort (30-, 60-, 90-, 180-, and 365-day), we conducted feature selection, hyperparameter optimization, and the fitting of a SVM, LR, RF, and XGB model in the inner cross validation loop. To begin, for each prediction-modeling cohort, two groups (readmission and non-readmission) were compared regarding all included features using only the training dataset. The feature filtering phase has the benefits of both partly minimization of overfitting and variable selection. Two-sided t test for continuous variables and chi-squared test for categorical variables were used to statistically filter each feature’s *p* value, and those with *p* < 0.05 were considered as significant features and were entered in the ML algorithms. We ran the four algorithms using the training set to build better classifiers in the inner cross-validation loop. We conducted hyperparameter optimization and the fitting of each of the four algorithms. Specifically, the SVM model was derived by ten-fold cross-validation of hyperparameters (gamma and cost) using the tune.svm function. The link function of the LR model was logit for the Bernoulli distributed binary classification dependent variable. The parameters for RF model (number of trees and number of variables tried at each split) were selected by grid search using ten-fold cross-validation on the training set. For XGB, the optimal hyperparameters (number of optimization rounds, maximum tree depth, minimum weight in each child node, minimum loss reduction, regularization penalty, subsampling for regularization, etc.) were selected by grid search using the expand.grid function. The hyperparameters that maximized area under the receiver operator characteristic (ROC) curve (AUC) were selected. All steps were completed in an inner cross-validation loop with five repeats of ten-fold cross-validation. See Supplementary Information 1.6 for further details on the selected hyperparameter values for each algorithm.

The learned parameters were then used to construct a model for the entire training set and to make predictions on the testing set that was never used for model selection or parameter tuning. We remedied the imbalanced nature of the dataset, which had more non-readmissions than readmissions, by experimenting with randomly sub-sampling the negative class (non-readmissions) in the training set using the ovun.sample function to produce a balanced ratio between the positive class (readmissions) and negative class. Testing set distributions were never modified to reflect the reality of class imbalance during prediction, and reported performance reflects those raw distributions. Topic features extracted for characterizing patients’ symptoms were derived from the complete dataset including both training and testing subsets. As derived topics do not incorporate any knowledge of future readmission, the inclusion of the testing set does not lead to overfitting or inflated estimates of discrimination.

To avoid favorable train-test splits in the data, this process was repeated four times for each prediction-modeling cohort. For the testing of our final models, we obtained performance estimates from four different test sets. Statistical significance of the AUCs obtained from different test sets for each algorithm was calculated by DeLong’s test. The code is available upon request.

#### Explainable ML predictions

An important step forward for ML in actual medical decision support is the ability to provide simple explanations of predictions from arbitrarily complex models, helping eliminate the typical trade-off between accuracy and interpretability. In our work, we applied model agnostic methods (including SHapley Additive exPlanations (SHAP) [36] and Break Down [37]) to the prediction models to obtain explanations of the features that drive patient-specific predictions to mitigate the issue of black-box predictions [38]. SHAP is a model-agnostic representation of feature importance where the impact of each feature on a particular prediction is represented using Shapley values inspired by cooperative game theory [36]. A Shapley value states, given the current set of feature values, how much a single feature in the context of its interaction with other features contributes to the difference between the actual prediction and the mean prediction [39]. The main goal of Break Down is to decompose model predictions into parts that can be attributed to specific variables [37], and contributions of variables are calculated sequentially and presented in the form of waterfall plots [38]. Shapley value is an average over Break Down contributions for all possible ordering of variables [38]. For comparison, we also analyzed how specific features contribute in different prediction scenarios, i.e., 30-, 60-, 90-, 180-, and 365-day psychiatric readmission.

#### Model evaluation

Discrimination of the models were assessed using AUC, sensitivity, specificity, PPV, and NPV (the positive and negative predicted values). A high sensitivity (also called the true positive rate, the recall, or probability of detection) may be preferable to achieve so that MDD patients at greatest risk of psychiatric readmissions can be effectively identified. However, specificity (also called the true negative rate) measures the percentage of patients who are correctly identified as not readmitted with any mental disorders. The AUC is a measure comparing different classification models, which combines sensitivity and specificity.

## Results

### Statistical analyses of features

To dissect the heterogeneity between readmitted patients and non-readmitted patients in sociodemographic status, neuropsychological symptomatology, comorbidity, vital signs, lifestyle and risk behavior, and treatment patterns, statistical analyses were conducted for each prediction-modeling cohort. Results of the 30-day cohort were shown in Table 1 and S6. We identified 12976 unique patients with the discharge diagnosis of MDD, with 431 (3.32%) of those patients readmitted with a psychiatric diagnosis within 30 days post-discharge. The median (IQR) age was 46 ([30, 61]) years, and 66.5% of patients were female. The median (IQR) of length of stay was 15 ([10, 21]) days. See Supplementary Information 2.1 for details on the results of statistical analyses of features Tables 2 and 3.

**Table 1:** Socio-demographic characteristics of the 30-day cohort.

Variable	Variable name	All	Non-readmission	Readmission	*P* value
		(*n* = 12,976)	(*n* = 12,545)	(*n* = 431)	
Socio-demographic variables					
Gender = Female (%)	gender	8632 (66.5)	8312 (66.3)	320 (74.2)	0.001
Age at admission (median [IQR])	admission_age	46.00 [30.00, 61.00]	46.00 [30.00, 61.00]	43.00 [26.00, 63.50]	0.134
Age group (%)	age_group				<0.001
0–17		1107 (8.5)	1053 (8.4)	54 (12.5)	
18–35		3016 (23.2)	2904 (23.1)	112 (26.0)	
36–60		5373 (41.4)	5237 (41.7)	136 (31.6)	
61-		3480 (26.8)	3351 (26.7)	129 (29.9)	
Job status (%)	job				0.001
Unknown		2389 (18.4)	2330 (18.6)	59 (13.7)	
Student		1971 (15.2)	1881 (15.0)	90 (20.9)	
Farmer		1763 (13.6)	1714 (13.7)	49 (11.4)	
Worker		588 (4.5)	570 (4.5)	18 (4.2)	
Civil servant		1105 (8.5)	1072 (8.5)	33 (7.7)	
Staff		1653 (12.7)	1608 (12.8)	45 (10.4)	
Freelancer		663 (5.1)	641 (5.1)	22 (5.1)	
Retired		1793 (13.8)	1714 (13.7)	79 (18.3)	
Unemployed		1051 (8.1)	1015 (8.1)	36 (8.4)	
Marital status (%)	marital_status				0.001
Unknown		35 (0.3)	35 (0.3)	0 (0.0)	
Unmarried		2957 (22.8)	2829 (22.6)	128 (29.7)	
Married		8900 (68.6)	8637 (68.8)	263 (61.0)	
Divorced		549 (4.2)	535 (4.3)	14 (3.2)	
Widowed		535 (4.1)	509 (4.1)	26 (6.0)	
Nationality (%)	nationality				0.082
Other		297 (2.3)	289 (2.3)	8 (1.9)	
Han		11,961 (92.2)	11,572 (92.2)	389 (90.3)	
Tibetan		718 (5.5)	684 (5.5)	34 (7.9)	
Type of payment (%)	pay_type				0.017
Cash		7927 (61.1)	7692 (61.3)	235 (54.5)	
City medical insurance		4707 (36.3)	4524 (36.1)	183 (42.5)	
Provincial medical insurance		342 (2.6)	329 (2.6)	13 (3.0)	
Source of patient (%)	pat_source				0.193
Chengdu		6800 (52.4)	6556 (52.3)	244 (56.6)	
Other cities in Sichuan		4424 (34.1)	4297 (34.3)	127 (29.5)	
Other provinces		1742 (13.4)	1682 (13.4)	60 (13.9)	
Foreign		10 (0.1)	10 (0.1)	0 (0.0)	
Province of hometown (%)	hometown				0.574
Other		1216 (9.4)	1182 (9.4)	34 (7.9)	
Sichuan		10,575 (81.5)	10,222 (81.5)	353 (81.9)	
Tibet		554 (4.3)	533 (4.2)	21 (4.9)	
Chongqing		429 (3.3)	411 (3.3)	18 (4.2)	
Guizhou		202 (1.6)	197 (1.6)	5 (1.2)

**Table 2:** Combination of key symptoms of the study population.

Variable	All	Non-readmission	Readmission	*P* value
	(*n* = 12,976)	(*n* = 12,545)	(*n* = 431)	
Combination of key symptoms (%)				<0.001
mood-down + worsen of symptoms	565 (4.4)	537 (4.3)	28 (6.5)	
mood-down	651 (5.0)	627 (5.0)	24 (5.6)	
mood-down + bad sleep + worsen of symptoms	642 (4.9)	619 (4.9)	23 (5.3)	
mood-down + bad sleep + relapse	249 (1.9)	229 (1.8)	20 (4.6)	
mood-down + bad sleep	607 (4.7)	589 (4.7)	18 (4.2)	
mood-down + relapse	257 (2.0)	242 (1.9)	15 (3.5)	
worsen of symptoms	462 (3.6)	454 (3.6)	8 (1.9)	
mood-down + bad sleep + flustered	80 (0.6)	73 (0.6)	7 (1.6)	
mood-down + physical discomfort + worsen of symptoms	48 (0.4)	42 (0.3)	6 (1.4)	
mood-down + loss of interest + worsen of symptoms	178 (1.4)	173 (1.4)	5 (1.2)	
mood-down + relapse + worsen of symptoms	108 (0.8)	103 (0.8)	5 (1.2)	
mood-down + loss of interest + relapse	85 (0.7)	80 (0.6)	5 (1.2)	
mood-down + bad sleep + flustered + worsen of symptoms	80 (0.6)	75 (0.6)	5 (1.2)	
mood-down + suicide + worsen of symptoms	71 (0.5)	66 (0.5)	5 (1.2)	
mood-down + hallucination	54 (0.4)	49 (0.4)	5 (1.2)	
mood-down + bad sleep + physical discomfort + relapse	31 (0.2)	26 (0.2)	5 (1.2)	
mood-down + bad sleep + loss of interest	160 (1.2)	156 (1.2)	4 (0.9)	
relapse	90 (0.7)	86 (0.7)	4 (0.9)	
mood-down + physical discomfort	63 (0.5)	59 (0.5)	4 (0.9)	
mood-down + loss of interest	168 (1.3)	165 (1.3)	3 (0.7)	
mood-down + bad sleep + loss of interest + worsen of symptoms	130 (1.0)	127 (1.0)	3 (0.7)	
mood-down + bad sleep + relapse + worsen of symptoms	96 (0.7)	93 (0.7)	3 (0.7)	
mood-down + suicide	83 (0.6)	80 (0.6)	3 (0.7)	
mood-down + bad sleep + upset	83 (0.6)	80 (0.6)	3 (0.7)	
relapse + worsen of symptoms	70 (0.5)	67 (0.5)	3 (0.7)	
mood-down + bad sleep + loss of interest + relapse	67 (0.5)	64 (0.5)	3 (0.7)	
mood-down + bad sleep + flustered + relapse	58 (0.4)	55 (0.4)	3 (0.7)	
mood-down + flustered + worsen of symptoms	45 (0.3)	42 (0.3)	3 (0.7)	
mood-down + flustered + relapse	27 (0.2)	24 (0.2)	3 (0.7)	
mood-down + suicide + relapse	19 (0.1)	16 (0.1)	3 (0.7)	
mood-down + loss of interest + suicide + worsen of symptoms	13 (0.1)	10 (0.1)	3 (0.7)	
mood-down + flustered + fatigue + relapse	5 (0.0)	2 (0.0)	3 (0.7)	
mood-down + bad sleep + upset + worsen of symptoms	66 (0.5)	64 (0.5)	2 (0.5)	
mood-down + bad sleep + physical discomfort	57 (0.4)	55 (0.4)	2 (0.5)	
mood-down + upset	44 (0.3)	42 (0.3)	2 (0.5)	
mood-down + worry + tension + worsen of symptoms	36 (0.3)	34 (0.3)	2 (0.5)	
mood-down + bad sleep + headache + worsen of symptoms	27 (0.2)	25 (0.2)	2 (0.5)	
mood-down + flustered + upset + worsen of symptoms	22 (0.2)	20 (0.2)	2 (0.5)	
mood-down + tension	19 (0.1)	17 (0.1)	2 (0.5)	
mood-down + bad sleep + fatigue + worsen of symptoms	19 (0.1)	17 (0.1)	2 (0.5)	
bad sleep + flustered	17 (0.1)	15 (0.1)	2 (0.5)	
mood-down + dizziness + worsen of symptoms	17 (0.1)	15 (0.1)	2 (0.5)

**Table 3:** Combination of all therapy types of the study population.

Variable	All	Non-readmission	Readmission	*P* value
	(*n* = 12,976)	(*n* = 12,545)	(*n* = 431)	
Combination of all therapy types for each patient (%)				<0.001
ADP + AP + AA + PHY + PSY	946 (7.3)	917 (7.3)	29 (6.7)	
ADP + AP + AA	374 (2.9)	351 (2.8)	23 (5.3)	
ADP + AP + AA + ASE + PHY + PSY	519 (4.0)	498 (4.0)	21 (4.9)	
ADP + AP + AA + PHY	390 (3.0)	370 (2.9)	20 (4.6)	
ADP + AP + AA + ASE + PSY	313 (2.4)	296 (2.4)	17 (3.9)	
ADP + AP + AA + PSY	592 (4.6)	578 (4.6)	14 (3.2)	
ADP + AA	586 (4.5)	573 (4.6)	13 (3.0)	
ADP + AP + AA + ASE + PHY	232 (1.8)	220 (1.8)	12 (2.8)	
ADP + AA + PHY + PSY	418 (3.2)	407 (3.2)	11 (2.6)	
ADP + AP + AA + ASE	262 (2.0)	251 (2.0)	11 (2.6)	
ADP + AP + AA + OT + PHY + PSY	206 (1.6)	198 (1.6)	8 (1.9)	
ADP + AP + AA + CM + PHY + PSY	160 (1.2)	152 (1.2)	8 (1.9)	
ADP + AP + AA + OT + PHY	102 (0.8)	95 (0.8)	7 (1.6)	
ADP + AP + AA + CM + OT + PHY + PSY	71 (0.5)	64 (0.5)	7 (1.6)	
ADP + AP + AA + ASE + CM + PHY + PSY	131 (1.0)	124 (1.0)	7 (1.6)	
ADP + AP + AA + MSB + ASE + PHY + PSY	74 (0.6)	67 (0.5)	7 (1.6)	
ADP + AA + ASE + PHY	65 (0.5)	60 (0.5)	5 (1.2)	
ADP + AP + AA + ASE + HYP + PHY + PSY	41 (0.3)	36 (0.3)	5 (1.2)	
ADP + AA + PSY	251 (1.9)	247 (2.0)	4 (0.9)	
ADP + AA + PHY	148 (1.1)	144 (1.1)	4 (0.9)	
ADP + AP + AA + OT + PSY	115 (0.9)	111 (0.9)	4 (0.9)	
ADP + AP + AA + CM + PHY	72 (0.6)	68 (0.5)	4 (0.9)	
ADP + AP + AA + ASE + OT + PSY	92 (0.7)	88 (0.7)	4 (0.9)	
ADP + AP + AA + ASE + CM + PSY	62 (0.5)	58 (0.5)	4 (0.9)	
ADP + AP + AA + ASE + CM + OT + PHY + PSY	64 (0.5)	60 (0.5)	4 (0.9)	
ADP + AP + AA + MSB + PSY	117 (0.9)	113 (0.9)	4 (0.9)	
ADP + AP + AA + MSB + PHY + PSY	156 (1.2)	152 (1.2)	4 (0.9)	
ADP + AA + OT	60 (0.5)	57 (0.5)	3 (0.7)	
ADP + AA + CM + PHY + PSY	93 (0.7)	90 (0.7)	3 (0.7)	
ADP + AA + HYP + PHY + PSY	27 (0.2)	24 (0.2)	3 (0.7)	
ADP + AP + PSY	103 (0.8)	100 (0.8)	3 (0.7)	
ADP + AP + PHY + PSY	88 (0.7)	85 (0.7)	3 (0.7)	
ADP + AP + ASE + PHY + PSY	21 (0.2)	18 (0.1)	3 (0.7)	
ADP + AP + AA + ASE + OT	102 (0.8)	99 (0.8)	3 (0.7)	
ADP + AP + AA + ASE + OT + PHY + PSY	138 (1.1)	135 (1.1)	3 (0.7)	
ADP + AP + AA + MSB + ASE + OT + PHY + PSY	26 (0.2)	23 (0.2)	3 (0.7)	
ADP + AP + AA + MSB + ASE + CM + OT + PHY + PSY	8 (0.1)	5 (0.0)	3 (0.7)	
AP + AA	40 (0.3)	38 (0.3)	2 (0.5)	
ADP + PSY	61 (0.5)	59 (0.5)	2 (0.5)	
ADP + AA + OT + PHY + PSY	90 (0.7)	88 (0.7)	2 (0.5)	
ADP + AA + CM + OT + PHY + PSY	24 (0.2)	22 (0.2)	2 (0.5)	
ADP + AA + ASE	390 (3.0)	388 (3.1)	2 (0.5)	
ADP + AA + ASE + PHY + PSY	107 (0.8)	105 (0.8)	2 (0.5)	
ADP + AA + MSB + PHY + PSY	33 (0.3)	31 (0.2)	2 (0.5)	
ADP + AP	81 (0.6)	79 (0.6)	2 (0.5)	
ADP + AP + AA + OT	73 (0.6)	71 (0.6)	2 (0.5)	
ADP + AP + AA + CM	36 (0.3)	34 (0.3)	2 (0.5)	
ADP + AP + AA + CM + PSY	67 (0.5)	65 (0.5)	2 (0.5)	
ADP + AP + AA + CM + OT + PHY	32 (0.2)	30 (0.2)	2 (0.5)	
ADP + AP + AA + HYP + PHY + PSY	65 (0.5)	63 (0.5)	2 (0.5)	
ADP + AP + AA + HYP + CM + OT + PHY + PSY	17 (0.1)	15 (0.1)	2 (0.5)	
ADP + AP + AA + ASE + OT + PHY	79 (0.6)	77 (0.6)	2 (0.5)	
ADP + AP + AA + ASE + OT + T3	18 (0.1)	16 (0.1)	2 (0.5)	
ADP + AP + AA + ASE + CM	40 (0.3)	38 (0.3)	2 (0.5)	
ADP + AP + AA + ASE + CM + PHY	52 (0.4)	50 (0.4)	2 (0.5)	
ADP + AP + AA + ASE + CM + T3 + PHY + PSY	4 (0.0)	2 (0.0)	2 (0.5)	
ADP + AP + AA + ASE + CM + OT + PHY	32 (0.2)	30 (0.2)	2 (0.5)	
ADP + AP + AA + ASE + HYP + PHY	21 (0.2)	19 (0.2)	2 (0.5)	
ADP + AP + AA + ASE + HYP + CM + PSY	13 (0.1)	11 (0.1)	2 (0.5)	
ADP + AP + AA + ASE + HYP + CM + OT + PHY + PSY	24 (0.2)	22 (0.2)	2 (0.5)	
ADP + AP + AA + MSB + ASE	29 (0.2)	27 (0.2)	2 (0.5)	
ADP + AP + AA + MSB + ASE + T3	5 (0.0)	3 (0.0)	2 (0.5)	
ADP + AP + AA + MSB + ASE + OT	18 (0.1)	16 (0.1)	2 (0.5)	
ADP + AP + AA + MSB + ASE + HYP + PHY + PSY	3 (0.0)	1 (0.0)	2 (0.5)	

### Results of ML models

#### Predictive performance

**Fig. 4:**
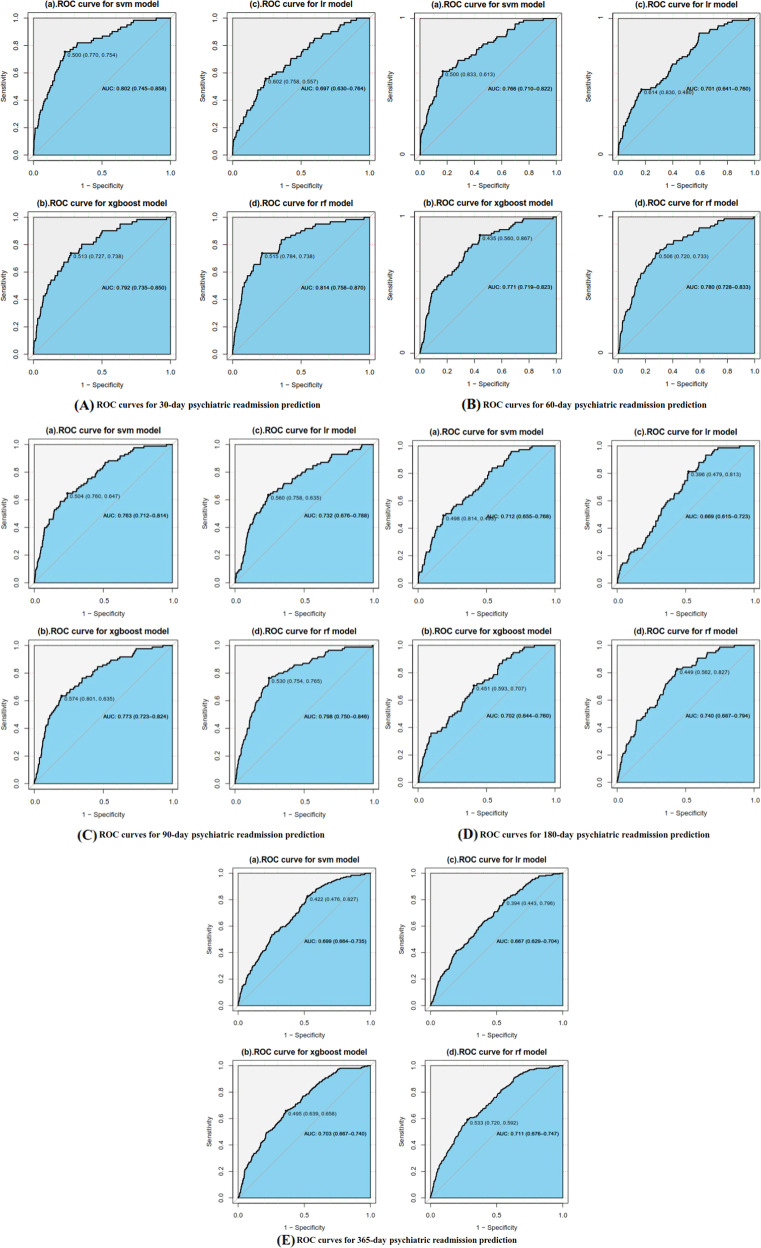
Model performance in the holdout test dataset for the 30, 60, 90, 180, and 365-day psychiatric readmission prediction. **A** ROC curves for 30-day psychiatric readmission prediction. **B** ROC curves for 60-day psychiatric readmission prediction. **C** ROC curves for 90-day psychiatric readmission prediction. **D** ROC curves for 180-day psychiatric readmission prediction. **E** ROC curves for 365-day psychiatric readmission prediction.

When predicting psychiatric readmission after the initial MDD hospitalization, the best predictive performance of our models decreased over follow-up time after discharge. As shown in Table 4 and Fig. 4 (the initial train-test split in Supplementary Information 1.5 for example), the best performance of each ML algorithm was compared. The AUC of 30-day psychiatric readmission in the holdout test dataset was 0.814 (0.758–0.87) and decreased to 0.711 (0.676–0.747) of 365-day psychiatric readmission. Since the data used in our models were patients’ information generated during hospitalization, the performance relative to time elapsed since discharge would decrease, probably due to less available information. Specifically, the best performance was achieved by RF for 30-day (with 66 features), 60-day (with 83 features), 90-day (with 85 features), 180-day (with 94 features), and 365-day (with 94 features) psychiatric readmission predictions. See Supplementary Information 2.2 for details on the results of predictive performance of other three train-test splits and the results of DeLong’s test. Results show that almost all AUCs obtained from other train-test splits were not significantly different from the AUCs obtained from the initial train-test split.

**Table 4:** Performance of the prediction models of 30-, 60-, 90-, 180-, and 365-day psychiatric readmission.

Performance of testing data	Days of follow-up	AUC (90% CI)	Threshold (Determined by Youden index)	Sensitivity	Specificity	PPV	NPV
*SVM*	30 days	0.802 (0.745–0.858)	0.500	0.754	0.770	0.096	0.990
	60 days	0.766 (0.710–0.822)	0.500	0.613	0.833	0.140	0.980
	90 days	0.763 (0.712–0.814)	0.504	0.647	0.760	0.132	0.975
	180 days	0.712 (0.655–0.768)	0.498	0.493	0.814	0.184	0.950
	365 days	0.699 (0.664–0.735)	0.422	0.827	0.476	0.145	0.962
*Xgboost*	30 days	0.792 (0.735–0.850)	0.513	0.738	0.727	0.080	0.988
	60 days	0.771 (0.719–0.823)	0.435	0.867	0.560	0.081	0.990
	90 days	0.773 (0.723–0.824)	0.574	0.635	0.801	0.152	0.975
	180 days	0.702 (0.644–0.760)	0.451	0.707	0.593	0.129	0.960
	365 days	0.703 (0.667–0.740)	0.495	0.658	0.639	0.163	0.962
*Logistic regression*	30 days	0.697 (0.630–0.764)	0.602	0.557	0.758	0.069	0.981
	60 days	0.701 (0.641–0.760)	0.614	0.480	0.830	0.111	0.973
	90 days	0.732 (0.676–0.788)	0.560	0.635	0.758	0.129	0.974
	180 days	0.669 (0.615–0.723)	0.396	0.813	0.479	0.117	0.968
	365 days	0.667 (0.629–0.704)	0.394	0.796	0.443	0.133	0.953
*Random forest*	30 days	0.814 (0.758–0.870)	0.515	0.738	0.784	0.099	0.989
	60 days	0.780 (0.728–0.833)	0.506	0.733	0.720	0.104	0.984
	90 days	0.798 (0.750–0.846)	0.530	0.765	0.754	0.149	0.983
	180 days	0.740 (0.687–0.794)	0.449	0.827	0.562	0.138	0.974
	365 days	0.711 (0.676–0.747)	0.533	0.592	0.720	0.185	0.943

#### Important features

**Fig. 5:**
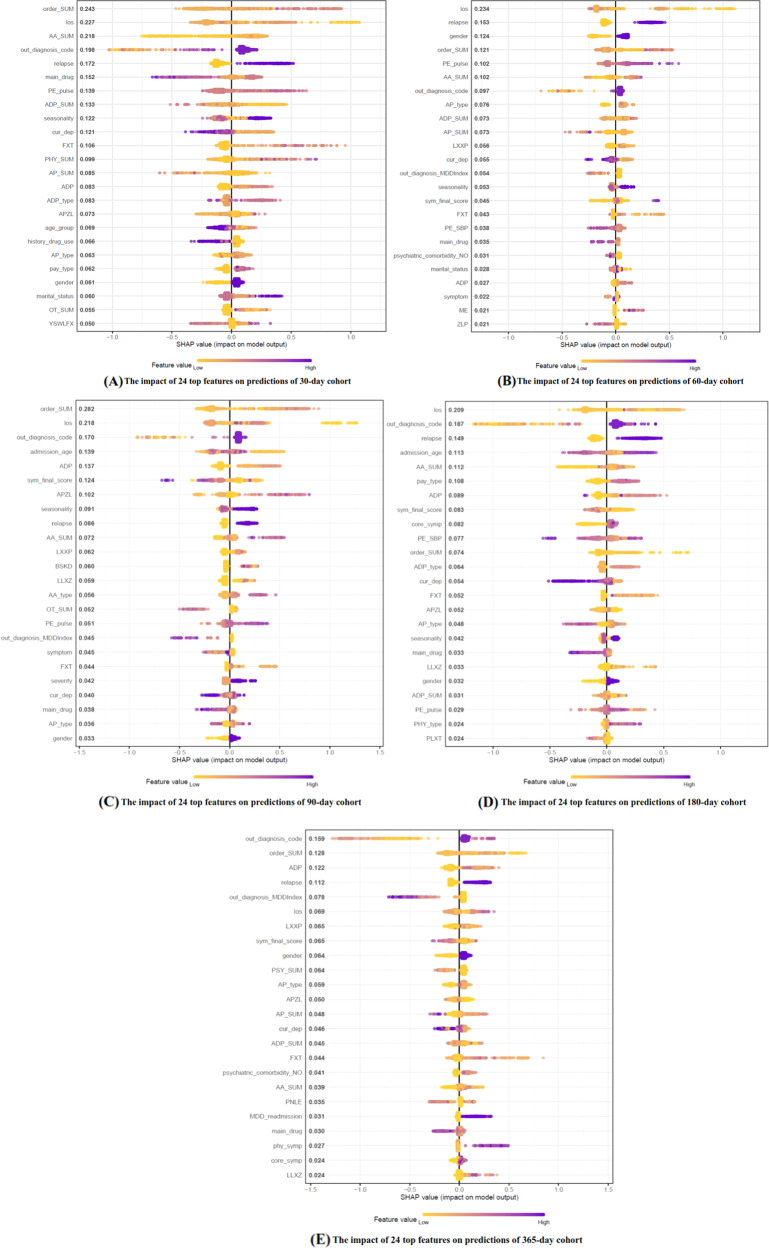
The impact of the 24 top important features on predictions. **A** The impact of 24 top features on predictions of 30-day cohort. **B** The impact of 24 top features on predictions of 60-day cohort. **C** The impact of 24 top features on predictions of 90-day cohort. **D** The impact of 24 top features on predictions of 180-day cohort. **E** The impact of 24 top features on predictions of 365-day cohort.

When considering the overall contribution of each of the features, the graphical explanation of 24 top important features in Xgboost models with respect to each prediction-modeling cohort were shown in Fig. 5. The Y-axis represents the importance of the features in making predictions, ranked in descending order. The X-axis represents the SHAP value. Each dot represents the impact of a feature on the readmission or non-readmission prediction for one patient in the training set. To be more specific, dots to the right (a SHAP value > 0) mean that patients with feature values contributed to a class “1” (readmission) prediction whereas dots to the left (a SHAP value < = 0) mean that patients with feature values contributed to a class “0” (non-readmission) prediction. The color from yellow to purple represents the feature’s value from low to high. The color coding shows how the difference in the values of each feature affected the results of the model. For example, low median values of continuous features like length of stay (los), sum of prescribed orders of antidepressants and antipsychotics, and treated in the care units of pediatrics and geriatrics were more strongly predictive of 30-day psychiatric readmission. High median values of prescribed orders (including total orders, anxiolytics, fluoxetine hydrochloride dispersible tablets, physiotherapies, olanzapine tablets, alprazolam tablets) and pulse, young female patients with recurrent MDD and without history of drug use, paid by medical insurances and admitted in winter generally drive predictions towards 30-day psychiatric readmission (Fig. 5A).

For comparison, Fig. 5B–E illustrate the contributions for the features in the 60-, 90-, 180-, and 365-day psychiatric readmission prediction models, respectively. There are two marked differences: the combination and ranking of the 24 top important features that drive the prediction process behind the Xgboost models change with the follow-up time; the contribution of the same feature (including the relative importance and the prediction direction) can be different with the changing follow-up times. Some features like los, symptom frequency score, sum of prescribed orders of antipsychotics (anxiolytics, antidepressants), principal diagnosis, the number of prescribed orders of alprazolam and clonazepam were important in all prediction scenarios, while there will be unique features for specific scenarios. It is not surprising that los has the greatest impact on the 30-, 60-, and 90-day psychiatric readmission predictions, with shorter los driving the predictions towards readmission and longer los driving the predictions towards non-readmission. This is in keeping with the fact that los is the variable that potentially reflects the thoroughness and effects of hospitalization treatment, and shorter los can significantly affect the short-term readmission risk. Short-term (30-, 60-, and 90-day) and medium-term (180-day) readmission risk can be affected most by the thoroughness and effects of treatment during the index admission, while the principal diagnosis itself and recurrence of symptoms are more likely to affect the long-term (365-day) readmission risk. Amount and type of ADP, AP, and AA used provided great impact on the readmission predictions in all prediction scenarios, using more anxiolytics driving predictions towards readmission while using less antidepressants driving predictions towards readmission.

**Fig. 6:**
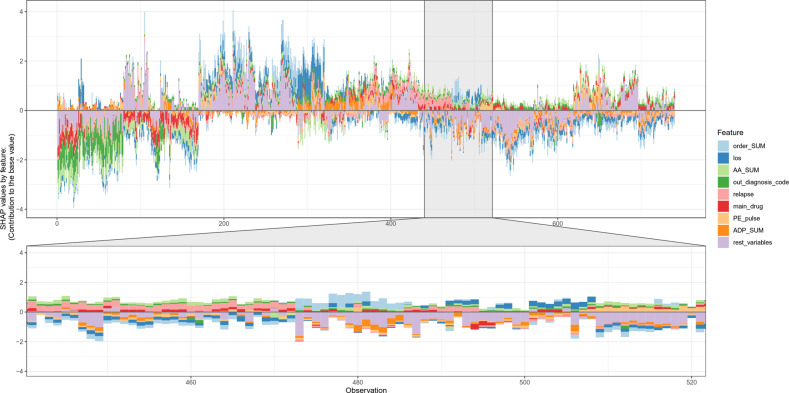
Force plot of all features at individual patient level for the holdout test dataset of the 30-day cohort.

**Fig. 7:**
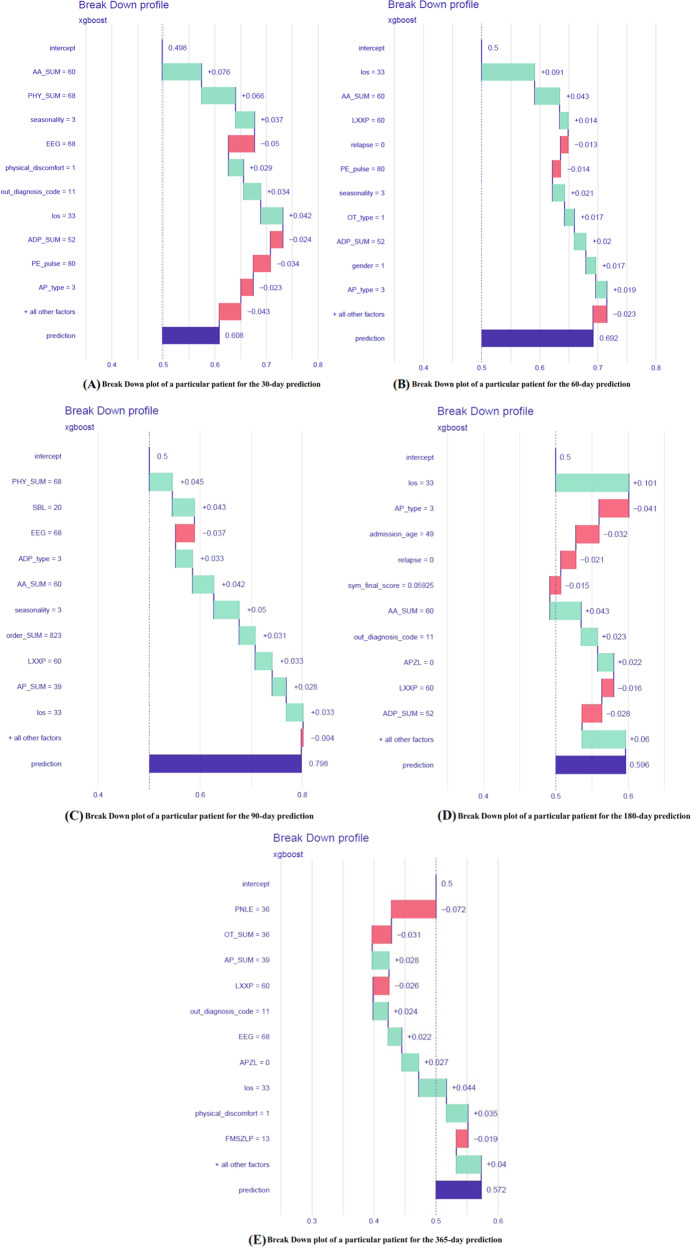
Impact of input features on 30-, 60-, 90-, 180-, and 365-day psychiatric readmission prediction for a single patient. **A** Break Down plot of a particular patient for the 30-day prediction. **B** Break Down plot of a particular patient for the 60-day prediction. **C** Break Down plot of a particular patient for the 90-day prediction. **D** Break Down plot of a particular patient for the 180-day prediction. **E** Break Down plot of a particular patient for the 365-day prediction.

When considering the relative importance of all features on the 30-day predictions for the holdout test dataset at individual patient level, the results were shown in Fig. 6. The force plot provides a graphical explanation of the 8 top important features’ contribution to the prediction for each individual patient. For some patients, the contribution of these 8 top important features exceeds half of the contribution of all other features. The same feature’s contribution to different individual patients can be different. See Supplementary Information 2.3 for the results of 60-, 90-, 180-, and 365-day cohorts. Moreover, the readmission risk prediction can also be explained at the five given follow-up times for a particular patient; we illustrated a case from the holdout test set (Fig. 7). The patient was a 49-year-old female diagnosed as first episode MDD without comorbidity and without prior hospitalization history at WCH. The patient readmitted with a MDD diagnosis within 30 days after discharge of her initial MDD hospitalization. In this case, the total number of prescribed orders of anxiolytics and physiotherapies drives the prediction towards psychiatric readmission throughout, whereas the total number of prescribed orders of electroencephalographic (EEG) biofeedback therapy is the most important feature pulling the prediction towards non-readmission within 30 days after discharge, along with the number of prescribed orders of antidepressants and pulse. In the same patient, the combination and ranking of the most important features change with the follow-up time. Some features can drive the prediction towards readmission at one follow-up time and towards non-readmission at other time windows (e.g., the total number of prescribed orders of antidepressants and the number of prescribed orders of clonazepam).

#### Feature interactions

**Fig. 8:**
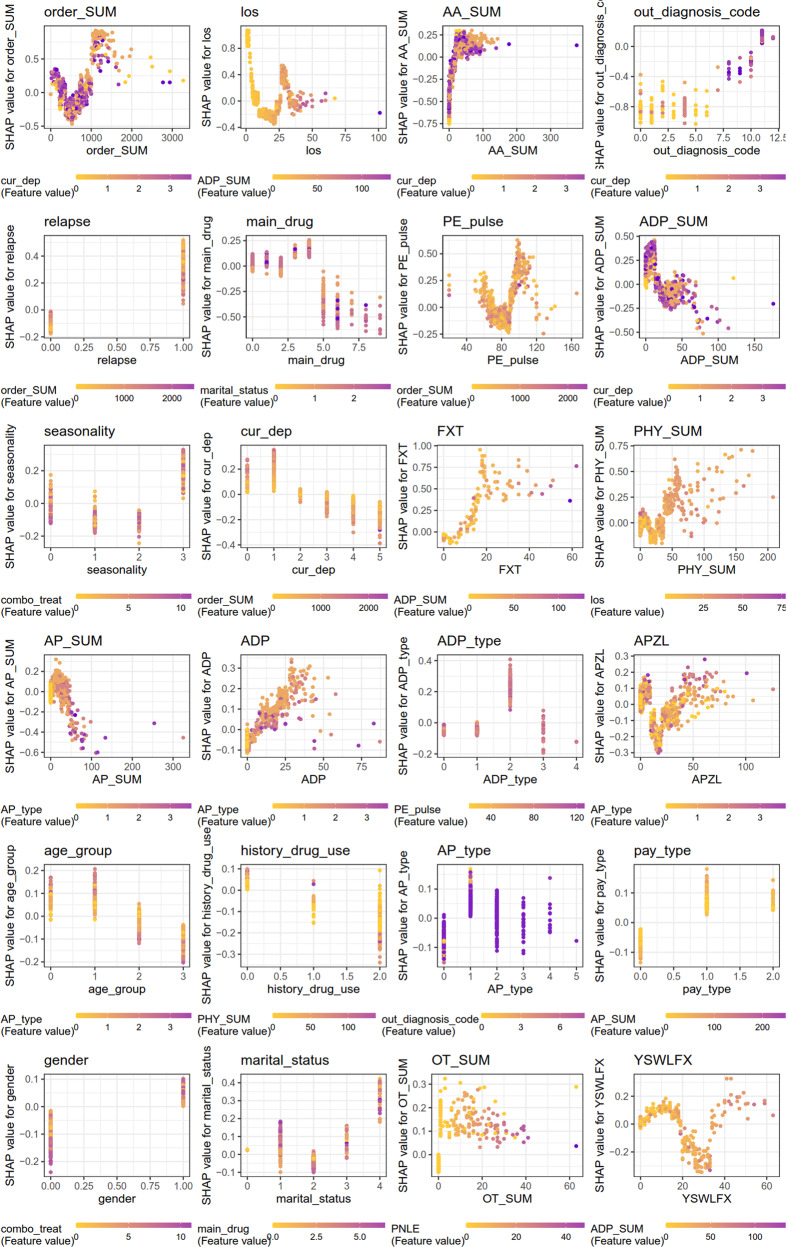
Feature interactions of 24 top important features of the 30-day cohort.

We further detail the importance of 24 top features in each model and provide a visual example of how they interact for the 30-day prediction (Fig. 8 and Supplementary Information 2.4). Varying trend of the SHAP value of each feature was also shown in the figures. In each figure, the X-axis shows the feature values, and the *Y* axis shows the SHAP value of the specific feature on the *X* axis. The color represents the value of the feature that interacts with the specific feature on the *X* axis. When considering the los, the SHAP value (relative risk of 30-day readmission prediction) first decreases with increasing values of los and then increases at the median value of readmitted patients. The varying curve of los for patients treated with different total number of prescribed orders of antidepressants can be differentiable, and the range of relative risks of los is quite wide and can be either positive or negative effect. Due to feature interactions, the relative risk of los on the readmission predictions decreases with the increasing of the total number of prescribed orders of antidepressants. When considering the total number of prescribed orders of anxiolytics, using more anxiolytics is associated with a higher relative risk of readmission. Moreover, gender interacts with the combination of all therapy types. Female patients have higher risk of 30-day psychiatric readmission than male patients. For female patients, using the combination of antipsychotics, anxiolytics, antidepressants, and other drugs would achieve lower risk of 30-day psychiatric readmission than using other treatment regimens. The opposite is true for male patients. Age interacts with the number of treated antipsychotic types. Younger patients (under 35 years old) have higher risk of 30-day psychiatric readmission than older patients (over 35 years old). For younger patients, more total number of treated antipsychotic types achieved higher risk, while less total number of treated antipsychotic types achieved higher risk for older patients.

## Discussion

In this study, we developed a risk prediction model providing individual 30-, 60-, 90-, 180-, and 365-day psychiatric readmission predictions after discharge of the initial major depression hospitalization, respectively. The current study is the first of its kind to investigate the role of multiple predictors extracted from EMR for rehospitalization modeling about 13,000 MDD individuals. Overall, our model provided a performance of AUC of 0.814 (0.758–0.87) for the 30-day psychiatric readmission prediction, AUC 0.78 (0.728–0.833) for the 60-day psychiatric readmission prediction, AUC 0.798 (0.75–0.846) for the 90-day psychiatric readmission prediction, AUC 0.740 (0.687–0.794) for the 180-day psychiatric readmission prediction, and AUC 0.711 (0.676–0.747) for the 365-day psychiatric readmission. Of clinical importance is whether this level of performance is sufficient for clinical use and how our model can be used to inform clinical decision making. Our model had comparable performance to a previous EMR-based predictive model of psychiatric readmission within 30 days for patients with a principal diagnosis of MDD in USA [7], in terms of the AUC (0.814 vs. 0.784). Their model based on baseline information (socioeconomic and clinical features like age, gender, insurance and Charlson comorbidity index) and top-1000 words extracted from discharge summaries to represent patients’ symptoms and characteristics of illness course contributed to an AUC of 0.682 and a sensitivity of 0.213; our model also included baseline information and top-20 words extracted from chief complaint to represent patients’ symptoms but resulting in a higher AUC and sensitivity.

Beyond prediction, it is also possible that identifying particularly high-risk factors and individuals will facilitate the development of interventions to reduce readmission risk in particular patient groups. Established psychiatric readmission risk factors include age, marital status, source of payment, unemployment [10, 40–42], shorter length of stay, greater psychiatric comorbidity such as substance use or eating disorders, general medical illness such as infection or dementia and markers of greater depression severity [7], level of symptoms at discharge and history of previous admissions [23], electroconvulsive therapy associated with reduced short-term psychiatric readmission [11], and more. Comparison among international studies is limited as service varies in different countries, and different populations are examined, results of psychiatric readmission risk factors appear to be consistent only for a few factors. The results of the present study add evidence that psychiatric comorbidity and other medical comorbid illnesses may have less impact psychiatric readmission risk of MDD patients. Markers of depression severity and symptoms (recurrence of the symptoms, symptom frequency score, combination of key symptoms, the number of core symptoms and physical symptoms), along with age, gender, type of payment, length of stay, treatment patterns such as the use of anxiolytics, antipsychotics, antidepressants, physiotherapy, and psychotherapy, and vital signs like pulse and SBP, may improve prediction of readmission. Our findings build on this work as well as provide preliminary evidence for the predictive ability of vital signs on psychiatric readmission.

As few studies are yet to consider a diverse range of clinically easy-collected features for readmission modeling, the predictive ability of the included features, as well as their interactions is of interest. Our model was made explainable by using SHAP and Break Down, and the top features driving psychiatric readmission prediction were identified both for an individual and the full holdout test dataset. Importantly, in the analysis of individual readmission predictions over follow-up time, we found that one feature can drive the prediction towards readmission at one follow-up time and towards non-readmission at another. The contribution of each feature to the predicted outcome changes nonlinearly with its value. Overall, we note that features interacted in a complex manner, and certain features could compensate for one another. An example was the positive effect of the used number of antipsychotics on readmission prediction could be aggravated by using more type of antipsychotics. In addition, obvious threshold effect was observed in the changing curve of SHAP value, such as the total number of orders and los (Fig. 8). Albeit some input features appear statistically heterogeneous in readmissions and non-readmissions, they did not have the anticipated impact on the predictions. For instance, history of smoking and drinking, whether a patient has symptoms of afraid and dizziness, and the used number of some drugs like sulpiride, tandospirone, ezopiclone, and so on, did not alter predictions much. A possible explanation for these counter-intuitive associations might be that the model was unable to learn the true importance of the conditions due to the small prevalence in the training dataset.

The predicted outcome varies with the follow-up time, and it encompasses short, medium, and long-term readmission risks. Patients who are readmitted within 30 days are probably quite different from patients who are readmitted after 30 days (Supplementary Information 2.5). Thus, the model adapts to account for the changing nature of the predicted outcome, making it more useful than one-off predictions. Using such a model for decision support gives the clinician dynamic information of the patient’s risk of psychiatric readmission and the specific features pulling towards readmission. This may help to establish personalized interventions that change with follow-up time. As aforementioned, short- and medium-term readmission risks can be affected most by the thoroughness and effects of treatment during the index admission, while the principal diagnosis itself and recurrence of symptoms are more likely to affect the long-term readmission risk.

Interpretation of meaningful findings and its implementations for prevention and early intervention for 30-day psychiatric readmission are presented as follows:

### Sociodemographic and clinical features

Considering sociodemographic features, in the current study, female, younger-aged (under 17 years old) student, older-aged (over 60 years old) retired and unemployed patients, unmarried and widowed patients, with either public or private medical insurance, had an observed higher relative risk of 30-day psychiatric readmission compared to other demographics. Social factors appear to be influential in the condition of MDD, indicating that population-level outcomes for MDD might be best improved through broad-based societal interventions. Consistent with previous studies, our model demonstrated that patients with a diagnosable severe MDD captured in ICD-10 codes were more likely to be readmitted. Alcohol- and drug-related problems and non-compliance with medication have been found to be the most important factors associated with frequency of hospitalizations [43]. In this study, more patients who were not readmitted described a history of smoking and drinking. Overall, 672 patients (with 615 males, 91.5%) described smoking status and only 20 of them readmitted; 512 patients (with 449 males, 87.7%) described drinking status and only 13 of them readmitted. Smoking and drinking that mainly occur in male patients may relieve depression. Shorter los can significantly increase the short- and medium-term readmission risk. Moreover, readmitted patients showed significantly different vital signs like pulse and SBP. Individual-level interventions may also be of great value in readmission risk prevention. For example, appropriately extending the los, and providing post-discharge medication and psychological treatment supervision, the efforts and help from the community would be very helpful.

### Symptoms at admission

Prior studies found that symptoms may remain a risk factor for readmission over the short and long term (as the illness progresses), consistent results were obtained when patient self-reported chief complaints were studied. Specifically, symptoms like mood-down, flustered, dizziness, physical discomfort, afraid and presented recurrence of these symptoms showed higher risk of 30-day psychiatric readmission. According to the Guidelines for prevention and treatment of MDD in China, the manifestations of depressive episodes can be divided into three categories: core symptom group, psychological symptom group and physical symptom group. Overall, 63% of the study population had at least one core symptom. The proportions of patients with at least one psychological symptom and at least one physical symptom were 27% and 53.4%, respectively. The fundamental findings were that patients with more core and psychological symptoms were more likely to be readmitted and should be paid more attention. Readmitted patients were more likely to have combinations of recurrent mood-down, suicide ideation, psychotic symptoms, while non-readmitted patients were more likely to have combinations of mood-down and physical symptoms (bad sleep, dizziness, fatigue, etc.).

### Treatment provided during the index admission

The majority of MDD patients (96.7%) involved treatment with at least one drug type of antidepressants (ADP), antipsychotics (AP), anxiolytics (AA), mood stabilizers (MSB), anti-side effects drugs (ASE), new hypnotics (HYP), β receptor blockers (OT), hormonal drugs (T3), Chinese patent medicines (CM), or one type of physiotherapy (PHY) and psychotherapy (PSY). The most frequent used treatment pattern was the combination of ADP, AP, AA, PHY, and PSY (7.3%), followed by the combination of ADP, AP, AA, and PSY (4.6%), the combination of ADP and AA (4.5%), and the combination of ADP, AP, AA, ASE, PHY, and PSY (4.0%). The remaining 3.3% of patients did not undergo any antidepressant therapy during the index admission stay as they were treated in the orthopedics, urology and other non-mental health care units and had a supplementary diagnosis of MDD. Selective serotonin reuptake inhibitors (SSRIs) (i.e., Fluoxetine with a 9.96% utilization rate [UR], Paroxetine with a 22.14% UR, Sertraline with a 27.31% UR) and serotonin-norepinephrine reuptake inhibitors (SNRIs) (i.e., Venlafaxine with a 25.73% UR) were the most frequently administered antidepressant medications, which was in line with the literature [2]. Antipsychotics like Olanzapine (35.68% UR) and Quetiapine fumarate (18.49% UR), anxiolytics like Alprazolam (57.87% UR), Clonazepam (39.08% UR) and Lorazepam (22.27% UR), and β receptor blocker like Propranolol (14.23% UR) were also utilized frequently. Interestingly, the usage frequency of Fluoxetine, Clonazepam, Olanzapine, and Alprazolam presented strong association with psychiatric readmission risk, and it is possible that patients treated with these drugs had a more severe form of illness requiring augmentation with antipsychotic and anxiolytic medications, placing them at a greater risk of rehospitalization. In addition, thyroid medication (T3) use was negatively associated with rehospitalization. Findings demonstrated that, the more complex the combination of psychiatric therapy was, the more likely the patient to be readmitted with a psychiatric diagnosis. The more type of drugs or physiotherapy and psychotherapy used in combination, the higher the patient’s readmission risk, highlighting the need for effective and efficient interventions that can target on patient groups with specific patterns.

There are several limitations of this study. First, the whole study was retrospectively conducted using a single and unbalanced sample collected from a large hospital’s EMR data in Southwest China. There is most probably a selection bias that hampers the generalizability of the ML algorithms trained in this dataset, although all the correct procedures to avoid overfitting were taken. External validation based on a larger sample size and multi-site samples could have decreased the uncertainty of some of our models, particularly regarding the performance of models resampling the positive and negative classes. As we were unable to identify a similar clinical cohort available for collaboration from other centers, the generalizability and portability of our methods could not be verified. Such validation would be a necessary next step before attempting to disseminate risk prediction models beyond this Chinese health system. The health system examined here is not a closed system, it is possible that individuals could be readmitted to another hospital and go undetected. This corollary may also be calibrated by the low incidence of readmission compared to those reported in prior studies. Particularly, the overall incidence of readmission was 16.25% within 10 years, less than the incidence of 22.26% reported for a Scottish Health Survey sample with a median follow-up of 4.5 years per participant [27], and 29.17% in a year reported by [23]. Consequently, the outcome measure of partial patients who were not readmitted with a psychiatric diagnosis at WCH might be mislabeled; namely, some non-readmitted patients should have been labeled as positive cases. Such mislabeling would tend to decrease the discrimination of predictive models, so the performance estimates provided here are likely conservative. Additionally, most of the false positives possess many strong risk factors, indicating these individuals share characteristics with those individuals who were readmitted. These patients should be reached low-cost interventions to mitigate psychiatric readmission risk in subsequent years. Clinical evaluation is particularly important in this regard and increasing emphasis on risk quantification and stratification may provide another means of improving prediction and saving intervention costs. Secondary, this study only involved predictors generated before discharge, another valuable comparison would consider patients’ information such as level of symptoms at discharge and post-discharge care, which have been acknowledged as important risk factors related to higher chance to be readmitted in patients with MDD. Thirdly, although we have made the ML models explainable, algorithm bias cannot be resolved because our models have no underlying causal structure.

Clinically, we believe that the limitations indicate that our model approach would be insufficient for directly using as a clinical tool without additional investigation. However, we believe that because our model was constructed directly from EMR data, integration into an EMR-based systemwide clinical decision support program would be more practical than if the model were created using data that needed to be collected outside the EMR.

In conclusion, we developed an explainable ML model for individual 30-, 60-, 90-, 180-, and 365-day psychiatric readmission prediction after discharge of the initial major depression hospitalization from a total dataset of about 13,000 patients from a large hospital in Chengdu, China. The presented findings suggest that the combination of ML techniques with EMR data may lead to an increase in prognostic certainty compared to chance level. Model interpretation showed that input features can interact and compensate for one another and can pull towards readmission at one follow-up time and towards non-readmission at another. None of these observations can be obtained from current static studies. Yet, before this kind of model can be used as a clinically actionable tool, continued research is required, and the results need to be confirmed in external validation.

## Supplementary information

Supplementary Information

## Data Availability

The data used to support the findings of this manuscript are restricted by the West China Hospital, to protect patient privacy and avoid legal and ethical risks. Data are available from West China Hospital for researchers who meet the criteria for access to confidential data.

## References

[1] H Whiteford, Addressing the burden of mental, neurological, and substance use disorders: Key messages from disease control priorities, 3rd edition, Lancet. 2015. 10.1016/S0140-6736(15)00390-6 26454360

[2] Citrome L, Jain R, Tung A, Landsman-Blumberg P, Kramer K, Ali S (2019). Prevalence, treatment patterns, and stay characteristics associated with hospitalizations for major depressive disorder. J Affect Disord.

[3] Nordentoft M, Mortensen P, Pedersen C (2011). Absolute risk of suicide after first hospital contact in mental disorder. Arch Gen Psychiatry.

[4] Cearns M, Opel N, Clark S, Kaehler C, Thalamuthu A, Heindel W (2019). Predicting rehospitalization within 2 years of initial patient admission for a major depressive episode: a multimodal machine learning approach, Translational. Psychiatry.

[5] Cipriani A, Furukawa TA, Salanti G, Geddes JR, Higgins JP, Churchill R (2009). Comparative efficacy and acceptability of 12 new-generation antidepressants: a multiple-treatments meta-analysis. Lancet.

[6] Rost K, Nutting P, Smith JL, Elliott CE, Dickinson M (2002). Managing depression as a chronic disease: a randomised trial of ongoing treatment in primary care. BMJ.

[7] Rumshisky A, Ghassemi M, Naumann T, Szolovits P, Castro V, McCoy T (2016). Predicting early psychiatric readmission with natural language processing of narrative discharge summaries. Transl Psychiatry.

[8] National Research Council: Committee on A Framework for Developing a New Taxonomy of Disease. Toward precision medicine: building a knowledge network for biomedical research and a new taxonomy of disease, National Academic Press: Washington DC, USA; 2011. 22536618

[9] Wang R, Tang S, Shaw I, Feng Z, Chen Z, Luo Y, et al. Integrated decision-making model for community-based rehabilitation service utilisation among persons with severe mental illness in China: protocol for a cross-sectional, mixed-methods study. BMJ Open. 2018;8:e021528. 10.1136/bmjopen-2018-021528 PMC6303639 30530575

[10] Hanrahan NP, Bressi S, Marcus SC, Solomon P (2016). Examining the impact of comorbid serious mental illness on rehospitalization among medical and surgical inpatients. Gen Hospital Psychiatry.

[11] Slade EP, Jahn DR, Regenold WT, Case BG (2017). Association of electroconvulsive therapy with psychiatric readmissions in US hospitals. JAMA Psychiatry.

[12] Germack HD, Caron A, Solomon R, Hanrahan NP (2018). Medical-surgical readmissions in patients with co-occurring serious mental illness: a systematic review and meta-analysis. Gen Hospital Psychiatry.

[13] Donisi V, Tedeschi F, Salazzari D, Amaddeo F (2016). Pre- and post-discharge factors influencing early readmission to acute psychiatric wards: implications for quality-of-care indicators in psychiatry. Gen Hospital Psychiatry.

[14] Byrne SL, Hooke GR, Page AC (2010). Readmission: a useful indicator of the quality of inpatient psychiatric care. J Affect Disord.

[15] Lauber C, Lay B, RSsler W (2006). Length of first admission and treatment outcome in patients with unipolar depression. J Affect Disord.

[16] Lyons JS, O’Mahoney MT, Miller SI, Neme J, Kabat J, Miller F (1997). Predicting readmission to the psychiatric hospital in a managed care environment: implications for quality indicators. Am J Psychiatry.

[17] Reese RL, Freedland KE, Steinmeyer BC, Rich MW, Rackley JW, Carney RM (2011). Depression and rehospitalization following acute myocardial infarction. Circulation Cardiovascular Qual Outcomes.

[18] Lin CH, Chen YS, Lin CH, Lin KS (2010). Factors affecting time to rehospitalization for patients with major depressive disorder. Psychiatry Clin Neurosci.

[19] Masters GA, Baldessarini RJ, Engr D, Centorrino F (2014). Factors associated with length of psychiatric hospitalization. Compr Psychiatry.

[20] Vink D, Aartsen MJ, Schoevers RA (2008). Risk factors for anxiety and depression in the elderly: a review. J Affect Disord.

[21] Mandl KD (2009). Longitudinal histories as predictors of future diagnoses of domestic abuse: modelling study. Br Med J.

[22] Perlis HRoy (2013). A clinical risk stratification tool for predicting treatment resistance in major depressive disorder. Biol Psychiatry.

[23] Baeza FLC, da Rocha NS, Fleck MPA. Readmission in psychiatry inpatients within a year of discharge: The role of symptoms at discharge and post-discharge care in a Brazilian sample. Gen Hosp Psychiatry. 2018;51:63–70. 10.1016/j.genhosppsych.2017.11.008 29324277

[24] Chwastiak LA, Davydow DS, Mckibbin CL, Schur E, Burley M, McDonell MG (2014). The effect of serious mental illness on the risk of rehospitalization among patients with diabetes. Psychosomatics.

[25] Lin HC, Lee HC (2009). Psychiatrists case load volume, length of stay and mental healthcare readmission rates: a three-year population-based study. Psychiatry Res.

[26] Stephanie B, Akin A, Becci A. Predictors of admission to acute inpatient psychiatric care among children enrolled in Medicaid. Adm Policy Ment Health. 2015;42:197–208. 10.1007/s10488-014-0560-6 24841746

[27] Innes H, Lewsey J, Smith DJ (2015). Predictors of admission and readmission to hospital for major depression: a community cohort study of 52,990 individuals. J Affect Disord.

[28] Jennings JH, Digiovine B, Obeid D, Frank C (2009). The association between depressive symptoms and acute exacerbations of COPD. Lung.

[29] Lin CH, Chen MC, Chou LS, Lin CH, Chen CC, Lane HY (2010). Time to rehospitalization in patients with major depression vs. those with schizophrenia or bipolar I disorder in a public psychiatric hospital. Psychiatry Res.

[30] AlcOLu E, BaOLu M (2008). Psychological effects of earthquakes in children: prospects for brief behavioral treatment. World J Pediatrics.

[31] Zheng L, Wang O, Hao S, Ye C, Liu M, Xia M, et al. Development of an early-warning system for high-risk patients for suicide attempt using deep learning and electronic health records. Transl Psychiatry. 2020;10:1–10. 10.1038/s41398-020-0684-2 PMC7033212 32080165

[32] Young J, Kempton MJ, Mcguire P. Using machine learning to predict outcomes in psychosis. Lancet Psychiatry. 2016;3:908–9. 10.1016/S2215-0366(16)30218-8 27569527

[33] Aday L, Andersen R (1974). A framework for the study of access to medical care. Health Serv Res.

[34] Andersen R (1995). Revisiting the behavioral model and access to medical care: does it matter?. J Health Soc Behav.

[35] Sperandei S (2014). Understanding logistic regression analysis. Biochemia Med.

[36] Lundberg S, Lee S (2017). A unified approach to interpreting model predictions. Adv Neurol.

[37] Staniak M, Biecek P (2018). Explanations of model predictions with live and breakDown Packages. R J.

[38] Gosiewska A, Biecek P. Do Not Trust Additive Explanations. 2019;arXiv:1903.11420v3:1–15.

[39] Thorsen-Meyer H, Nielsen A, Nielsen AP (2020). Dynamic and explainable machine learning prediction of mortality in patients in the intensive care unit: a retrospective study of high-frequency data in electronic patient records. Lancet Digital Health.

[40] Silva NC, Bassani DG, Palazzo LS (2009). A case-control study of factors associated with multiple psychiatric readmissions. Psychiatr Serv.

[41] Bernardo AC, Forchuk C (2001). Factors associated with readmission to a psychiatric facility. Psychiatr Serv.

[42] Schmutte T, Dunn CL, Sledge WH (2010). Predicting time to readmission in patients with recent histories of recurrent psychiatric hospitalization. J Nerv Ment Dis.

[43] Thomas H, Kravitz W (1995). Predicting the ‘revolving door’ phenomenon among patients with schizophrenic, schizoaffective. Am J Psychiatry.

