# Supplementary material for: Individualized prediction of psychiatric readmissions for patients with major depressive disorder: a 10-year retrospective cohort study
Source: Transl Psychiatry. 2022 Apr 23;12:170. doi: 10.1038/s41398-022-01937-7 (PMC9035153; doi:10.1038/s41398-022-01937-7)
Supplement: Supplementary file 1 — Supplementary Information [file 41398_2022_1937_MOESM1_ESM.docx]

**Supplementary Information**

**Supplementary Information SI1:**

- 1. **Study subjects**

In 2019, our team created a research database by extracting information from the EMR of WCH. First, we searched all admission records between January 1, 2009 and December 31, 2018, with a diagnosis name including the words either “depression”, “mania”, or “bipolar disorder”. According to these admission records, we identified patients’ ID number. Then, we extracted these patients’ all (psychiatric or non-psychiatric) admission records. Finally, 36,780 admission records of 21,964 inpatients who were hospitalized and then discharged at least once between January 2009 and December 2018 were extracted. The aim of creating the database was to provide high quality EMR data for research works on “depression”, “mania”, and “bipolar disorder” (such as readmission prediction, illness trajectories analysis, differential diagnosis, etc.).

The 36,780 records include these patients’ all (psychiatric or non-psychiatric) admissions at WCH. For this study, we first cleaned 6,282 records that patients were diagnosed as any disease of non-mental disorders. Based on the remaining 30,498 records, we identified 21,964 patients’ initial admission records and their 8,534 readmission records. Based on the 21,964 initial records, we then determined 12,480 patients who had a discharge diagnosis of MDD (according to the International Classification of Disease, Tenth Revision (ICD-10, Clinical Modification Codes F32 and F33)), and 9,484 patients who had other mental disorders. We attempted to determine whether some of these 9,484 patients were admitted with MDD after the initial non-MDD admission, to enrich our sample size by considering their initial MDD admission records as our study subjects. Among them, 2,318 patients showed readmission records, of which 999 records from 697 patients were identified as MDD admission. Finally, a sample contained 13,177 MDD patients were recruited as the study population of this study and each patient’s index admission record was extracted for analyses.

- 1. **Psychiatric readmission cohort**

**1.2.1 30-day cohort**

Since the censor date of our data was December 31, 2018, the collection of admission information for all study subjects ended at December 31, 2018. Patients’ 30-day psychiatric readmission status could not be determined for those index admissions occurred between December 1, 2018 and December 31, 2018, since the follow-up cannot cover a whole month. 201 patients whose discharge date of the index admission located in December 2018 were removed from the initial cohort, and 12976 patients were included in the 30-day cohort. For each patient in the 30-day cohort, follow-up began at the date of discharge, and ended at the earliest instance of 30-day psychiatric readmission (if at all), or the 30-day follow-up end date after discharge.

**1.2.2 60-day cohort.**

Similarly, the prediction-modeling cohort of 60-day psychiatric readmission, cases in the cohort referred to patients whose discharge date of the index admission located between January 1, 2009 and October 31, 2018, to ensure a 60-day follow-up. Based on the 30-day cohort, 191 patients whose discharge date of the index admission located in November 2018 were removed, and 12785 patients were included in the 60-day cohort. For each patient in the 60-day cohort, follow-up began at the date of discharge, and ended at the earliest instance of 60-day psychiatric readmission (if at all), or the 60-day follow-up end date after discharge.

**1.2.3 90-day cohort**

The prediction-modeling cohort of 90-day psychiatric readmission, cases in the cohort referred to patients whose discharge date of the index admission located between January 1, 2009 and September 30, 2018, to ensure a 90-day follow-up. Based on the 60-day cohort, 163 patients whose discharge date of the index admission located in October 2018 were removed, and 12622 patients were included in the 90-day cohort. For each patient in the 90-day cohort, follow-up began at the date of discharge, and ended at the earliest instance of 90-day psychiatric readmission (if at all), or the 90-day follow-up end date after discharge.

**1.2.4 180-day cohort**

The prediction-modeling cohort of 180-day psychiatric readmission, cases in the cohort referred to patients whose discharge date of the index admission located between January 1, 2009 and June 30, 2018, to ensure a 180-day follow-up. Based on the 90-day cohort, 640 patients whose discharge date of the index admission located between July 1, 2018 and September 30, 2018 were removed, and 11982 patients were included in the 180-day cohort. For each patient in the 180-day cohort, follow-up began at the date of discharge, and ended at the earliest instance of 180-day psychiatric readmission (if at all), or the 180-day follow-up end date after discharge.

**1.2.5 365-day cohort**

The prediction-modeling cohort of 365-day psychiatric readmission, cases in the cohort referred to patients whose discharge date of the index admission located between January 1, 2009 and December 31, 2017, to ensure a 365-day follow-up. Based on the 180-day cohort, 956 patients whose discharge date of the index admission located between January 1, 2018 and June 30, 2018 were removed, and 11026 patients were included in the 365-day cohort. For each patient in the 365-day cohort, follow-up began at the date of discharge, and ended at the earliest instance of 365-day psychiatric readmission (if at all), or the 365-day follow-up end date after discharge.

- 1. **Unstructured data processing**

In this study, unstructured data like patients’ chief complaint was processed, including 13177 chief complaints collected at the index admission. The text data of chief complaint includes Chinese words, numbers, and punctuation marks. Chinese words record patients’ main symptoms, current mood states, the specific symptom’s duration, frequency, and severity. Sometimes, the specific symptom’s duration and frequency can be recorded as numbers. The text data was split into words by jieba package of R (Version 3.6.1 for Windows). The process is as follows:

Since we aimed at extracting key words of patients’ symptoms as precitors of psychiatric readmission, we first removed all numbers and punctuation marks by using the R function “gsub”. Second, we downloaded a Chinese stop-word dictionary from CSDN (<https://blog.csdn.net/shijiebei2009/article/details/39696571>) and some medical dictionaries from the Sogou Thesaurus (<https://pinyin.sogou.com/dict/>). The medical dictionaries included medical professional dictionary, doctor psychology dictionary, medicine name dictionary, psychology dictionary, and drug name dictionary. Moreover, we defined some stop words (such as time unit, more, less, etc.) and symptom words (such as mood-down, bad sleep, loss of interest, etc.) to supplement the downloaded dictionaries. By using the jieba package of R, the text data of each patient’s chief complaint was segmented into words, and the frequency of each word was counted. The term frequency scores were used to identify the most informative words in patients’ chief complaints and these words were referred to as features of patients’ main symptoms and current mood states (mood-down, bad sleep, loss of interest, flustered, worry, tension, upset, headache, dizziness, physical discomfort, fatigue, suicide ideation, self-harm, hallucination, less activity, chest tightness, afraid, irritability, fidget, slow response, recurrence and worsen of symptoms). The value of each of the symptom feature depends on whether the chief complaint of this patient includes the above key words or not, particularly, 1 for including the specific word and 0 for not including the specific word.

- 1. **Quality of raw data and data preprocessing**

In this study, the database consists of a representative set of items to record all information generated during a patient’s hospitalization. Various categories of features were extracted from the original medical records, such as sociodemographic information, basic information about hospitalization, vital signs based on basic body check at admission, information about past illness history and life behavior, information about treatment patterns, and text data (chief complaint, discharge summary and medical record summary). The details of recruited features were concluded in the following Table S1. Drugs, physiotherapies, and psychotherapies used in the antidepressant therapy were summarized in Table S2. We analyzed the quality of our raw data. The results are presented in the following Table S3 and Table S4.

Table S1 Features recruited into the raw data of this study

| **Variable name** | **Variable interpretation** | **Data type** | **Number of different values** | **Number of na values** | **Ratio of na values** | **Number of non-na values** | **Ratio of non-na values** | **Missing value processing** |
| --- | --- | --- | --- | --- | --- | --- | --- | --- |
| PADMNO | Patient admission number (unique for each admission record of each patient) | factor | 13176 | 0 | 0% | 13176 | 100% | / |
| gender | Gender | factor | 2 | 0 | 0% | 13176 | 100% | / |
| age_group | Age group | factor | 4 | 0 | 0% | 13176 | 100% | / |
| marital_status | Marital status | factor | 7 | 45 | 0.34% | 13131 | 99.66% | Filling with data extracted from patients' past history, current medical history, personal history in the EMR |
| job | Job status | factor | 16 | 275 | 2.09% | 12901 | 97.91% | Filling with data extracted from patients' past history, current medical history, personal history in the EMR |
| nationality | Nationality | factor | 18 | 13 | 0.10% | 13163 | 99.90% | Filling with data extracted from patients' past history, current medical history, personal history in the EMR |
| pat_type | Type of patient | factor | 7 | 0 | 0% | 13176 | 100% | / |
| pay_type | Type of payment | factor | 3 | 0 | 0% | 13176 | 100% | / |
| pat_source | Source of patient | factor | 5 | 397 | 3.01% | 12779 | 96.99% | Filling with data extracted from patients' past history, current medical history, personal history in the EMR |
| hometown | Province of hometown for each patient | factor | 35 | 0 | 0% | 13176 | 100% | / |
| in_year | Year at admission | factor | 9 | 0 | 0% | 13176 | 100% | / |
| seasonality | Seasonality at admission | factor | 4 | 0 | 0% | 13176 | 100% | / |
| out_diag_code | Principal diagnosis code | factor | 1226 | 0 | 0% | 13176 | 100% | / |
| out_diag_name | Principal diagnosis name | factor | 1150 | 0 | 0% | 13176 | 100% | / |
| out_diag_code1 | Supplementary diagnosis code 1 | factor | 1516 | 4808 | 36.49% | 8368 | 63.51% | The missing means that patients have no relevant diagnoses, so no processing is required for these variables |
| out_diag_name1 | Supplementary diagnosis name 1 | factor | 1414 | 4808 | 36.49% | 8368 | 63.51% |  |
| out_diag_code2 | Supplementary diagnosis code 2 | factor | 1246 | 7615 | 57.79% | 5561 | 42.21% |  |
| out_diag_name2 | Supplementary diagnosis name 2 | factor | 1165 | 7615 | 57.79% | 5561 | 42.21% |  |
| out_diag_code3 | Supplementary diagnosis code 3 | factor | 977 | 9315 | 70.70% | 3861 | 29.30% |  |
| out_diag_name3 | Supplementary diagnosis name 3 | factor | 910 | 9315 | 70.70% | 3861 | 29.30% |  |
| out_diag_code4 | Supplementary diagnosis code 4 | factor | 773 | 10453 | 79.33% | 2723 | 20.67% |  |
| out_diag_name4 | Supplementary diagnosis name 4 | factor | 712 | 10453 | 79.33% | 2723 | 20.67% |  |
| out_diag_code5 | Supplementary diagnosis code 5 | factor | 623 | 11288 | 85.67% | 1888 | 14.33% |  |
| out_diag_name5 | Supplementary diagnosis name 5 | factor | 585 | 11288 | 85.67% | 1888 | 14.33% |  |
| out_diag_code6 | Supplementary diagnosis code 6 | factor | 488 | 11904 | 90.35% | 1272 | 9.65% |  |
| out_diag_name6 | Supplementary diagnosis name 6 | factor | 456 | 11904 | 90.35% | 1272 | 9.65% |  |
| out_diagnosis_code | Quantifying number of the principal diagnosis code | factor | 13 | 0 | 0% | 13176 | 100% | / |
| out_diagnosis_NO | Number of all diagnoses | factor | 7 | 0 | 0% | 13176 | 100% | / |
| out_diagnosis_MDDIndex | Location of MDD diagnosis in all diagnoses | factor | 7 | 0 | 0% | 13176 | 100% | / |
| severity | Severity of MDD | factor | 5 | 0 | 0% | 13176 | 100% | / |
| first_episode | Whether the MDD diagnosis is a first episode or a recurrent episode | factor | 2 | 0 | 0% | 13176 | 100% | / |
| cur_dep | Specialty care unit that the patient lived | factor | 47 | 0 | 0% | 13176 | 100% | / |
| if_trans | Whether a patient transferred to other units | factor | 2 | 0 | 0% | 13176 | 100% | / |
| in_rank | How many admissions before the index admission | factor | 19 | 0 | 0% | 13176 | 100% | / |
| main_MDD_readmission | Whether the index admission is a readmission with a principal diagnosis of MDD | factor | 2 | 0 | 0% | 13176 | 100% | / |
| main_psychiatry_readmission | Whether the index admission is a readmission with a principal diagnosis of any mental disorders | factor | 2 | 0 | 0% | 13176 | 100% | / |
| MDD_readmission | Whether the index admission is a readmission with a principal or supplementray diagnosis of MDD | factor | 2 | 0 | 0% | 13176 | 100% | / |
| psychiatry_readmission | Whether the index admission is a readmission with a principal or supplementray diagnosis of any mental disorders | factor | 2 | 0 | 0% | 13176 | 100% | / |
| cancer_comorbidity_NO | Number of cancer comorbidity | factor | 6 | 0 | 0% | 13176 | 100% | / |
| respiratory_comorbidity_NO | Number of respiratory comorbidity | factor | 6 | 0 | 0% | 13176 | 100% | / |
| circulatory_comorbidity_NO | Number of circulatory comorbidity | factor | 7 | 0 | 0% | 13176 | 100% | / |
| digestive_comorbidity_NO | Number of digestive comorbidity | factor | 7 | 0 | 0% | 13176 | 100% | / |
| nervous_comorbidity_NO | Number of nervous comorbidity | factor | 5 | 0 | 0% | 13176 | 100% | / |
| endocrine_comorbidity_NO | Number of endocrine comorbidity | factor | 6 | 0 | 0% | 13176 | 100% | / |
| psychiatric_comorbidity_NO | Number of psychiatric comorbidity | factor | 4 | 0 | 0% | 13176 | 100% | / |
| comorbidity | Whether the patient has a diagnosis of a medical comorbidity | factor | 2 | 0 | 0% | 13176 | 100% | / |
| psychiatric_comorbidity | Whether the patient has a comorbidity of psychiatric diseases | factor | 2 | 0 | 0% | 13176 | 100% | / |
| endocrine_comorbidity | Whether the patient has a comorbidity of endocrine diseases | factor | 2 | 0 | 0% | 13176 | 100% | / |
| nervous_comorbidity | Whether the patient has a comorbidity of nervous diseases | factor | 2 | 0 | 0% | 13176 | 100% | / |
| digestive_comorbidity | Whether the patient has a comorbidity of digestive diseases | factor | 2 | 0 | 0% | 13176 | 100% | / |
| circulatory_comorbidity | Whether the patient has a comorbidity of circulatory diseases | factor | 2 | 0 | 0% | 13176 | 100% | / |
| respiratory_comorbidity | Whether the patient has a comorbidity of respiratory diseases | factor | 2 | 0 | 0% | 13176 | 100% | / |
| cancer_comorbidity | Whether the patient has a comorbidity of cancer | factor | 2 | 0 | 0% | 13176 | 100% | / |
| surgery_NO | Number of surgeries during this admission | factor | 6 | 0 | 0% | 13176 | 100% | / |
| operation_NO | Number of operations during this admission | factor | 6 | 0 | 0% | 13176 | 100% | / |
| history_allergy | History of allergy | factor | 2 | 0 | 0% | 13176 | 100% | / |
| history_blood_transfusion | History of blood transfusion | factor | 2 | 0 | 0% | 13176 | 100% | / |
| history_drug_use | History of drug use | factor | 3 | 0 | 0% | 13176 | 100% | / |
| history_surgery | History of surgery | factor | 2 | 0 | 0% | 13176 | 100% | / |
| history_smoking | History of smoking | factor | 2 | 0 | 0% | 13176 | 100% | / |
| history_alcoholism | History of alcoholism | factor | 2 | 0 | 0% | 13176 | 100% | / |
| chief_complaint | Chief complaint | factor | 12340 | 645 | 4.90% | 12531 | 95.10% | Filling with data extracted from patients' discharge summary and medical record summary in the EMR |
| symptom_1 | Symptom 1: the first symptom word segmented from the chief complaint | factor | 2925 | 648 | 4.92% | 12528 | 95.08% | The missing means that patients have no relevant symptom words and duration of symptoms segmented from the chief complaint, so no processing is required for these variables |
| duration_1 | Duration of symptom 1 | factor | 168 | 654 | 4.96% | 12522 | 95.04% |  |
| symptom_2 | Symptom 2: the second symptom word segmented from the chief complaint | factor | 2475 | 1928 | 14.63% | 11248 | 85.37% |  |
| duration_2 | Duration of symptom 2 | factor | 152 | 1943 | 14.75% | 11233 | 85.25% |  |
| symptom_3 | Symptom 3: the third symptom word segmented from the chief complaint | factor | 1559 | 5457 | 41.42% | 7719 | 58.58% |  |
| duration_3 | Duration of symptom 3 | factor | 128 | 5467 | 41.49% | 7709 | 58.51% |  |
| symptom_4 | Symptom 4: the fourth symptom word segmented from the chief complaint | factor | 722 | 9811 | 74.46% | 3365 | 25.54% |  |
| duration_4 | Duration of symptom 4 | factor | 91 | 9822 | 74.54% | 3354 | 25.46% |  |
| symptom_5 | Symptom 5: the fifth symptom word segmented from the chief complaint | factor | 250 | 12330 | 93.58% | 846 | 6.42% |  |
| duration_5 | Duration of symptom 5 | factor | 63 | 12331 | 93.59% | 845 | 6.41% |  |
| symptom_6 | Symptom 6: the sixth symptom word segmented from the chief complaint | factor | 75 | 13024 | 98.85% | 152 | 1.15% |  |
| duration_6 | Duration of symptom 6 | factor | 38 | 13025 | 98.85% | 151 | 1.15% |  |
| symptom_7 | Symptom 7: the seventh symptom word segmented from the chief complaint | factor | 16 | 13151 | 99.81% | 25 | 0.19% |  |
| duration_7 | Duration of symptom 7 | factor | 15 | 13151 | 99.81% | 25 | 0.19% |  |
| symptom_8 | Symptom 8: the eighth symptom word segmented from the chief complaint | factor | 5 | 13172 | 99.97% | 4 | 0.03% |  |
| duration_8 | Duration of symptom 8 | factor | 5 | 13172 | 99.97% | 4 | 0.03% |  |
| mood | Whether the patient has a symptom of mooddown | factor | 2 | 0 | 0% | 13176 | 100% | / |
| bad_sleep | Whether the patient has a symptom of bad sleep | factor | 2 | 0 | 0% | 13176 | 100% | / |
| loss_interest | Whether the patient has a symptom of loss of interest | factor | 2 | 0 | 0% | 13176 | 100% | / |
| flustered | Whether the patient has a symptom of flustered | factor | 2 | 0 | 0% | 13176 | 100% | / |
| worry | Whether the patient has a symptom of worry | factor | 2 | 0 | 0% | 13176 | 100% | / |
| tension | Whether the patient has a symptom of tension | factor | 2 | 0 | 0% | 13176 | 100% | / |
| upset | Whether the patient has a symptom of upset | factor | 2 | 0 | 0% | 13176 | 100% | / |
| headache | Whether the patient has a symptom of headache | factor | 2 | 0 | 0% | 13176 | 100% | / |
| dizziness | Whether the patient has a symptom of dizziness | factor | 2 | 0 | 0% | 13176 | 100% | / |
| physical_discomfort | Whether the patient has a symptom of physical discomfort | factor | 2 | 0 | 0% | 13176 | 100% | / |
| fatigue | Whether the patient has a symptom of fatigue | factor | 2 | 0 | 0% | 13176 | 100% | / |
| suicide | Whether the patient has a symptom of suicide ideation | factor | 2 | 0 | 0% | 13176 | 100% | / |
| self_harm | Whether the patient has a symptom of self-harm | factor | 2 | 0 | 0% | 13176 | 100% | / |
| hallucination | Whether the patient has a symptom of hallucination | factor | 2 | 0 | 0% | 13176 | 100% | / |
| less_activity | Whether the patient has a symptom of less activity | factor | 2 | 0 | 0% | 13176 | 100% | / |
| chest_tightness | Whether the patient has a symptom of chest tightness | factor | 2 | 0 | 0% | 13176 | 100% | / |
| afraid | Whether the patient has a symptom of afraid | factor | 2 | 0 | 0% | 13176 | 100% | / |
| irritability | Whether the patient has a symptom of irritability | factor | 2 | 0 | 0% | 13176 | 100% | / |
| fidget | Whether the patient has a symptom of fidget | factor | 2 | 0 | 0% | 13176 | 100% | / |
| slow_response | Whether the patient has a symptom of slow response | factor | 2 | 0 | 0% | 13176 | 100% | / |
| relapse | Whether the patient has a recurrence of symptoms | factor | 2 | 0 | 0% | 13176 | 100% | / |
| symp_worsen | Whether the patient has a worsen of symptoms | factor | 2 | 0 | 0% | 13176 | 100% | / |
| core_symp | Core symptom group: the number of core symptoms (including mooddown and loss of interest) for each patient | factor | 3 | 0 | 0% | 13176 | 100% | / |
| psy_symp | Psychological symptom group: the number of psychological symptoms (including worry, tension, upset, suicide ideation, self-harm, hallucination, less activity, afraid, irritability, fidget and slow response) for each patient | factor | 5 | 0 | 0% | 13176 | 100% | / |
| phy_symp | Physical symptom group: the number of physical symptoms (including flustered, bad sleep, headache, dizziness, physical discomfort, fatigue and chest tightness) for each patient | factor | 6 | 0 | 0% | 13176 | 100% | / |
| combo_symptom | Combination of key symptoms for each patient | factor | 1401 | 0 | 0% | 13176 | 100% | / |
| symptom | Quantifying number of the combo_symptom | factor | 18 | 0 | 0% | 13176 | 100% | / |
| PE_subcutaneous.bleeding | Physical examination: to record whether a patient has subcutaneous bleeding | factor | 108 | 70 | 0.53% | 13106 | 99.47% | Filling with data extracted from patients' past history, current medical history, personal history in the EMR |
| PE_expression | Physical examination: to record a patient's facial expression | factor | 11 | 353 | 2.68% | 12823 | 97.32% | Filling with data extracted from patients' past history, current medical history, personal history in the EMR |
| PE_face | Physical examination: to record a patient's sickly look | factor | 9 | 353 | 2.68% | 12823 | 97.32% | Filling with data extracted from patients' past history, current medical history, personal history in the EMR |
| PE_nutrition | Physical examination: to record a patient's nutrition status | factor | 7 | 327 | 2.48% | 12849 | 97.52% | Filling with data extracted from patients' past history, current medical history, personal history in the EMR |
| PE_cooperation | Physical examination: to record whether a patient cooperates with the examination | factor | 3 | 353 | 2.68% | 12823 | 97.32% | Filling with data extracted from patients' past history, current medical history, personal history in the EMR |
| PE_consciousness | Physical examination: to record a patient's consciousness | factor | 7 | 353 | 2.68% | 12823 | 97.32% | Filling with data extracted from patients' past history, current medical history, personal history in the EMR |
| PE_gait | Physical examination: to record a patient's gait | factor | 13 | 356 | 2.70% | 12820 | 97.30% | Filling with data extracted from patients' past history, current medical history, personal history in the EMR |
| PE_body.position | Physical examination: to record a patient's body position | factor | 10 | 354 | 2.69% | 12822 | 97.31% | Filling with data extracted from patients' past history, current medical history, personal history in the EMR |
| combo_treatment | Combination of all therapy types for each patient | factor | 531 | 0 | 0% | 13176 | 100% | / |
| main_drug | Combination of drug types first used for over three days in the whole treatment path for each patient | factor | 10 | 0 | 0% | 13176 | 100% | / |
| ADP_type | Type of antidepressants used for each patient | factor | 6 | 0 | 0% | 13176 | 100% | / |
| AP_type | Type of antipsychotics used for each patient | factor | 8 | 0 | 0% | 13176 | 100% | / |
| AA_type | Type of anxiolytics used for each patient | factor | 8 | 0 | 0% | 13176 | 100% | / |
| MSB_type | Type of mood stabilizers used for each patient | factor | 5 | 0 | 0% | 13176 | 100% | / |
| ASE_type | Type of anti-side effects drugs used for each patient | factor | 8 | 0 | 0% | 13176 | 100% | / |
| HYP_type | Type of new hypnotics used for each patient | factor | 2 | 0 | 0% | 13176 | 100% | / |
| OT_type | Type of β receptor blockers used for each patient | factor | 3 | 0 | 0% | 13176 | 100% | / |
| T3_type | Type of hormonal drugs used for each patient | factor | 2 | 0 | 0% | 13176 | 100% | / |
| CM_type | Type of Chinese patent medicines used for each patient | factor | 2 | 0 | 0% | 13176 | 100% | / |
| PHY_type | Type of physiotherapies used for each patient | factor | 5 | 0 | 0% | 13176 | 100% | / |
| PSY_type | Type of psychotherapies used for each patient | factor | 2 | 0 | 0% | 13176 | 100% | / |
| order_SUM | Sum of medical orders prescribed for each patient | numeric | 1633 | 0 | 0% | 13176 | 100% | / |
| ADP_SUM | Sum of antidepressant orders prescribed for each patient | numeric | 126 | 0 | 0% | 13176 | 100% | / |
| AMTL | Number of orders of amitriptyline hydrochloride tablets prescribed for each patient | numeric | 48 | 0 | 0% | 13176 | 100% | / |
| ASXTPL | Number of orders of escitalopram oxalate tablets prescribed for each patient | numeric | 47 | 0 | 0% | 13176 | 100% | / |
| BMQ | Number of orders of imipramine hydrochloride tablets prescribed for each patient | numeric | 7 | 0 | 0% | 13176 | 100% | / |
| DLXT | Number of orders of duloxetine hydrochloride enteric-coated capsules prescribed for each patient | numeric | 54 | 0 | 0% | 13176 | 100% | / |
| DSP | Number of orders of doxepin hydrochloride tablets prescribed for each patient | numeric | 10 | 0 | 0% | 13176 | 100% | / |
| FFSM | Number of orders of fluvoxamine maleate tablets prescribed for each patient | numeric | 33 | 0 | 0% | 13176 | 100% | / |
| FPSDMLQX | Number of orders of flupentixol and meritroxine tablets prescribed for each patient | numeric | 8 | 0 | 0% | 13176 | 100% | / |
| FXT | Number of orders of fluoxetine hydrochloride dispersible tablets prescribed for each patient | numeric | 61 | 0 | 0% | 13176 | 100% | / |
| LMPM | Number of orders of clomipramine hydrochloride tablets prescribed for each patient | numeric | 44 | 0 | 0% | 13176 | 100% | / |
| MDP | Number of orders of mirtazapine tablets prescribed for each patient | numeric | 25 | 0 | 0% | 13176 | 100% | / |
| MDPP | Number of orders of mirtazapine tablets (from another manufacture) prescribed for each patient | numeric | 2 | 0 | 0% | 13176 | 100% | / |
| MPTL | Number of orders of maprotiline hydrochloride tablets prescribed for each patient | numeric | 6 | 0 | 0% | 13176 | 100% | / |
| PLXT | Number of orders of paroxetine hydrochloride tablets prescribed for each patient | numeric | 74 | 0 | 0% | 13176 | 100% | / |
| PLXTHSP | Number of orders of paroxetine hydrochloride enteric-coated sustained-release tablets prescribed for each patient | numeric | 12 | 0 | 0% | 13176 | 100% | / |
| SQL | Number of orders of sertraline hydrochloride tablets prescribed for each patient | numeric | 76 | 0 | 0% | 13176 | 100% | / |
| SYHCTQW | Number of orders of St. John's wort extract tablets prescribed for each patient | numeric | 2 | 0 | 0% | 13176 | 100% | / |
| WLFX | Number of orders of venlafaxine hydrochloride capsules prescribed for each patient | numeric | 68 | 0 | 0% | 13176 | 100% | / |
| XTPL | Number of orders of citalopram hydrobromide tablets prescribed for each patient | numeric | 42 | 0 | 0% | 13176 | 100% | / |
| YSDSPP | Number of orders of doxepine hydrochloride tablets prescribed for each patient | numeric | 41 | 0 | 0% | 13176 | 100% | / |
| YSQZT | Number of orders of trazodone hydrochloride tablets prescribed for each patient | numeric | 15 | 0 | 0% | 13176 | 100% | / |
| YSWLFX | Number of orders of venlafaxine hydrochloride sustained-release capsules prescribed for each patient | numeric | 86 | 0 | 0% | 13176 | 100% | / |
| AP_SUM | Sum of antipsychotic orders prescribed for each patient | numeric | 129 | 0 | 0% | 13176 | 100% | / |
| ADP | Number of orders of olanzapine tablets prescribed for each patient | numeric | 88 | 0 | 0% | 13176 | 100% | / |
| AHBL | Number of orders of amisulpride tablets prescribed for each patient | numeric | 23 | 0 | 0% | 13176 | 100% | / |
| ALPZ | Number of orders of aripiprazole tablets prescribed for each patient | numeric | 28 | 0 | 0% | 13176 | 100% | / |
| ALPZKBP | Number of orders of aripiprazole orally disintegrating tablets prescribed for each patient | numeric | 42 | 0 | 0% | 13176 | 100% | / |
| FMSZLP | Number of orders of quetiapine fumrate tablets prescribed for each patient | numeric | 67 | 0 | 0% | 13176 | 100% | / |
| FPDC | Number of orders of haloperidol tablets prescribed for each patient | numeric | 11 | 0 | 0% | 13176 | 100% | / |
| FPDCZSY | Number of orders of haloperidol for injection prescribed for each patient | numeric | 17 | 0 | 0% | 13176 | 100% | / |
| LBL | Number of orders of tiapride hydrochloride tablets prescribed for each patient | numeric | 44 | 0 | 0% | 13176 | 100% | / |
| LBQ | Number of orders of chlorpromazine hydrochloride tablets prescribed for each patient | numeric | 6 | 0 | 0% | 13176 | 100% | / |
| LBQ | Number of orders of chlorpromazine hydrochloride for injection prescribed for each patient | numeric | 4 | 0 | 0% | 13176 | 100% | / |
| LBZ | Number of orders of chlorpromazine prescribed for each patient | numeric | 4 | 0 | 0% | 13176 | 100% | / |
| LDP | Number of orders of clozapine tablets prescribed for each patient | numeric | 71 | 0 | 0% | 13176 | 100% | / |
| LPT | Number of orders of risperidone tablets prescribed for each patient | numeric | 60 | 0 | 0% | 13176 | 100% | / |
| PLPT. | Number of orders of paliperidone sustained-release tablets prescribed for each patient | numeric | 29 | 0 | 0% | 13176 | 100% | / |
| SBL | Number of orders of sulpiride prescribed for each patient | numeric | 25 | 0 | 0% | 13176 | 100% | / |
| SBL1 | Number of orders of sulpiride tablets prescribed for each patient | numeric | 33 | 0 | 0% | 13176 | 100% | / |
| SBLP | Number of orders of sulpiride tablets (from another manufacture) prescribed for each patient | numeric | 35 | 0 | 0% | 13176 | 100% | / |
| SBLZSY | Number of orders of sulpiride for injection prescribed for each patient | numeric | 27 | 0 | 0% | 13176 | 100% | / |
| YSQLXT | Number of orders of ziprasidone hydrochloride capsules prescribed for each patient | numeric | 7 | 0 | 0% | 13176 | 100% | / |
| ZLP | Number of orders of quetiapine fumrate tablets (from another manufacture) prescribed for each patient | numeric | 62 | 0 | 0% | 13176 | 100% | / |
| AA_SUM | Sum of anxiolytic orders prescribed for each patient | numeric | 161 | 0 | 0% | 13176 | 100% | / |
| APZL | Number of orders of alprazolam tablets prescribed for each patient | numeric | 117 | 0 | 0% | 13176 | 100% | / |
| ASZL | Number of orders of estazolam tablets prescribed for each patient | numeric | 43 | 0 | 0% | 13176 | 100% | / |
| DLHT | Number of orders of buspirone hydrochloride tablets prescribed for each patient | numeric | 35 | 0 | 0% | 13176 | 100% | / |
| DXP | Number of orders of diazepam tablets prescribed for each patient | numeric | 45 | 0 | 0% | 13176 | 100% | / |
| DXPZSJ | Number of orders of diazepam for injection prescribed for each patient | numeric | 2 | 0 | 0% | 13176 | 100% | / |
| DXPZSY | Number of orders of diazepam for injection (from another manufacture) prescribed for each patient | numeric | 15 | 0 | 0% | 13176 | 100% | / |
| LLXZ | Number of orders of lorazepam tablets prescribed for each patient | numeric | 75 | 0 | 0% | 13176 | 100% | / |
| LXXP | Number of orders of clonazepam tablets prescribed for each patient | numeric | 103 | 0 | 0% | 13176 | 100% | / |
| LXXPZSY | Number of orders of clonazepam for injection prescribed for each patient | numeric | 22 | 0 | 0% | 13176 | 100% | / |
| mdzl | Number of orders of midazolam maleate tablets prescribed for each patient | numeric | 30 | 0 | 0% | 13176 | 100% | / |
| MDZLZSY | Number of orders of midazolam for injection prescribed for each patient | numeric | 12 | 0 | 0% | 13176 | 100% | / |
| TDLT | Number of orders of tandospirone citrate tablets prescribed for each patient | numeric | 4 | 0 | 0% | 13176 | 100% | / |
| TDLTJL | Number of orders of tandospirone citrate capsules prescribed for each patient | numeric | 45 | 0 | 0% | 13176 | 100% | / |
| XXP | Number of orders of nitrazepam tablets prescribed for each patient | numeric | 8 | 0 | 0% | 13176 | 100% | / |
| YZPKL | Number of orders of ezopiclone tablets prescribed for each patient | numeric | 40 | 0 | 0% | 13176 | 100% | / |
| MSB_SUM | Sum of mood stabilizer orders prescribed for each patient | numeric | 66 | 0 | 0% | 13176 | 100% | / |
| BWSN | Number of orders of sodium valproate sustained-release tablets prescribed for each patient | numeric | 52 | 0 | 0% | 13176 | 100% | / |
| BWSN1 | Number of orders of sodium valproate tablets prescribed for each patient | numeric | 27 | 0 | 0% | 13176 | 100% | / |
| JBPD | Number of orders of gabapentin capsules prescribed for each patient | numeric | 12 | 0 | 0% | 13176 | 100% | / |
| KMXP | Number of orders of carbamazepine tablets prescribed for each patient | numeric | 11 | 0 | 0% | 13176 | 100% | / |
| LMSZ | Number of orders of lamotrigine tablets prescribed for each patient | numeric | 48 | 0 | 0% | 13176 | 100% | / |
| TBZ | Number of orders of topiramate capsules prescribed for each patient | numeric | 38 | 0 | 0% | 13176 | 100% | / |
| TSZ | Number of orders of lithium carbonate tablets prescribed for each patient | numeric | 44 | 0 | 0% | 13176 | 100% | / |
| ZSYBWSN | Number of orders of sodium valproate for injection prescribed for each patient | numeric | 23 | 0 | 0% | 13176 | 100% | / |
| ASE_SUM | Sum of anti-side effects drugs orders prescribed for each patient | numeric | 118 | 0 | 0% | 13176 | 100% | / |
| ASPL | Number of orders of aspirin enteric-coated tablets prescribed for each patient | numeric | 53 | 0 | 0% | 13176 | 100% | / |
| ATFTTG | Number of orders of atorvastatin clacium tablets prescribed for each patient | numeric | 61 | 0 | 0% | 13176 | 100% | / |
| BHS | Number of orders of benhexol hydrochloride tablets prescribed for each patient | numeric | 56 | 0 | 0% | 13176 | 100% | / |
| BSKD | Number of orders of bisacodyl enteric-coated tablets prescribed for each patient | numeric | 52 | 0 | 0% | 13176 | 100% | / |
| JYEC | Number of orders of polyethylene glycol prescribed for each patient | numeric | 48 | 0 | 0% | 13176 | 100% | / |
| KSL | Number of orders of glycerine enema prescribed for each patient | numeric | 29 | 0 | 0% | 13176 | 100% | / |
| MR | Number of orders of maren maru prescribed for each patient | numeric | 35 | 0 | 0% | 13176 | 100% | / |
| MSBL | Number of orders of mosapride citrate tablets prescribed for each patient | numeric | 52 | 0 | 0% | 13176 | 100% | / |
| HYP_SUM | Sum of new hypnotic orders prescribed for each patient | numeric | 45 | 0 | 0% | 13176 | 100% | / |
| ZPKL | Number of orders of zopiclone tablets prescribed for each patient | numeric | 2 | 0 | 0% | 13176 | 100% | / |
| ZZT | Number of orders of zopiclone tartrate tablets prescribed for each patient | numeric | 45 | 0 | 0% | 13176 | 100% | / |
| OT_SUM | Sum of β receptor blocker orders prescribed for each patient | numeric | 76 | 0 | 0% | 13176 | 100% | / |
| BSLE | Number of orders of bisoprolol fumarate tablets prescribed for each patient | numeric | 26 | 0 | 0% | 13176 | 100% | / |
| MTLE | Number of orders of metoprolol succinate sustained-release tablets prescribed for each patient | numeric | 48 | 0 | 0% | 13176 | 100% | / |
| MTLE1 | Number of orders of metoprolol tartrate tablets prescribed for each patient | numeric | 51 | 0 | 0% | 13176 | 100% | / |
| PNLE | Number of orders of propranolol hydrochloride tablets prescribed for each patient | numeric | 67 | 0 | 0% | 13176 | 100% | / |
| T3_SUM | Sum of hormonal drugs orders prescribed for each patient | numeric | 51 | 0 | 0% | 13176 | 100% | / |
| ZJZXSN | Number of orders of levothyroxine sodium tablets prescribed for each patient | numeric | 51 | 0 | 0% | 13176 | 100% | / |
| CM_SUM | Sum of Chinese patent medicines orders prescribed for each patient | numeric | 63 | 0 | 0% | 13176 | 100% | / |
| GWS | Number of orders of oryzanol tablets prescribed for each patient | numeric | 63 | 0 | 0% | 13176 | 100% | / |
| PHY_SUM | Sum of physiotherapy orders prescribed for each patient | numeric | 207 | 0 | 0% | 13176 | 100% | / |
| MPBT | Number of orders of multi-parameter biofeedback therapy prescribed for each patient | numeric | 13 | 0 | 0% | 13176 | 100% | / |
| ME | Number of orders of modified electroconvulsive therapy prescribed for each patient | numeric | 62 | 0 | 0% | 13176 | 100% | / |
| TMS | Number of orders of transcranial magnetic stimulation therapy prescribed for each patient | numeric | 11 | 0 | 0% | 13176 | 100% | / |
| TRMS | Number of orders of transcranial repetitive magnetic stimulation therapy prescribed for each patient | numeric | 7 | 0 | 0% | 13176 | 100% | / |
| EEG | Number of orders of electroencephalographic (EEG) biofeedback therapy prescribed for each patient | numeric | 92 | 0 | 0% | 13176 | 100% | / |
| BT | Number of orders of biofeedback therapy prescribed for each patient | numeric | 10 | 0 | 0% | 13176 | 100% | / |
| PSY_SUM | Sum of psychotherapy orders prescribed for each patient | numeric | 36 | 0 | 0% | 13176 | 100% | / |
| PSY | Number of orders of psychotherapy prescribed for each patient | numeric | 36 | 0 | 0% | 13176 | 100% | / |
| PE_body.temperature | Physical examination: to record a patient's body temperature | numeric | 84 | 330 | 2.50% | 12846 | 97.50% | Filling with data extracted from patients' discharge summary and medical record summary in the EMR |
| PE_pulse | Physical examination: to record a patient's pulse | numeric | 128 | 330 | 2.50% | 12846 | 97.50% | Filling with data extracted from patients' discharge summary and medical record summary in the EMR |
| PE_breath | Physical examination: to record a patient's breath | numeric | 62 | 327 | 2.48% | 12849 | 97.52% | Filling with data extracted from patients' discharge summary and medical record summary in the EMR |
| PE_SBP | Physical examination: to record a patient's systolic blood pressure (SBP) | numeric | 151 | 340 | 2.58% | 12836 | 97.42% | Filling with data extracted from patients' discharge summary and medical record summary in the EMR |
| PE_DBP | Physical examination: to record a patient's diastolic blood pressure (DBP) | numeric | 117 | 346 | 2.63% | 12830 | 97.37% | Filling with data extracted from patients' discharge summary and medical record summary in the EMR |
| admission_age | Age at admisison | numeric | 89 | 0 | 0% | 13176 | 100% | / |
| los | Length of stay | numeric | 101 | 0 | 0% | 13176 | 100% | / |
| sym_final_score^[1]^ | Frequency score of all symptoms | numeric | 3370 | 0 | 0% | 13176 | 100% | / |

[1] The calculation process of the variable “sym_final_score” is as follows:

1. Step one: Define the symptom set.

According to the 8 variables including symptom_1, symptom_2, symptom_3, symptom_4, symptom_5, symptom_6, symptom_7, symptom_8 (Table S1), all symptoms of the study population were extracted and the types of all symptoms were counted (see Equation S1).

Equation S1: $S=\{s_{1},s_{2},\cdots,s_{i},\cdots,s_{N}\}$, where each item $s_{i}$ represents one symptom and $N$is the total number of the types of all symptoms.

1. Step two: Define the duration set of each symptom.

According to the 8 variables including duration_1, duration_2, duration_3, duration_4, duration_5, duration_6, duration_7, duration_8 (Table S1), all durations of each symptom were extracted and the types of all durations for each symptom were counted (see Equation S2).

Equation S2: $D=\left\{ D_{s_{1}},D_{s_{2}},\cdots,D_{s_{i}},\cdots,D_{s_{N}} \right\}$, where each item $D_{s_{i}}$ represents one symptom’s duration set. And

$D_{s_{i}}=\left\{ d_{s_{i}}^{1},d_{s_{i}}^{2},\cdots,d_{s_{i}}^{j},\cdots,d_{s_{i}}^{M_{s_{i}}} \right\},$ where $D_{s_{i}}$ represents the symptom $s_{i}'s$ duration set and each item $d_{s_{i}}^{j}$ in the set represents the symptom $s_{i}'s$ one possible duration. $M_{s_{i}}$ is the total number of the types of all durations of the symptom $s_{i}$.

1. Step three: Compute the probability for each combination of symptom and the correspond duration.

The probability of each symptom (Equation S3): $P_{s_{i}}={Num(s_{i})}/{\sum_{i=1}^{N} Num(s_{i})}$, where $Num(s_{i})$ represents the frequency of the symptom $s_{i}$ for the whole study population.

The probability of each duration for the symptom$s_{i}$ (Equation S4): $P_{d_{s_{i}}^{j}}={Num(d_{s_{i}}^{j})}/{\sum_{j=1}^{M_{s_{i}}} Num(d_{s_{i}}^{j})}$, where $Num(d_{s_{i}}^{j})$ represents the frequency of the duration $d_{s_{i}}^{j}$ for symptom $s_{i}$.

The probability for each combination of symptom and the correspond duration (Equation S5): $P_{s_{i}}\times P_{d_{s_{i}}^{j}}=\left[ {Num\left( s_{i} \right)}/{\sum_{i=1}^{N} Num\left( s_{i} \right)} \right]\times[{Num\left( d_{s_{i}}^{j} \right)}/{\sum_{j=1}^{M_{s_{i}}} Num\left( d_{s_{i}}^{j} \right)}]$.

1. Step four: Compute the variable “sym_final_score” for patient $l (Equation S6)$.

Assume that patient $l$ has $K$ symptoms, and $K\in\left[ 1,N \right].$

Equation S6:

${sym\_final\_score}_{l}=\sum_{k=1}^{K} {(P}_{s_{i}}\times P_{d_{s_{i}}^{j}})=\sum_{k=1}^{K} (\left[ {Num\left( s_{i} \right)}/{\sum_{i=1}^{N} Num\left( s_{i} \right)} \right]\times[{Num\left( d_{s_{i}}^{j} \right)}/{\sum_{j=1}^{M_{s_{i}}} Num\left( d_{s_{i}}^{j} \right)}])$.

From the aforementioned calculation process of the variable “sym_final_score”, it can be concluded that the low value of the variable indicates that the patient’s symptoms and durations are rare or unique.

Table S2 Drugs, physiotherapies, and psychotherapies used in the antidepressant therapy

| Type of the therapy | Name of the therapy |
| --- | --- |
| T3 (Hormonal drugs) | Levothyroxine sodium tablets (ZJZXSN) |
| CM (Chinese patent medicine) | Oryzanol tablets (GWS) |
| OT (β receptor blocker) | Bisoprolol fumarate tablets (BSLE) |
|  | Metoprolol succinate sustained-release tablets (MTLE) |
|  | Metoprolol tartrate tablets (MTLE) |
|  | Propranolol hydrochloride tablets (PNLE) |
| MSB (Mood stabilizer) | Sodium valproate sustained-release tablets (BWSN) |
|  | Sodium valproate tablets (BWSN1) |
|  | Sodium valproate for injection (ZSYBWSN) |
|  | Gabapentin Capsules (JBPD) |
|  | Carbamazepine tablets (KMXP) |
|  | Lamotrigine tablets (LMSZ) |
|  | Lithium carbonate tablets (TSZ) |
|  | Topiramate capsules (TBZ) |
| HYP (New hypnotics) | Zolpidem tartrate tablets (ZZT) |
|  | Zopiclone (ZPKL) |
| ASE (Anti-side effects drugs) | Atorvastatin calcium tablets (ATFTTG) |
|  | Aspirin enteric-coated tablets (ASPL) |
|  | Bisacodyl enteric-coated tablets (BSKD) |
|  | Benhexol Hydrochloride tablets (BHS) |
|  | Polyethylene glycol (JYEC) |
|  | Kaysero (KSL) |
|  | Mosapride citrate tablets (MSBL) |
|  | Maren maru (MR) |
| AP (Antipsychotics) | Olanzapine tablets (ADP) |
|  | Quetiapine fumarate tablets (FMSZLP) |
|  | Clozapine tablets (LDP) |
|  | Aripiprazole orally disintegrating tablets (ALPZKBP) |
|  | Aripiprazole tablets (ALPZ) |
|  | Amisulpride tablets (AHBL) |
|  | Haloperidol for injection (FPDCZSY) |
|  | Haloperidol tablets (FPDC) |
|  | Tiapride hydrochloride tablets (LBL) |
|  | Risperidone tablets (LPT) |
|  | Chlorpromazine hydrochloride tablets (LBQ) |
|  | Chlorpromazine hydrochloride for injection (LBQ) |
|  | Chlorpromazine (LBZ) |
|  | Paliperidone sustained-release tablets (PLPT) |
|  | Sulpiride tablets (SBL) |
|  | Sulpiride for injection (SBL) |
|  | Ziprasidone hydrochloride capsules (YSQLXT) |
| ADP (Antidepressants) | Doxepin hydrochloride tablets (DSP) |
|  | Clomipramine hydrochloride tablets (LMPM) |
|  | Venlafaxine hydrochloride capsules (WLFX) |
|  | Escitalopram oxalate tablets (ASXTPL) |
|  | Amitriptyline hydrochloride tablets (AMTL) |
|  | Duloxetine hydrochloride enteric-coated capsules (DLXT) |
|  | Fluoxetine hydrochloride dispersible tablets (FXT) |
|  | Fluvoxamine maleate tablets (FFSM) |
|  | Flupentixol and meritroxine tablets (FPSDMLQX) |
|  | Mirtazapine tablets (MDP) |
|  | Maprotiline hydrochloride tablets (MPTL) |
|  | Paroxetine hydrochloride tablets (PLXT) |
|  | Paroxetine hydrochloride enteric-coated sustained-release tablets (PLXTHSP) |
|  | Sertraline hydrochloride tablets (SQL) |
|  | St. John’s wort extract tablets (SYHCTQW) |
|  | Citalopram hydrobromide tablets (XTPL) |
|  | Venlafaxine hydrochloride sustained-release capsules (YSWLFX) |
|  | Trazodone hydrochloride tablets (YSQZT) |
|  | Imipramine hydrochloride tablets (BMQ) |
| AA (Anxiolytics) | Alprazolam tablets (APZL) |
|  | Estazolam tablets (ASZL) |
|  | Diazepam tablets (DXP) |
|  | Diazepam for injection (DXPZSY) |
|  | Buspirone hydrochloride tablets (DLHT) |
|  | Clonazepam tablets (LXXP) |
|  | Clonazepam for injection (LXXPZSY) |
|  | Lorazepam tablets (LLXZ) |
|  | Midazolam maleate tablets (MDZL) |
|  | Midazolam for injection (MDZLZSY) |
|  | Tandospirone citrate capsules (TDLTJL) |
|  | Tandospirone citrate tablets (TDLT) |
|  | Nitrazepam Tablets (XXP) |
|  | Ezopiclone (YZPKL) |
| Physiotherapy (PHY) | Multi-parameter biofeedback therapy |
|  | Biofeedback therapy |
|  | Modified electroconvulsive therapy (ECT) |
|  | Transcranial magnetic stimulation |
|  | Transcranial repetitive magnetic stimulation therapy |
|  | Electroencephalographic (EEG) biofeedback therapy |
| Psychotherapy (PSY) | Psychotherapy |

Table S3 Quality of the numeric features

|  | Min | Max | Mean | StDev | 25% quantile | 75% quantile | range | Mean-3*StDev | Mean+3*StDev |
| --- | --- | --- | --- | --- | --- | --- | --- | --- | --- |
| order_SUM | 0 | 16232 | 599.1938 | 21.06991 | 372 | 711 | 0.057871 | 535.9841 | 662.4036 |
| ADP_SUM | 0 | 247 | 20.83865 | 4.063376 | 10 | 28 | 0.057871 | 8.648519 | 33.02877 |
| AMTL | 0 | 72 | 0.324605 | 1.661361 | 0 | 0 | 0.057871 | -4.65948 | 5.308689 |
| ASXTPL | 0 | 49 | 1.222526 | 2.21849 | 0 | 0 | 0.057871 | -5.43294 | 7.877996 |
| BMQ. | 0 | 23 | 0.006148 | 0.559004 | 0 | 0 | 0.057871 | -1.67086 | 1.68316 |
| DLXT | 0 | 79 | 0.463191 | 1.8758 | 0 | 0 | 0.057871 | -5.16421 | 6.090591 |
| DSP | 0 | 47 | 0.011688 | 0.717422 | 0 | 0 | 0.057871 | -2.14058 | 2.163955 |
| FFSM | 0 | 74 | 0.128947 | 1.484117 | 0 | 0 | 0.057871 | -4.3234 | 4.581296 |
| FPSDMLQX | 0 | 27 | 0.007514 | 0.582061 | 0 | 0 | 0.057871 | -1.73867 | 1.753697 |
| FXT | 0 | 99 | 1.832271 | 2.556527 | 0 | 0 | 0.057871 | -5.83731 | 9.501851 |
| LMPM | 0 | 81 | 0.167198 | 1.523721 | 0 | 0 | 0.057871 | -4.40397 | 4.738361 |
| MDP | 0 | 39 | 0.054493 | 0.978194 | 0 | 0 | 0.057871 | -2.88009 | 2.989075 |
| MDPP | 0 | 1 | 0.000228 | 0.122834 | 0 | 0 | 0.057871 | -0.36827 | 0.368729 |
| MPTL | 0 | 38 | 0.009183 | 0.690893 | 0 | 0 | 0.057871 | -2.0635 | 2.081863 |
| PLXT | 0 | 105 | 4.395188 | 3.16342 | 0 | 0 | 0.057871 | -5.09507 | 13.88545 |
| PLXTHSP | 0 | 24 | 0.014572 | 0.719316 | 0 | 0 | 0.057871 | -2.14338 | 2.172521 |
| SQL | 0 | 183 | 5.392532 | 3.312155 | 0 | 4 | 0.057871 | -4.54393 | 15.329 |
| SYHCTQW | 0 | 2 | 0.000152 | 0.131999 | 0 | 0 | 0.057871 | -0.39584 | 0.396147 |
| WLFX | 0 | 125 | 0.560944 | 2.134978 | 0 | 0 | 0.057871 | -5.84399 | 6.965878 |
| XTPL | 0 | 55 | 0.295689 | 1.650738 | 0 | 0 | 0.057871 | -4.65652 | 5.247903 |
| YSDSPP | 0 | 64 | 0.2034 | 1.443323 | 0 | 0 | 0.057871 | -4.12657 | 4.533371 |
| YSQZT | 0 | 27 | 0.014117 | 0.657708 | 0 | 0 | 0.057871 | -1.95901 | 1.98724 |
| YSWLFX | 0 | 151 | 5.734062 | 3.431343 | 0 | 3 | 0.057871 | -4.55997 | 16.02809 |
| AP_SUM | 0 | 333 | 13.75934 | 4.166593 | 0 | 22 | 0.057871 | 1.259556 | 26.25911 |
| ADP | 0 | 140 | 6.503415 | 3.346175 | 0 | 12 | 0.057871 | -3.53511 | 16.54194 |
| AHBL | 0 | 52 | 0.06269 | 1.19202 | 0 | 0 | 0.057871 | -3.51337 | 3.638751 |
| ALPZ | 0 | 87 | 0.060033 | 1.191497 | 0 | 0 | 0.057871 | -3.51446 | 3.634523 |
| ALPZKBP | 0 | 45 | 0.283015 | 1.580658 | 0 | 0 | 0.057871 | -4.45896 | 5.024989 |
| FMSZLP | 0 | 98 | 3.019733 | 2.830748 | 0 | 0 | 0.057871 | -5.47251 | 11.51198 |
| FPDC | 0 | 54 | 0.014876 | 0.834361 | 0 | 0 | 0.057871 | -2.48821 | 2.517959 |
| FPDCZSY | 0 | 29 | 0.041135 | 0.732342 | 0 | 0 | 0.057871 | -2.15589 | 2.23816 |
| LBL | 0 | 50 | 0.414921 | 1.700562 | 0 | 0 | 0.057871 | -4.68677 | 5.516609 |
| LBQ | 0 | 38 | 0.007514 | 0.679351 | 0 | 0 | 0.057871 | -2.03054 | 2.045566 |
| LBQ | 0 | 7 | 0.001746 | 0.298757 | 0 | 0 | 0.057871 | -0.89453 | 0.898017 |
| LBZ | 0 | 7 | 0.000835 | 0.254176 | 0 | 0 | 0.057871 | -0.76169 | 0.763363 |
| LDP | 0 | 238 | 0.647237 | 2.285083 | 0 | 0 | 0.057871 | -6.20801 | 7.502486 |
| LPT | 0 | 75 | 0.375607 | 1.901691 | 0 | 0 | 0.057871 | -5.32947 | 6.080681 |
| PLPT | 0 | 68 | 0.092213 | 1.249586 | 0 | 0 | 0.057871 | -3.65655 | 3.840972 |
| SBL | 0 | 99 | 0.050243 | 1.194238 | 0 | 0 | 0.057871 | -3.53247 | 3.632957 |
| SBL1 | 0 | 115 | 0.103901 | 1.337661 | 0 | 0 | 0.057871 | -3.90908 | 4.116884 |
| SBLP | 0 | 50 | 0.108834 | 1.264347 | 0 | 0 | 0.057871 | -3.68421 | 3.901877 |
| SBLZSY | 0 | 33 | 0.079007 | 1.060185 | 0 | 0 | 0.057871 | -3.10155 | 3.259563 |
| YSQLXT | 0 | 23 | 0.005616 | 0.528979 | 0 | 0 | 0.057871 | -1.58132 | 1.592554 |
| ZLP | 0 | 107 | 1.886764 | 2.533815 | 0 | 0 | 0.057871 | -5.71468 | 9.488209 |
| AA_SUM | 0 | 377 | 25.59472 | 4.883808 | 7 | 38 | 0.057871 | 10.94329 | 40.24614 |
| APZL | 0 | 255 | 11.54394 | 4.10021 | 0 | 19 | 0.057871 | -0.75669 | 23.84457 |
| ASZL | 0 | 181 | 0.306542 | 1.752472 | 0 | 0 | 0.057871 | -4.95088 | 5.563959 |
| DLHT | 0 | 54 | 0.115361 | 1.262265 | 0 | 0 | 0.057871 | -3.67143 | 3.902156 |
| DXP | 0 | 144 | 0.333333 | 1.772508 | 0 | 0 | 0.057871 | -4.98419 | 5.650858 |
| DXPZSJ | 0 | 1 | 0.000379 | 0.139561 | 0 | 0 | 0.057871 | -0.4183 | 0.419062 |
| DXPZSY | 0 | 18 | 0.050395 | 0.679005 | 0 | 0 | 0.057871 | -1.98662 | 2.087408 |
| LLXZ | 0 | 94 | 2.371812 | 2.803233 | 0 | 0 | 0.057871 | -6.03789 | 10.78151 |
| LXXP | 0 | 171 | 9.27292 | 3.986417 | 0 | 16 | 0.057871 | -2.68633 | 21.23217 |
| LXXPZSY | 0 | 26 | 0.082878 | 0.898313 | 0 | 0 | 0.057871 | -2.61206 | 2.777818 |
| mdzl | 0 | 42 | 0.072025 | 1.103026 | 0 | 0 | 0.057871 | -3.23705 | 3.381103 |
| MDZLZSY | 0 | 38 | 0.070583 | 0.717328 | 0 | 0 | 0.057871 | -2.0814 | 2.222566 |
| TDLT | 0 | 11 | 0.003264 | 0.423764 | 0 | 0 | 0.057871 | -1.26803 | 1.274555 |
| TDLTJL | 0 | 49 | 0.904599 | 2.087072 | 0 | 0 | 0.057871 | -5.35662 | 7.165815 |
| XXP | 0 | 33 | 0.009487 | 0.685533 | 0 | 0 | 0.057871 | -2.04711 | 2.066087 |
| YZPKL | 0 | 48 | 0.457195 | 1.710693 | 0 | 0 | 0.057871 | -4.67489 | 5.589275 |
| MSB_SUM | 0 | 108 | 1.730267 | 2.542639 | 0 | 0 | 0.057871 | -5.89765 | 9.358184 |
| BWSN | 0 | 108 | 0.795841 | 2.065443 | 0 | 0 | 0.057871 | -5.40049 | 6.99217 |
| BWSN1 | 0 | 39 | 0.059882 | 1.073115 | 0 | 0 | 0.057871 | -3.15946 | 3.279228 |
| JBPD | 0 | 13 | 0.009032 | 0.523026 | 0 | 0 | 0.057871 | -1.56005 | 1.57811 |
| KMXP | 0 | 25 | 0.012447 | 0.648901 | 0 | 0 | 0.057871 | -1.93426 | 1.959151 |
| LMSZ | 0 | 76 | 0.301988 | 1.68004 | 0 | 0 | 0.057871 | -4.73813 | 5.34211 |
| TBZ | 0 | 75 | 0.147996 | 1.383074 | 0 | 0 | 0.057871 | -4.00122 | 4.297217 |
| TSZ | 0 | 52 | 0.339633 | 1.644575 | 0 | 0 | 0.057871 | -4.59409 | 5.273357 |
| ZSYBWSN | 0 | 37 | 0.063449 | 0.938135 | 0 | 0 | 0.057871 | -2.75096 | 2.877852 |
| ASE_SUM | 0 | 441 | 5.873406 | 3.72713 | 0 | 7 | 0.057871 | -5.30798 | 17.0548 |
| ASPL | 0 | 141 | 0.795993 | 2.065583 | 0 | 0 | 0.057871 | -5.40076 | 6.992741 |
| ATFTTG | 0 | 176 | 0.84745 | 2.151892 | 0 | 0 | 0.057871 | -5.60823 | 7.303127 |
| BHS | 0 | 109 | 0.828552 | 2.124747 | 0 | 0 | 0.057871 | -5.54569 | 7.202792 |
| BSKD | 0 | 95 | 1.265255 | 2.127358 | 0 | 0 | 0.057871 | -5.11682 | 7.647328 |
| JYEC | 0 | 123 | 0.935868 | 2.008838 | 0 | 0 | 0.057871 | -5.09065 | 6.962382 |
| KSL | 0 | 265 | 0.236111 | 1.724195 | 0 | 0 | 0.057871 | -4.93647 | 5.408697 |
| MR | 0 | 80 | 0.206815 | 1.370937 | 0 | 0 | 0.057871 | -3.906 | 4.319627 |
| MSBL | 0 | 74 | 0.756982 | 2.000967 | 0 | 0 | 0.057871 | -5.24592 | 6.759883 |
| HYP_SUM | 0 | 57 | 0.607772 | 1.839797 | 0 | 0 | 0.057871 | -4.91162 | 6.127162 |
| ZPKL | 0 | 3 | 0.000228 | 0.161665 | 0 | 0 | 0.057871 | -0.48477 | 0.485221 |
| ZZT | 0 | 57 | 0.607544 | 1.839781 | 0 | 0 | 0.057871 | -4.9118 | 6.126886 |
| OT_SUM | 0 | 186 | 3.149135 | 2.959617 | 0 | 0 | 0.057871 | -5.72972 | 12.02799 |
| BSLE | 0 | 81 | 0.060109 | 1.127531 | 0 | 0 | 0.057871 | -3.32249 | 3.442704 |
| MTLE | 0 | 92 | 0.436248 | 1.778564 | 0 | 0 | 0.057871 | -4.89945 | 5.771941 |
| MTLE1 | 0 | 186 | 0.266469 | 1.893441 | 0 | 0 | 0.057871 | -5.41385 | 5.946793 |
| PNLE | 0 | 74 | 2.386308 | 2.711741 | 0 | 0 | 0.057871 | -5.74891 | 10.52153 |
| T3_SUM | 0 | 126 | 0.437234 | 1.863355 | 0 | 0 | 0.057871 | -5.15283 | 6.027299 |
| ZJZXSN | 0 | 126 | 0.437234 | 1.863355 | 0 | 0 | 0.057871 | -5.15283 | 6.027299 |
| CM_SUM | 0 | 80 | 2.086445 | 2.612835 | 0 | 0 | 0.057871 | -5.75206 | 9.92495 |
| GWS | 0 | 80 | 2.086445 | 2.612835 | 0 | 0 | 0.057871 | -5.75206 | 9.92495 |
| PHY_SUM | 0 | 349 | 17.6593 | 5.689409 | 0 | 28 | 0.057871 | 0.591077 | 34.72753 |
| MPBT | 0 | 56 | 0.382514 | 1.504732 | 0 | 0 | 0.057871 | -4.13168 | 4.896711 |
| ME | 0 | 315 | 5.62075 | 5.141136 | 0 | 0 | 0.057871 | -9.80266 | 21.04416 |
| TMS | 0 | 18 | 0.085231 | 0.712074 | 0 | 0 | 0.057871 | -2.05099 | 2.221454 |
| TRMS | 0 | 15 | 0.006603 | 0.418055 | 0 | 0 | 0.057871 | -1.24756 | 1.260769 |
| EEG | 0 | 349 | 11.50607 | 4.249828 | 0 | 24 | 0.057871 | -1.24341 | 24.25555 |
| BT | 0 | 40 | 0.053734 | 0.967207 | 0 | 0 | 0.057871 | -2.84789 | 2.955354 |
| PSY_SUM | 0 | 44 | 2.630768 | 2.027999 | 0 | 4 | 0.057871 | -3.45323 | 8.714766 |
| PSY | 0 | 44 | 2.630768 | 2.027999 | 0 | 4 | 0.057871 | -3.45323 | 8.714766 |
| PE_body.temperature | 1 | 84 | 39.4896 | 3.087915 | 37 | 45 | 0.057871 | 30.22586 | 48.75335 |
| PE_pulse | 1 | 128 | 85.97344 | 5.712587 | 80 | 106 | 0.057871 | 68.83567 | 103.1112 |
| PE_breath | 1 | 62 | 20.79652 | 2.101655 | 22 | 22 | 0.057871 | 14.49156 | 27.10149 |
| PE_SBP | 1 | 151 | 40.02376 | 5.623522 | 21 | 48 | 0.057871 | 23.15319 | 56.89432 |
| PE_DBP | 1 | 117 | 81.34821 | 4.812873 | 73 | 96 | 0.057871 | 66.90959 | 95.78683 |
| admission_age | 8 | 97 | 45.52846 | 4.368359 | 29 | 61 | 0.057871 | 32.42338 | 58.63354 |
| los | 1 | 220 | 16.43412 | 3.205931 | 10 | 21 | 0.057871 | 6.816331 | 26.05191 |
| sym_final_score | 0 | 0.160106 | 0.02848 | 0.182318 | 0.001153 | 0.059024 | 0.057871 | -0.51848 | 0.575435 |

Table S4 Quality of the factor features

| **Variable name** | **Level** | **Count** |
| --- | --- | --- |
| gender | :male:female | :4411:8765 |
| age_group | :>=60:0-17:18-35:36-59 | :3516:1163:3064:5433 |
| marital_status | :missing:single:divorced:other:widowed:unmarried:married | :45:8:552:35:545:3027:8964 |
| job | :missing:unknown:self-employed:worker:civil servant:teacher:soldier:farmer:other:retired:unemployed:student:doctor:staff:technician: freelancer | :275:45:263:570:1082:4:27:1750:2331:1774:1036:1982:1:977:667:392 |
| nationality | :missing:Buyi:Tibetan:Korean:Daur:Dong:Kazakh:Han:Li:Lisu:Other:She:Tujia:Tu:Wa:Uyghur: Yao: Gelao | :13:7:731:1:1:5:2:12129:1:1:252:1:20:2:1:2:1:6 |
| pat_type | :faculty of the hospital:retired officials:gold card for non-members: gold card for members:medical insurance:own expenses | :18:22:52:36:18:4:13026 |
| pay_type | :provincial medical insurance:city medical insurance:cash | :346:4770:8060 |
| pat_source | :missing:Chengdu:foreign:other cities in Sichuan:other provinces | :397:6698:10:4357:1714 |
| hometown | :Anhui:Beijing:Unknown:Fujian:Gansu:Guangdong:Guangxi:Guizhou:Hainan:Hebei:Henan:Heilongjiang:Hubei:Hunan:Jilin:Jiangsu:Jiangxi:Liaoning:Inner Mongolia:Ningxia:Qinghai:Shandong:Shanxi:Shaanxi:Shanghai:Sichuan:Taiwan:Tianjin:missing: Tibet:Hong Kong:Xinjiang:Yunnan:Zhejiang:Chongqing | :26:15:71:25:170:23:12:207:2:47:65:27:53:33:19:47:25:34:8:24:74:71:42:57:15:10666:1:8:2:565:2:69:172:68:431 |
| in_year | :2010:2011:2012:2013:2014:2015:2016:2017:2018 | :80:1555:1404:1347:1473:1536:1663:2060:2058 |
| seasonality | :spring:winter:autumn:summer | :3420:3037:3407:3312 |
| out_diagnosis_code | :0:1:2:3:4:5:6:7:8:9:10:11:12 | :888:271:432:205:588:175:190:64:113:41:414:9713:82 |
| out_diagnosis_NO | :1:2:3:4:5:6:7 | :4808:2807:1700:1138:835:616:1272 |
| out_diagnosis_MDDIndex | :1:2:3:4:5:6:7 | :9713:1415:697:514:348:287:202 |
| severity | :other:mild:unspecified:moderate:severe | :21:20:9864:309:2962 |
| first_episode | :first episode:recurrent episode | :11864:1312 |
| cur_dep | :ICU:Infectious Diseases one:Biliary Surgery:Pediatric Geriatrics:Otorhinolaryngology Head and Neck Surgery:Lung Cancer Center:Rheumatology:Hepatobiliary and Pancreatic Surgery:Hepatovascular Surgery: Infectious Diseases two:Cadre Medical-Geriatrics:Orthopedics:Nuclear Medicine:Respiratory Medicine:Respiratory And Critical Care Medicine:Thyroid and Breast Surgery:Tuberculosis:Jinka Medical Unit:Psychiatry:Rehabilitation Medicine:Aesthetic Plastic/Burn Surgery:Urology:Endocrinology:Dermatology:General Internal Medicine:Neurology:Neurology Surgery:Nephrology:Special Needs Medical Center:Pain:Head and Neck Oncology:Gastrointestinal Surgery:Gastroenterology:Mental Health Center:Psychological Comprehensive Medical Unit:Psychosomatic Disorder Medical Unit:Cardiology:Cardiac Surgery:Thoracic Oncology:Thoracic Surgery:Department of Hematology:Ophthalmology:Pancreatic Surgery:Department of Integrated Traditional Chinese and Western Medicine:Department of Oncology One :Department of Oncology Two:Department of Oncology:Department of Oncology Three | :9:32:24:2649:27:1:71:11:29:33:308:96:3:60:19:42:16:145:1152:78:2:37:112:51:3:1184:55:44:8:73:2:38:54:7:2991:3309:154:9:1:38:23:38:9:55:22:21:31 |
| if_trans | :no:yes | :12746:430 |
| in_rank | :1:2:3:4:5:6:7:8:9:10:11:12:13:14:16:17:18:20:23 | :11724:984:261:94:47:13:12:13:5:6:2:2:3:2:2:1:3:1:1 |
| main_MDD_readmission | :0:1 | :12450:726 |
| main_psychiatry_readmission | :0:1 | :12347:829 |
| MDD_readmission | :0:1 | :11724:1452 |
| psychiatry_readmission | :0:1 | :11724:1452 |
| cancer_comorbidity_NO | :0:1:2:3:4:5 | :13000:108:43:18:5:2 |
| respiratory_comorbidity_NO | :0:1:2:3:4:5 | :11987:968:191:25:4:1 |
| circulatory_comorbidity_NO | :0:1:2:3:4:5:6 | :10346:1923:630:209:58:9:1 |
| digestive_comorbidity_NO | :0:1:2:3:4:5:6 | :11141:1558:372:80:21:3:1 |
| nervous_comorbidity_NO | :0:1:2:3:4 | :12183:865:108:19:1 |
| endocrine_comorbidity_NO | :0:1:2:3:4:5 | :10815:1911:378:61:10:1 |
| psychiatric_comorbidity_NO | :0:1:2:3 | :8361:4347:442:26 |
| comorbidity | :0:1 | :4808:8368 |
| psychiatric_comorbidity | :0:1 | :8361:4815 |
| endocrine_comorbidity | :0:1 | :10815:2361 |
| nervous_comorbidity | :0:1 | :12183:993 |
| digestive_comorbidity | :0:1 | :11141:2035 |
| circulatory_comorbidity | :0:1 | :10346:2830 |
| respiratory_comorbidity | :0:1 | :11987:1189 |
| cancer_comorbidity | :0:1 | :13000:176 |
| surgery_NO | :0:1:2:3:4:5 | :11892:1094:120:28:6:36 |
| operation_NO | :0:1:2:3:4:5 | :11892:678:294:106:71:135 |
| history_allergy | :no:yes | :11341:1835 |
| history_blood_transfusion | :no:yes | :12692:484 |
| history_drug_use | :frequently:occasionally:no | :1986:641:10549 |
| history_surgery | :no:yes | :8259:4917 |
| history_smoking | :no:yes | :12500:676 |
| history_alcoholism | :no:yes | :12663:513 |
| mood | :0:1 | :5129:8047 |
| bad_sleep | :0:1 | :8532:4644 |
| loss_interest | :0:1 | :11772:1404 |
| flustered | :0:1 | :11759:1417 |
| worry | :0:1 | :12404:772 |
| tension | :0:1 | :12465:711 |
| upset | :0:1 | :12251:925 |
| headache | :0:1 | :12551:625 |
| dizziness | :0:1 | :12272:904 |
| physical_discomfort | :0:1 | :12383:793 |
| fatigue | :0:1 | :12598:578 |
| suicide | :0:1 | :12652:524 |
| self_harm | :0:1 | :13044:132 |
| hallucination | :0:1 | :12931:245 |
| less_activity | :0:1 | :12727:449 |
| chest_tightness | :0:1 | :12815:361 |
| afraid | :0:1 | :12994:182 |
| irritability | :0:1 | :13008:168 |
| fidget | :0:1 | :13017:159 |
| slow_response | :0:1 | :13032:144 |
| relapse | :0:1 | :10927:2249 |
| symp_worsen | :0:1 | :8208:4968 |
| core_symp | :0:1:2 | :4999:6903:1274 |
| psy_symp | :0:1:2:3:4 | :9667:2670:779:57:3 |
| phy_symp | :0:1:2:3:4:5 | :6252:4927:1632:330:34:1 |
| combo_sym | :0:1:2:3:4:5:7:8:9:10:11:12:13:14:15:16:17:18 | :2035:8047:802:65:195:67:75:289:335:88:188:36:44:112:90:86:160:462 |
| PE_expression | :missing:indifferent:dreadful:painful:exciting:anxious:natural | :353:185:1:123:3:243:12268 |
| PE_face | :missing:acute:chronic:other:normal | :353:280:1011:53:11479 |
| PE_nutrition | :missing:malnourished:cachectic:good:medium | :327:224:9:11894:722 |
| PE_cooperation | :missing:no:yes | :353:251:12572 |
| PE_consciousness | :missing:stupor:coma:confused:aware:somnolence:delirium | :353:6:6:23:12761:23:4 |
| PE_gait | :missing:abnormal:normal | :356:512:12308 |
| PE_body.position | :missing:passive:other:coercive:natural | :354:164:29:22:12607 |
| ADP_type | :0:1:2:3:4:6 | :1648:9379:1892:232:23:2 |
| AP_type | :0:1:2:3:4:5:6:7 | :5220:5586:1817:434:100:14:3:2 |
| AA_type | :0:1:2:3:4:5:6:7 | :2001:5582:3711:1463:356:51:10:2 |
| MSB_type | :0:1:2:3:4 | :11860:1168:143:4:1 |
| ASE_type | :0:1:2:3:4:5:6:7 | :8422:2768:1345:467:152:17:4:1 |
| HYP_type | :0:1 | :12440:736 |
| OT_type | :0:1:2 | :10764:2323:89 |
| T3_type | :0:1 | :12810:366 |
| CM_type | :0:1 | :11346:1830 |
| PHY_type | :0:1:2:3:4 | :7240:4944:835:146:11 |
| PSY_type | :0:1 | :6422:6754 |
| main_drug | :0:1:2:3:4:5:6:7:8:9 | :935:769:4185:303:4093:1106:1029:203:407:146 |

Since some features (such as out_diag_code, out_diag_name, out_diag_code1, out_diag_name1, out_diag_code2, out_diag_name2, out_diag_code3, out_diag_name3, out_diag_code4, out_diag_name4, out_diag_code5, out_diag_name5, out_diag_code6 and out_diag_name6, chief_complaint, symptom_1, duration_1, symptom_2, duration_2, symptom_3, duration_3, symptom_4, duration_4, symptom_5, duration_5, symptom_6, duration_6, symptom_7, duration_7, symptom_8, duration_8 and PE_subcutaneous.bleeding) have too many levels, it is difficult to present them in Table S3.

Based on the above results, we preprocessed the raw data, including outliers processing of the numeric features, classification and integration of raw levels of the factor features, and initial feature filtering. Finally, 165 features were kept and used in the analyses.

- 1. **Segmentation of training and testing datasets**

To assess the prediction performance of ML models on a different dataset and to ensure the unbiased approximation of the model’s generalizability to new patients, each prediction-modeling cohort was split into multiple training and testing datasets according to the period obtained of the data respectively (details in the Table S5). We segmented the training set and testing set according to patients’ discharge date.

**1.5.1 30-day cohort**

(1) The initial train-test split

Since cases in the prediction-modeling cohort of 30-day psychiatric readmission referred to patients whose discharge date of the index admission located between January 2009 and November 2018, patients whose discharge date of the index admission is before January 1, 2018 were classified as the training set (11026 patients, 84.97%), and patients whose discharge date of the index admission is during 2018 were classified as the testing set (1950 patients, 15.03%). We used the 11026 patients’ records (non-readmission ratio=10656/11026=96.64%; readmission ratio=370/11026=3.36%) in the training set to establish models and predicted the 30-day psychiatric readmission risk of the 1950 patients (non-readmission ratio=1889/1950=96.87%; readmission ratio=61/1950=3.13%) in the testing set.

To avoid favorable train-test splits in the data, the 30-day prediction-modeling cohort was split into other three train-test splits.

(2) The second train-test split

Patients whose discharge date of the index admission is during 2017 were classified as the testing set (2023 patients, 15.59%), and the other patients were classified as the training set (10953 patients, 84.41%). We used the 10953 patients’ records (non-readmission ratio=10592/10953=96.70%; readmission ratio=361/10953=3.30%) in the training set to establish models and predicted the 30-day psychiatric readmission risk of the 2023 patients (non-readmission ratio=1953/2023=96.54%; readmission ratio=70/2023=3.46%) in the testing set.

(3) The third train-test split

Patients whose discharge date of the index admission is during 2016 were classified as the testing set (1682 patients, 12.96%), and the other patients were classified as the training set (11294 patients, 87.04%). We used the 11294 patients’ records (non-readmission ratio=10932/11294=96.79%; readmission ratio=362/11294=3.21%) in the training set to establish models and predicted the 30-day psychiatric readmission risk of the 1682 patients (non-readmission ratio=1613/1682=95.90%; readmission ratio=69/1682=4.10%) in the testing set.

(4) The fourth train-test split

Patients whose discharge date of the index admission is during 2015 were classified as the testing set (1514 patients, 11.67%), and the other patients were classified as the training set (11462 patients, 88.33%). We used the 11462 patients’ records (non-readmission ratio=11074/11462=96.61%; readmission ratio=388/11462=3.39%) in the training set to establish models and predicted the 30-day psychiatric readmission risk of the 1514 patients (non-readmission ratio=1471/1514=97.16%; readmission ratio=43/1514=2.84%) in the testing set.

**1.5.2 60-day cohort**

(1) The initial train-test split

Since cases in the prediction-modeling cohort of 60-day psychiatric readmission referred to patients whose discharge date of the index admission located between January 2009 and October 2018, patients whose discharge date of the index admission is before January 1, 2018 were classified as the training set (11026 patients, 86.24%), and patients whose discharge date of the index admission is during 2018 were classified as the testing set (1759 patients, 13.76%). We used the 11026 patients’ records (non-readmission ratio=10528/11026=95.48%; readmission ratio=498/11026=4.52%) in the training set to establish models and predicted the 60-day psychiatric readmission risk of the 1759 patients (non-readmission ratio=1684/1759=95.74%; readmission ratio=75/1759=4.26%) in the testing set.

To avoid favorable train-test splits in the data, the 60-day prediction-modeling cohort was split into other three train-test splits.

(2) The second train-test split

Patients whose discharge date of the index admission is during 2017 were classified as the testing set (2023 patients, 15.82%), and the other patients were classified as the training set (10762 patients, 84.18%). We used the 10762 patients’ records (non-readmission ratio=10273/10762=95.46%; readmission ratio=489/10762=4.54%) in the training set to establish models and predicted the 60-day psychiatric readmission risk of the 2023 patients (non-readmission ratio=1939/2023=95.85%; readmission ratio=84/2023=4.15%) in the testing set.

(3) The third train-test split

Patients whose discharge date of the index admission is during 2016 were classified as the testing set (1682 patients, 13.16%), and the other patients were classified as the training set (11103 patients, 86.84%). We used the 11103 patients’ records (non-readmission ratio=10619/11103=95.64%; readmission ratio=484/11103=4.36%) in the training set to establish models and predicted the 60-day psychiatric readmission risk of the 1682 patients (non-readmission ratio=1593/1682=94.71%; readmission ratio=89/1682=5.29%) in the testing set.

(4) The fourth train-test split

Patients whose discharge date of the index admission is during 2015 were classified as the testing set (1514 patients, 11.84%), and the other patients were classified as the training set (11271 patients, 88.16%). We used the 11271 patients’ records (non-readmission ratio=10758/11271=95.45%; readmission ratio=513/11271=4.55%) in the training set to establish models and predicted the 60-day psychiatric readmission risk of the 1514 patients (non-readmission ratio=1454/1514=96.04%; readmission ratio=60/1514=3.96%) in the testing set.

**1.5.3 90-day cohort**

(1) The initial train-test split

Since cases in the prediction-modeling cohort of 90-day psychiatric readmission referred to patients whose discharge date of the index admission located between January 2009 and September 2018, patients whose discharge date of the index admission is before January 1, 2018 were classified as the training set (11026 patients, 87.36%), and patients whose discharge date of the index admission is during 2018 were classified as the testing set (1596 patients, 12.64%). We used the 11026 patients’ records (non-readmission ratio=10433/11026=94.62%; readmission ratio=593/11026=5.38%) in the training set to establish models and predicted the 90-day psychiatric readmission risk of the 1596 patients (non-readmission ratio=1511/1596=94.67%; readmission ratio=85/1596=5.33%) in the testing set.

To avoid favorable train-test splits in the data, the 90-day prediction-modeling cohort was split into other three train-test splits.

(2) The second train-test split

Patients whose discharge date of the index admission is during 2017 were classified as the testing set (2023 patients, 16.03%), and the other patients were classified as the training set (10599 patients, 83.97%). We used the 10599 patients’ records (non-readmission ratio=10018/10599=94.52%; readmission ratio=581/10599=5.48%) in the training set to establish models and predicted the 90-day psychiatric readmission risk of the 2023 patients (non-readmission ratio=1926/2023=95.21%; readmission ratio=97/2023=4.79%) in the testing set.

(3) The third train-test split

Patients whose discharge date of the index admission is during 2016 were classified as the testing set (1682 patients, 13.33%), and the other patients were classified as the training set (10940 patients, 86.67%). We used the 10940 patients’ records (non-readmission ratio=10364/10940=94.73%; readmission ratio=576/10940=5.27%) in the training set to establish models and predicted the 90-day psychiatric readmission risk of the 1682 patients (non-readmission ratio=1580/1682=93.94%; readmission ratio=102/1682=6.06%) in the testing set.

(4) The fourth train-test split

Patients whose discharge date of the index admission is during 2015 were classified as the testing set (1514 patients, 11.99%), and the other patients were classified as the training set (11108 patients, 88.01%). We used the 11108 patients’ records (non-readmission ratio=10507/11108=94.59%; readmission ratio=601/11108=5.41%) in the training set to establish models and predicted the 90-day psychiatric readmission risk of the 1514 patients (non-readmission ratio=1437/1514=94.91%; readmission ratio=77/1514=5.09%) in the testing set.

**1.5.4 180-day cohort**

(1) The initial train-test split

Since cases in the prediction-modeling cohort of 180-day psychiatric readmission referred to patients whose discharge date of the index admission located between January 2009 and June 2018, patients whose discharge date of the index admission is before January 1, 2018 were classified as the training set (11026 patients, 92.02%), and patients whose discharge date of the index admission is during 2018 were classified as the testing set (956 patients, 7.98%). We used the 11026 patients’ records (non-readmission ratio=10184/11026=92.36%; readmission ratio=842/11026=7.64%) in the training set to establish models and predicted the 180-day psychiatric readmission risk of the 956 patients (non-readmission ratio=881/956=92.15%; readmission ratio=75/956=7.85%) in the testing set.

To avoid favorable train-test splits in the data, the 180-day prediction-modeling cohort was split into other three train-test splits.

(2) The second train-test split

Patients whose discharge date of the index admission is during 2017 were classified as the testing set (2023 patients, 16.88%), and the other patients were classified as the training set (9959 patients, 83.12%). We used the 9959 patients’ records (non-readmission ratio=9184/9959=92.22%; readmission ratio=775/9959=7.78%) in the training set to establish models and predicted the 180-day psychiatric readmission risk of the 2023 patients (non-readmission ratio=1881/2023=92.98%; readmission ratio=142/2023=7.02%) in the testing set.

(3) The third train-test split

Patients whose discharge date of the index admission is during 2016 were classified as the testing set (1682 patients, 14.04%), and the other patients were classified as the training set (10300 patients, 85.96%). We used the 10300 patients’ records (non-readmission ratio=9519/10300=92.42%; readmission ratio=781/10300=7.58%) in the training set to establish models and predicted the 180-day psychiatric readmission risk of the 1682 patients (non-readmission ratio=1546/1682=91.91%; readmission ratio=136/1682=8.09%) in the testing set.

(4) The fourth train-test split

Patients whose discharge date of the index admission is during 2015 were classified as the testing set (1514 patients, 12.64%), and the other patients were classified as the training set (10468 patients, 87.36%). We used the 10468 patients’ records (non-readmission ratio=9670/10468=92.38%; readmission ratio=798/10468=7.62%) in the training set to establish models and predicted the 180-day psychiatric readmission risk of the 1514 patients (non-readmission ratio=1395/1514=92.14%; readmission ratio=119/1514=7.86%) in the testing set.

**1.5.4 365-day cohort**

(1) The initial train-test split

Since cases in the prediction-modeling cohort of 365-day psychiatric readmission referred to patients whose discharge date of the index admission located between January 2009 and December 2017, patients whose discharge date of the index admission is before January 1, 2017 were classified as the training set (9003 patients, 81.65%), and patients whose discharge date of the index admission is during 2017 were classified as the testing set (2023 patients, 18.35%). We used the 9003 cases (non-readmission ratio=7978/9003=88.61%; readmission ratio=1025/9003=11.39%) in the training set to establish models and predicted the 365-day psychiatric readmission risk of the 2023 cases (non-readmission ratio=1827/2023=90.31%; readmission ratio=196/2023=9.69%) in the testing set.

To avoid favorable train-test splits in the data, the 365-day prediction-modeling cohort was split into other three train-test splits.

(2) The second train-test split

Patients whose discharge date of the index admission is during 2016 were classified as the testing set (1682 patients, 15.25%), and the other patients were classified as the training set (9344 patients, 84.75%). We used the 9344 patients’ records (non-readmission ratio=8323/9344=89.07%; readmission ratio=1021/9344=10.93%) in the training set to establish models and predicted the 365-day psychiatric readmission risk of the 1682 patients (non-readmission ratio=1482/1682=88.11%; readmission ratio=200/1682=11.89%) in the testing set.

(3) The third train-test split

Patients whose discharge date of the index admission is during 2015 were classified as the testing set (1514 patients, 13.73%), and the other patients were classified as the training set (9512 patients, 86.27%). We used the 9512 patients’ records (non-readmission ratio=8461/9512=88.95%; readmission ratio=1051/9512=11.05%) in the training set to establish models and predicted the 365-day psychiatric readmission risk of the 1514 patients (non-readmission ratio=1344/1514=88.77%; readmission ratio=170/1514=11.23%) in the testing set.

(4) The fourth train-test split

Patients whose discharge date of the index admission is during 2014 were classified as the testing set (1484 patients, 13.46%), and the other patients were classified as the training set (9542 patients, 86.54%). We used the 9542 patients’ records (non-readmission ratio=8487/9542=88.94%; readmission ratio=1055/9542=11.06%) in the training set to establish models and predicted the 365-day psychiatric readmission risk of the 1484 patients (non-readmission ratio=1318/1484=88.81%; readmission ratio=166/1484=11.19%) in the testing set.

According to the above results of segmentation of training and testing datasets for each cohort, the non-readmission ratio (or the readmission ratio) in the training set is almost the same as the non-readmission ratio (or the readmission ratio) in the testing set.

Table S5 Multiple different train-test splits and test samples distributed in time compared to training sets

| Follow-up | Train-test splits | Year of discharge of patients in the testing dataset | No. and percentage of patients in the training dataset  (non-readmission ratio & readmission ratio) | No. and percentage of patients in the testing dataset  (non-readmission ratio & readmission ratio) |
| --- | --- | --- | --- | --- |
| 30-day | The initial split | During 2018 | 11026 patients, 84.97%  (96.64% & 3.36%) | 1950 patients, 15.03%  (96.87% & 3.13%) |
|  | The second split | During 2017 | 10953 patients, 84.41%  (96.70% & 3.30%) | 2023 patients, 15.59%  (96.54% & 3.46%) |
|  | The third split | During 2016 | 11294 patients, 87.04%  (96.79% & 3.21%) | 1682 patients, 12.96%  (95.90% & 4.10%) |
|  | The fourth split | During 2015 | 11462 patients, 88.33%  (96.61% & 3.39%) | 1514 patients, 11.67%  (97.16% & 2.84%) |
| 60-day | The initial split | During 2018 | 11026 patients, 86.24%  (95.48% & 4.52%) | 1759 patients, 13.76%  (95.74% & 4.26%) |
|  | The second split | During 2017 | 10762 patients, 84.18%  (95.46% & 4.54%) | 2023 patients, 15.82%  (95.85% & 4.15%) |
|  | The third split | During 2016 | 11103 patients, 86.84%  (95.64% & 4.36%) | 1682 patients, 13.16%  (94.71% & 5.29%) |
|  | The fourth split | During 2015 | 11271 patients, 88.16%  (95.45% & 4.55%) | 1514 patients, 11.84%  (96.04% & 3.96%) |
| 90-day | The initial split | During 2018 | 11026 patients, 87.36%  (94.62% & 5.38%) | 1596 patients, 12.64%  (94.67% & 5.33%) |
|  | The second split | During 2017 | 10599 patients, 83.97%  (94.52% & 5.48%) | 2023 patients, 16.03%  (95.21% & 4.79%) |
|  | The third split | During 2016 | 10940 patients, 86.67%  (94.73% & 5.27%) | 1682 patients, 13.33%  (93.94% & 6.06%) |
|  | The fourth split | During 2015 | 11108 patients, 88.01%  (94.59% & 5.41%) | 1514 patients, 11.99%  (94.91% & 5.09%) |
| 180-day | The initial split | During 2018 | 11026 patients, 92.02%  (92.36% & 7.64%) | 956 patients, 7.98%  (92.15% & 7.85%) |
|  | The second split | During 2017 | 9959 patients, 83.12%  (92.22% & 7.78%) | 2023 patients, 16.88%  (92.98% & 7.02%) |
|  | The third split | During 2016 | 10300 patients, 85.96%  (92.42% & 7.58%) | 1682 patients, 14.04%  (91.91% & 8.09%) |
|  | The fourth split | During 2015 | 10468 patients, 87.36%  (92.38% & 7.62%) | 1514 patients, 12.64%  (92.14% & 7.86%) |
| 365-day | The initial split | During 2017 | 9003 patients, 81.65%  (88.61% & 11.39%) | 2023 patients, 18.35%  (90.31% & 9.69%) |
|  | The second split | During 2016 | 9344 patients, 84.75%  (89.07% & 10.93%) | 1682 patients, 15.25%  (88.11% & 11.89%) |
|  | The third split | During 2015 | 9512 patients, 86.27%  (88.95% & 11.05%) | 1514 patients, 13.73%  (88.77% & 11.23%) |
|  | The fourth split | During 2014 | 9542 patients, 86.54%  (88.94% & 11.06%) | 1484 patients, 13.46%  (88.81% & 11.19%) |

- 1. **Machine learning pipeline architecture**

Within our pipeline we conducted feature selection and hyperparameter optimization.

**1.6.1 Predictor selection**

For predictor selection, we used the statistically hypothesis testing using only training dataset, particularly, two-sided t test for continuous variables and chi-squared test for categorical variables were used. It allows the dissection of patients’ heterogeneity in all recruited predictors, and it has the benefits of both partly minimization of overfitting and variable selection. Variables with p value < 0.05 were considered as significant pridictors and were entered in the ML models.

**1.6.2 Parameter tuning**

We conducted an exhaustive grid search to tune the hyperparameters of four algorithms. A grid search allows us to search all specified hyperparameters (parameters not directly learnt within a model) that maximize our cross-validation score according to a specified criterion. In our case, the criterion was the maximization of area under the curve on the receiver operator characteristic.

For the SVM, the hyperparameters were type = “C-classification”, kernel = “radial”, gamma = 10^(-6:0), cost = 10^(-4:2). For the XGB, the hyperparameters were nrounds = c(seq(100,500,100)), max_depth = c(2:6), eta = seq(0,1,0.05), gamma = seq(0,0.1,0.05), colsample_bytree= seq(0.25,0.75,0.1), subsample = seq(0.5,1,0.1), min_child_weight = c(0). For the RF, the hyperparameters were ntree = seq(200,1500,5), mtry = seq(2,10,1).

**(1) 30-day cohort**

(a) The initial train-test split

For the SVM, the final hyperparameters were cost = 1, gamma = 0.00001, kernel = radial, scale = TRUE, probability = TRUE). For the XGB, the final hyperparameters were nrounds = 100, max_depth = 3, eta = 0.1, gamma = 0.05, colsample_bytree= 0.75, subsample = 0.5, min_child_weight = 0, method = “xgbTree”, tuneLength = 10. For the RF, the final hyperparameters were ntree = 900, mtry = 2, importance = TRUE.

(b) The second train-test split

For the SVM, the final hyperparameters were cost = 0.01, gamma = 0.00001, kernel = radial, scale = TRUE, probability = TRUE). For the XGB, the final hyperparameters were nrounds = 100, max_depth = 3, eta = 0.1, gamma = 0.05, colsample_bytree= 0.75, subsample = 1, min_child_weight = 0, method = “xgbTree”, tuneLength = 10. For the RF, the final hyperparameters were ntree = 510, mtry = 3, importance = TRUE.

(c) The third train-test split

For the SVM, the final hyperparameters were cost = 10, gamma = 0.0001, kernel = radial, scale = TRUE, probability = TRUE). For the XGB, the final hyperparameters were nrounds = 100, max_depth = 3, eta = 0.1, gamma = 0.05, colsample_bytree= 0.75, subsample = 1, min_child_weight = 0, method = “xgbTree”, tuneLength = 10. For the RF, the final hyperparameters were ntree = 920, mtry = 3, importance = TRUE.

(d) The fourth train-test split

For the SVM, the final hyperparameters were cost = 100, gamma = 0.00001, kernel = radial, scale = TRUE, probability = TRUE). For the XGB, the final hyperparameters were nrounds = 100, max_depth = 3, eta = 0.1, gamma = 0.05, colsample_bytree= 0.85, subsample = 1, min_child_weight = 0, method = “xgbTree”, tuneLength = 10. For the RF, the final hyperparameters were ntree = 500, mtry = 4, importance = TRUE.

**(2) 60-day cohort**

(a) The initial train-test split

For the SVM, the final hyperparameters were cost = 1, gamma = 0.000001, kernel = radial, scale = TRUE, probability = TRUE). For the XGB, the final hyperparameters were nrounds = 100, max_depth = 2, eta = 0.1, gamma = 0, colsample_bytree= 0.35, subsample = 1, min_child_weight = 0, method = “xgbTree”, tuneLength = 10. For the RF, the final hyperparameters were ntree = 600, mtry = 3, importance = TRUE.

(b) The second train-test split

For the SVM, the final hyperparameters were cost = 1, gamma = 0.01, kernel = radial, scale = TRUE, probability = TRUE). For the XGB, the final hyperparameters were nrounds = 100, max_depth = 3, eta = 0.1, gamma = 0.05, colsample_bytree= 0.85, subsample = 1, min_child_weight = 0, method = “xgbTree”, tuneLength = 10. For the RF, the final hyperparameters were ntree = 190, mtry = 4, importance = TRUE.

(c) The third train-test split

For the SVM, the final hyperparameters were cost = 1, gamma = 0.01, kernel = radial, scale = TRUE, probability = TRUE). For the XGB, the final hyperparameters were nrounds = 100, max_depth = 3, eta = 0.1, gamma = 0.05, colsample_bytree= 0.55, subsample = 1, min_child_weight = 0, method = “xgbTree”, tuneLength = 10. For the RF, the final hyperparameters were ntree = 850, mtry = 5, importance = TRUE.

(d) The fourth train-test split

For the SVM, the final hyperparameters were cost = 1, gamma = 0.001, kernel = radial, scale = TRUE, probability = TRUE). For the XGB, the final hyperparameters were nrounds = 100, max_depth = 3, eta = 0.1, gamma = 0.05, colsample_bytree= 0.25, subsample = 1, min_child_weight = 0, method = “xgbTree”, tuneLength = 10. For the RF, the final hyperparameters were ntree = 250, mtry = 4, importance = TRUE.

**(3) 90-day cohort**

(a) The initial train-test split

For the SVM, the final hyperparameters were cost = 0.01, gamma = 0.001, kernel = radial, scale = TRUE, probability = TRUE). For the XGB, the final hyperparameters were nrounds = 200, max_depth = 2, eta = 0.1, gamma = 0.05, colsample_bytree= 0.45, subsample = 1, min_child_weight = 0, method = “xgbTree”, tuneLength = 10. For the RF, the final hyperparameters were ntree = 550, mtry = 5, importance = TRUE.

(b) The second train-test split

For the SVM, the final hyperparameters were cost = 5, gamma = 0.001, kernel = radial, scale = TRUE, probability = TRUE). For the XGB, the final hyperparameters were nrounds = 100, max_depth = 3, eta = 0.1, gamma = 0.05, colsample_bytree= 0.45, subsample = 1, min_child_weight = 0, method = “xgbTree”, tuneLength = 10. For the RF, the final hyperparameters were ntree = 300, mtry = 5, importance = TRUE.

(c) The third train-test split

For the SVM, the final hyperparameters were cost = 0.5, gamma = 0.01, kernel = radial, scale = TRUE, probability = TRUE). For the XGB, the final hyperparameters were nrounds = 100, max_depth = 3, eta = 0.1, gamma = 0.05, colsample_bytree= 0.25, subsample = 1, min_child_weight = 0, method = “xgbTree”, tuneLength = 10. For the RF, the final hyperparameters were ntree = 200, mtry = 6, importance = TRUE.

(d) The fourth train-test split

For the SVM, the final hyperparameters were cost = 1, gamma = 0.001, kernel = radial, scale = TRUE, probability = TRUE). For the XGB, the final hyperparameters were nrounds = 100, max_depth = 2, eta = 0.1, gamma = 0.05, colsample_bytree= 0.85, subsample = 1, min_child_weight = 0, method = “xgbTree”, tuneLength = 10. For the RF, the final hyperparameters were ntree = 200, mtry = 4, importance = TRUE.

**(4) 180-day cohort**

(a) The initial train-test split

For the SVM, the final hyperparameters were cost = 1, gamma = 0.00001, kernel = radial, scale = TRUE, probability = TRUE). For the XGB, the final hyperparameters were nrounds = 100, max_depth = 3, eta = 0.1, gamma = 0.05, colsample_bytree= 0.55, subsample = 1, min_child_weight = 0, method = “xgbTree”, tuneLength = 10. For the RF, the final hyperparameters were ntree = 1290, mtry = 2, importance = TRUE.

(b) The second train-test split

For the SVM, the final hyperparameters were cost = 0.1, gamma = 0.0001, kernel = radial, scale = TRUE, probability = TRUE). For the XGB, the final hyperparameters were nrounds = 100, max_depth = 2, eta = 0.1, gamma = 0.05, colsample_bytree= 0.25, subsample = 1, min_child_weight = 0, method = “xgbTree”, tuneLength = 10. For the RF, the final hyperparameters were ntree = 295, mtry = 4, importance = TRUE.

(c) The third train-test split

For the SVM, the final hyperparameters were cost = 10, gamma = 0.0001, kernel = radial, scale = TRUE, probability = TRUE). For the XGB, the final hyperparameters were nrounds = 100, max_depth = 3, eta = 0.1, gamma = 0.05, colsample_bytree= 0.65, subsample = 1, min_child_weight = 0, method = “xgbTree”, tuneLength = 10. For the RF, the final hyperparameters were ntree = 205, mtry = 4, importance = TRUE.

(d) The fourth train-test split

For the SVM, the final hyperparameters were cost = 1, gamma = 0.001, kernel = radial, scale = TRUE, probability = TRUE). For the XGB, the final hyperparameters were nrounds = 100, max_depth = 2, eta = 0.1, gamma = 0.05, colsample_bytree= 0.55, subsample = 1, min_child_weight = 0, method = “xgbTree”, tuneLength = 10. For the RF, the final hyperparameters were ntree = 890, mtry = 2, importance = TRUE.

**(5) 365-day cohort**

(a) The initial train-test split

For the SVM, the final hyperparameters were cost = 10, gamma = 0.0001, kernel = radial, scale = TRUE, probability = TRUE). For the XGB, the final hyperparameters were nrounds = 100, max_depth = 3, eta = 0.1, gamma = 0.05, colsample_bytree= 0.25, subsample = 1, min_child_weight = 0, method = “xgbTree”, tuneLength = 10. For the RF, the final hyperparameters were ntree = 1290, mtry = 3, importance = TRUE.

(b) The second train-test split

For the SVM, the final hyperparameters were cost = 100, gamma = 0.0001, kernel = radial, scale = TRUE, probability = TRUE). For the XGB, the final hyperparameters were nrounds = 100, max_depth = 2, eta = 0.1, gamma = 0.05, colsample_bytree= 0.95, subsample = 1, min_child_weight = 0, method = “xgbTree”, tuneLength = 10. For the RF, the final hyperparameters were ntree = 1300, mtry = 5, importance = TRUE.

(c) The third train-test split

For the SVM, the final hyperparameters were cost = 0.5, gamma = 0.001, kernel = radial, scale = TRUE, probability = TRUE). For the XGB, the final hyperparameters were nrounds = 100, max_depth = 2, eta = 0.1, gamma = 0.05, colsample_bytree= 0.7, subsample = 1, min_child_weight = 0, method = “xgbTree”, tuneLength = 10. For the RF, the final hyperparameters were ntree = 790, mtry = 2, importance = TRUE.

(d) The fourth train-test split

For the SVM, the final hyperparameters were cost = 10, gamma = 0.001, kernel = radial, scale = TRUE, probability = TRUE). For the XGB, the final hyperparameters were nrounds = 100, max_depth = 2, eta = 0.1, gamma = 0.05, colsample_bytree= 0.55, subsample = 1, min_child_weight = 0, method = “xgbTree”, tuneLength = 10. For the RF, the final hyperparameters were ntree = 925, mtry = 2, importance = TRUE.

**1.6.3 Cross Validation**

Cross-validation was conducted using 5 repeats of 10-fold cross-validation (method = “repeatedcv” in the trainControl function). Research suggests that 10-fold cross-validation may better balance the bias-variance trade-off be compared to the more expensive leave-one-out cross-validation. In addition, repeated runs are suggested to avoid favorable splits in the data that may lead to overly optimistic accuracy estimates.

**Supplementary Information SI2:**

**2.1 Heterogeneity between readmitted patients and non-readmitted patients**

**2.1.1 Sociodemographic and clinical features**

We identified 12976 unique patients with the discharge diagnosis of MDD in the 30-day cohort, with 431 (3.32%) of those patients readmitted with a psychiatric diagnosis within 30 days post-discharge. The median (IQR) age of the study population was 46 ([30, 61]) years, and 66.5% of patients were female. The median (IQR) of length of stay was 15 ([10, 21]) days. Patients who were readmitted with a psychiatric diagnosis within 30 days were more likely to be female, younger-aged (under 17 years old) student, older-aged (over 60 years old) retired, unmarried or widowed, and to have medical insurances.

Patients in the readmission group vs. patients in the non-readmission group had longer length of stay (median, 20 [range, 11-27] days vs. 15 [range, 10-21] days; p < 0.001), and were more likely to be admitted in winter and treated in the pediatrics, geriatrics, and psychiatric care unit. Compared with the non-readmitted patients, readmitted patients’ index admission was more likely to be a readmission with a diagnosis of MDD, indicating the history of prior non-MDD admissions. Readmitted patients were more likely to have less diagnoses (comorbidities) and to be diagnosed as severely recurrent MDD, whilst they were less likely to have surgeries during the index admission.

**2.1.2 Symptoms**

When considering each individual symptom, readmitted patients were more likely to have symptoms like mood-down, flustered, dizziness, physical discomfort, afraid, and presented recurrence of these symptoms. According to the Guidelines for prevention and treatment of MDD in China, the manifestations of depressive episodes can be divided into three categories: core symptom group, psychological symptom group and physical symptom group. Overall, 63% of the study population had at least one core symptom. The proportions of patients with at least one psychological symptom and at least one physical symptom were 27% and 53.4%, respectively. Based on each patient’s chief complaint, the number of symptoms occurred in each group were counted. A significantly higher proportion of readmitted patients had a greater number of core symptoms and psychological symptoms than that of the patients who were not readmitted, indicating that patients with more core symptoms and psychological symptoms were more likely to be readmitted. Meanwhile, the combination of key symptoms of each patient was analyzed and summarized. Readmitted patients were more likely to mainly be mood-down, accompanied by other symptoms (Table 1). Particularly, symptom combinations were sorted and selected according to the proportion of more than 0.5% of readmitted patients who have the specific combination (Table 2). For readmitted patients, the most frequent combinations were mood-down and worsen of the symptom (6.5%), mood-down (5.6%), mood-down, bad sleep and worsen of the symptoms (5.3%), and mood-down, bad sleep and presented recurrence of these symptoms (4.6%). Readmitted patients were more likely to have combinations of recurrent mood-down, suicide ideation, psychotic symptoms, while non-readmitted patients were more likely to have combinations of mood-down and physical symptoms (bad sleep, dizziness, fatigue, etc.).

**2.1.3 Prior illness history, vital signs, and comorbidity**

The results show that more non-readmitted patients have a history of smoking and alcoholism. Non-readmitted patients were more likely to have sickly look and uncooperative with the physical examination, while readmitted patients were more likely to have abnormal gait. Moreover, readmitted patients showed higher pulse than non-readmitted patients. Patients who didn’t have diagnoses indicating comorbid medical illnesses were more likely to be in the group with psychiatric readmission, possibly reflecting that most of the readmitted patients only had a principal diagnosis of MDD without any other comorbid illnesses.

**2.1.4 Treatment patterns**

By analyzing doctors’ order dataset, the antidepressant drugs, physiotherapies, and psychotherapies used in the treatment of the study population were extracted. The total number of medical orders for readmitted patients was significantly larger than the number for non-readmitted patients. Specifically, the sum of orders of antidepressants (ADP), antipsychotics (AP), anxiolytics (AA), mood stabilizers (MSB), anti-side effects drugs (ASE), β receptor blockers (OT), hormonal drugs (T3), Chinese patent medicines (CM), physiotherapy (PHY) and psychotherapy (PSY), the number of orders of fluoxetine hydrochloride dispersible tablets, venlafaxine hydrochloride sustained-release capsules, olanzapine tablets, quetiapine fumarate tablets, clozapine tablets, sulpiride tablets, alprazolam tablets, lorazepam tablets, clonazepam tablets, midazolam for injection, tandospirone citrate capsules, ezopiclone tablets, lamotrigine tablets, lithium carbonate tablets, aspirin enteric-coated tablets, atorvastatin calcium tablets, benhexol hydrochloride tablets, bisacodyl enteric-coated tablets, zopiclone tartrate tablets, propranolol hydrochloride tablets, modified electroconvulsive therapy, and electroencephalographic (EEG) biofeedback therapy of readmitted patients were significantly different from that of the non-readmitted patients.

We analyzed the type of drugs in each cluster (ADP, AP, AA, MSB, ASE, new hypnotics (HYP), OT, T3, and CM), the type of physiotherapies and psychotherapies used for the treatment of each patient. The results showed that readmitted patients were more likely to have a greater number of drug types of ADP, AP, AA, ASE, HYP, OT, and CM than the patients who were not readmitted, indicating that patients who were treated with more drug types were more likely to be readmitted. Similarly, patients who were treated with more physiotherapy and psychotherapy types were more likely to be readmitted.

The combination of all medication, physiotherapy, and psychotherapy types that were utilized for the treatment of each patient during the inpatient psychiatric service period was summarized in Table 3. All combinations were sorted and selected according to the proportion of more than 0.5% of readmitted patients who have the specific combination. Since the study population included the patients who were treated in the orthopedics, urology, and other non-mental health care units and who also had a co-diagnosis of MDD, they might not undergo antidepressant therapy during the index admission. In patients who received antidepressant treatment, the most frequent used treatment pattern was the combination of ADP, AP, AA, PHY, and PSY (7.3%), followed by the combination of ADP, AP, AA, and PSY (4.6%), the combination of ADP and AA (4.5%), and the combination of ADP, AP, AA, ASE, PHY, and PSY (4.0%). Moreover, the combination of ADP, AP, and AA, the combination of ADP, AP, AA, ASE, PHY, and PSY, the combination of ADP, AP, AA, and PHY, the combination of ADP, AP, AA, ASE, and PSY, the combination of ADP, AP, AA, ASE, and PHY, the combination of ADP, AP, AA, and ASE, were utilized more often to patients who were readmitted with a psychiatric diagnosis, where all these combinations included ADP, AP, and AA. However, the combinations including ADP and AA were utilized more often to patients who were not readmitted with a psychiatric diagnosis.

See Table 1 and Table S6 for the comparison results of the readmission population (case) and the non-readmission population (control) regarding all included features.

Table S6: Characteristics of clinical, comorbidity, prior history, vital signs, symptoms, and treatment-related of the 30-day cohort.

| **Variable** | **Variable name** | **All** | **Non-readmission** | **Readmission** | **P-value** |
| --- | --- | --- | --- | --- | --- |
|  |  | **(n = 12976)** | **(n = 12545)** | **(n = 431)** |  |
| ***Clinical variables*** | | |  |  |  |
| **Year at admission (%)** | **in_year** |  |  |  | **0.578** |
| 2010 |  | 80 (0.6) | 78 (0.6) | 2 (0.5) |  |
| 2011 |  | 1555 (12.0) | 1507 (12.0) | 48 (11.1) |  |
| 2012 |  | 1404 (10.8) | 1361 (10.8) | 43 (10.0) |  |
| 2013 |  | 1347 (10.4) | 1292 (10.3) | 55 (12.8) |  |
| 2014 |  | 1473 (11.4) | 1431 (11.4) | 42 (9.7) |  |
| 2015 |  | 1536 (11.8) | 1489 (11.9) | 47 (10.9) |  |
| 2016 |  | 1663 (12.8) | 1598 (12.7) | 65 (15.1) |  |
| 2017 |  | 2060 (15.9) | 1989 (15.9) | 71 (16.5) |  |
| 2018 |  | 1858 (14.3) | 1800 (14.3) | 58 (13.5) |  |
| **Seasonality at admission (%)** | **seasonality** |  |  |  | **<0.001** |
| Spring |  | 3420 (26.4) | 3311 (26.4) | 109 (25.3) |  |
| Summer |  | 3312 (25.5) | 3209 (25.6) | 103 (23.9) |  |
| Autumn |  | 3323 (25.6) | 3237 (25.8) | 86 (20.0) |  |
| Winter |  | 2921 (22.5) | 2788 (22.2) | 133 (30.9) |  |
| **Specialty care unit (%)** | **cur_dep** |  |  |  | **<0.001** |
| Other |  | 1865 (14.4) | 1851 (14.8) | 14 (3.2) |  |
| Pediatrics and Geriatrics |  | 2607 (20.1) | 2440 (19.4) | 167 (38.7) |  |
| Neurology |  | 1158 (8.9) | 1151 (9.2) | 7 (1.6) |  |
| Psychosomatic disorders |  | 3278 (25.3) | 3170 (25.3) | 108 (25.1) |  |
| Psychology |  | 2950 (22.7) | 2861 (22.8) | 89 (20.6) |  |
| Mental Disorders |  | 1118 (8.6) | 1072 (8.5) | 46 (10.7) |  |
| **Whether a patient transferred to other units (%)** | **if_trans** |  |  |  | **1** |
| No |  | 12554 (96.7) | 12137 (96.7) | 417 (96.8) |  |
| Yes |  | 422 (3.3) | 408 (3.3) | 14 (3.2) |  |
| **Whether the index admission is a readmission with a principal diagnosis of MDD (%)** | **main_MDD_readmission** |  |  |  | **<0.001** |
| No |  | 12259 (94.5) | 11880 (94.7) | 379 (87.9) |  |
| Yes |  | 717 (5.5) | 665 (5.3) | 52 (12.1) |  |
| **Whether the index admission is a readmission with a principal or supplementary diagnosis of MDD (%)** | **MDD_readmission** |  |  |  | **0.042** |
| No |  | 11546 (89.0) | 11176 (89.1) | 370 (85.8) |  |
| Yes |  | 1430 (11.0) | 1369 (10.9) | 61 (14.2) |  |
| **Principal diagnosis code (%)** | **out_diagnosis_code** |  |  |  | **<0.001** |
| Other |  | 874 (6.7) | 866 (6.9) | 8 (1.9) |  |
| ICD-10 code M00-M99: Diseases of the musculoskeletal system |  | 269 (2.1) | 267 (2.1) | 2 (0.5) |  |
| ICD-10 code I00-I99: Diseases of the circulatory system |  | 429 (3.3) | 425 (3.4) | 4 (0.9) |  |
| ICD-10 code J00-J99: Diseases of the respiratory system |  | 205 (1.6) | 205 (1.6) | 0 (0.0) |  |
| ICD-10 code G00-G99: Diseases of the nervous system |  | 570 (4.4) | 568 (4.5) | 2 (0.5) |  |
| ICD-10 code C00-C99: Cancer |  | 174 (1.3) | 174 (1.4) | 0 (0.0) |  |
| ICD-10 code G00-G99: Diseases of the digestive system |  | 188 (1.4) | 187 (1.5) | 1 (0.2) |  |
| ICD-10 code F00-F09: Organic (including symptomatic) mental disorders |  | 62 (0.5) | 60 (0.5) | 2 (0.5) |  |
| ICD-10 code F10-F19: Mental and behavioral disorders caused using psychoactive substances |  | 111 (0.9) | 106 (0.8) | 5 (1.2) |  |
| ICD-10 code F20-F29: Schizophrenia, schizophrenia, and delusional mental disorders |  | 40 (0.3) | 40 (0.3) | 0 (0.0) |  |
| ICD-10 code F40-F48: Neurotic, stressful and somatoform disorders |  | 409 (3.2) | 399 (3.2) | 10 (2.3) |  |
| ICD-10 code F32-F33: Depression |  | 9563 (73.7) | 9168 (73.1) | 395 (91.6) |  |
| Other Mental and behavioral disorders |  | 82 (0.6) | 80 (0.6) | 2 (0.5) |  |
| **Number of all diagnoses (%)** | **out_diagnosis_NO** |  |  |  | **0.024** |
| One diagnosis |  | 4734 (36.5) | 4555 (36.3) | 179 (41.5) |  |
| Two diagnoses |  | 2767 (21.3) | 2665 (21.2) | 102 (23.7) |  |
| Three diagnoses |  | 1683 (13.0) | 1624 (12.9) | 59 (13.7) |  |
| Four diagnoses |  | 1128 (8.7) | 1100 (8.8) | 28 (6.5) |  |
| Five diagnoses |  | 819 (6.3) | 800 (6.4) | 19 (4.4) |  |
| Six diagnoses |  | 601 (4.6) | 587 (4.7) | 14 (3.2) |  |
| Seven diagnoses |  | 1244 (9.6) | 1214 (9.7) | 30 (7.0) |  |
| **Location of MDD diagnosis in all diagnoses (%)** | **out_diagnosis_MDDIndex** |  |  |  | **<0.001** |
| Principal diagnosis is MDD |  | 9563 (73.7) | 9168 (73.1) | 395 (91.6) |  |
| Supplementary diagnosis one is MDD |  | 1397 (10.8) | 1374 (11.0) | 23 (5.3) |  |
| Supplementary diagnosis two is MDD |  | 687 (5.3) | 681 (5.4) | 6 (1.4) |  |
| Supplementary diagnosis three is MDD |  | 506 (3.9) | 501 (4.0) | 5 (1.2) |  |
| Supplementary diagnosis four is MDD |  | 341 (2.6) | 341 (2.7) | 0 (0.0) |  |
| Supplementary diagnosis five is MDD |  | 284 (2.2) | 283 (2.3) | 1 (0.2) |  |
| Supplementary diagnosis six is MDD |  | 198 (1.5) | 197 (1.6) | 1 (0.2) |  |
| **Severity of MDD (%)** | **severity** |  |  |  | **0.001** |
| Other |  | 17 (0.1) | 17 (0.1) | 0 (0.0) |  |
| Unspecified |  | 9767 (75.3) | 9476 (75.5) | 291 (67.5) |  |
| Mild |  | 18 (0.1) | 18 (0.1) | 0 (0.0) |  |
| Moderate |  | 277 (2.1) | 270 (2.2) | 7 (1.6) |  |
| Severe |  | 2897 (22.3) | 2764 (22.0) | 133 (30.9) |  |
| **Whether the MDD diagnosis is a first episode or a recurrent episode (%)** | **first_episode** |  |  |  | **0.007** |
| First episode |  | 11684 (90.0) | 11313 (90.2) | 371 (86.1) |  |
| Recurrent episode |  | 1292 (10.0) | 1232 (9.8) | 60 (13.9) |  |
| **Number of surgeries during this admission (%)** | **surgery_NO** |  |  |  | **<0.001** |
| Without surgeries during this admission |  | 11719 (90.3) | 11304 (90.1) | 415 (96.3) |  |
| With one surgery during this admission |  | 1071 (8.3) | 1060 (8.4) | 11 (2.6) |  |
| With two surgeries during this admission |  | 119 (0.9) | 118 (0.9) | 1 (0.2) |  |
| With three surgeries during this admission |  | 26 (0.2) | 26 (0.2) | 0 (0.0) |  |
| With four surgeries during this admission |  | 6 (0.0) | 6 (0.0) | 0 (0.0) |  |
| With five surgeries during this admission |  | 35 (0.3) | 31 (0.2) | 4 (0.9) |  |
| **Number of operations during this admission (%)** | **operation_NO** |  |  |  | **0.001** |
| Without operations during this admission |  | 11719 (90.3) | 11304 (90.1) | 415 (96.3) |  |
| With one operation during this admission |  | 660 (5.1) | 651 (5.2) | 9 (2.1) |  |
| With two operations during this admission |  | 291 (2.2) | 291 (2.3) | 0 (0.0) |  |
| With three operations during this admission |  | 104 (0.8) | 102 (0.8) | 2 (0.5) |  |
| With four operations during this admission |  | 69 (0.5) | 68 (0.5) | 1 (0.2) |  |
| With five operations during this admission |  | 133 (1.0) | 129 (1.0) | 4 (0.9) |  |
| **Length of stay (los) (median [IQR])** | **los** | 15.00 [10.00, 21.00] | 15.00 [10.00, 21.00] | 20.00 [11.00, 27.00] | **<0.001** |
| ***Comorbidity variables*** | | |  |  |  |
| **Whether the patient has a diagnosis of a medical comorbidity (%)** | **comorbidity** |  |  |  | **0.031** |
| No |  | 4734 (36.5) | 4555 (36.3) | 179 (41.5) |  |
| Yes |  | 8242 (63.5) | 7990 (63.7) | 252 (58.5) |  |
| **Whether the patient has a comorbidity of cancer (%)** | **cancer_comorbidity** |  |  |  | **0.326** |
| No |  | 12801 (98.7) | 12373 (98.6) | 428 (99.3) |  |
| Yes |  | 175 (1.3) | 172 (1.4) | 3 (0.7) |  |
| **Whether the patient has a comorbidity of respiratory diseases (%)** | **respiratory_comorbidity** |  |  |  | **0.208** |
| No |  | 11806 (91.0) | 11406 (90.9) | 400 (92.8) |  |
| Yes |  | 1170 (9.0) | 1139 (9.1) | 31 (7.2) |  |
| **Whether the patient has a comorbidity of circulatory diseases (%)** | **circulatory_comorbidity** |  |  |  | **0.109** |
| No |  | 10178 (78.4) | 9826 (78.3) | 352 (81.7) |  |
| Yes |  | 2798 (21.6) | 2719 (21.7) | 79 (18.3) |  |
| **Whether the patient has a comorbidity of digestive diseases (%)** | **digestive_comorbidity** |  |  |  | **0.082** |
| No |  | 10980 (84.6) | 10602 (84.5) | 378 (87.7) |  |
| Yes |  | 1996 (15.4) | 1943 (15.5) | 53 (12.3) |  |
| **Whether the patient has a comorbidity of nervous diseases (%)** | **nervous_comorbidity** |  |  |  | **1** |
| No |  | 12003 (92.5) | 11604 (92.5) | 399 (92.6) |  |
| Yes |  | 973 (7.5) | 941 (7.5) | 32 (7.4) |  |
| **Whether the patient has a comorbidity of endocrine diseases (%)** | **endocrine_comorbidity** |  |  |  | **0.982** |
| No |  | 10648 (82.1) | 10295 (82.1) | 353 (81.9) |  |
| Yes |  | 2328 (17.9) | 2250 (17.9) | 78 (18.1) |  |
| **Whether the patient has a comorbidity of psychiatric diseases (%)** | **psychiatric_comorbidity** |  |  |  | **<0.001** |
| No |  | 8232 (63.4) | 7896 (62.9) | 336 (78.0) |  |
| Yes |  | 4744 (36.6) | 4649 (37.1) | 95 (22.0) |  |
| **Number of cancer comorbidity (%)** | **cancer_comorbidity_NO** |  |  |  | **0.698** |
| Without cancer comorbidity |  | 12801 (98.7) | 12373 (98.6) | 428 (99.3) |  |
| With one cancer comorbidity |  | 108 (0.8) | 107 (0.9) | 1 (0.2) |  |
| With two cancer comorbidities |  | 43 (0.3) | 41 (0.3) | 2 (0.5) |  |
| With three cancer comorbidities |  | 17 (0.1) | 17 (0.1) | 0 (0.0) |  |
| With four cancer comorbidities |  | 5 (0.0) | 5 (0.0) | 0 (0.0) |  |
| With five cancer comorbidities |  | 2 (0.0) | 2 (0.0) | 0 (0.0) |  |
| **Number of respiratory comorbidity (%)** | **respiratory_comorbidity_NO** |  |  |  | **0.2** |
| Without comorbidities of respiratory system diseases |  | 11806 (91.0) | 11406 (90.9) | 400 (92.8) |  |
| With one comorbidity of respiratory system diseases |  | 953 (7.3) | 932 (7.4) | 21 (4.9) |  |
| With two comorbidities of respiratory system diseases |  | 187 (1.4) | 177 (1.4) | 10 (2.3) |  |
| With three comorbidities of respiratory system diseases |  | 25 (0.2) | 25 (0.2) | 0 (0.0) |  |
| With four comorbidities of respiratory system diseases |  | 4 (0.0) | 4 (0.0) | 0 (0.0) |  |
| With five comorbidities of respiratory system diseases |  | 1 (0.0) | 1 (0.0) | 0 (0.0) |  |
| **Number of circulatory comorbidity (%)** | **circulatory_comorbidity_NO** |  |  |  | **0.538** |
| Without comorbidities of circulatory system diseases |  | 10178 (78.4) | 9826 (78.3) | 352 (81.7) |  |
| With one comorbidity of circulatory system diseases |  | 1900 (14.6) | 1840 (14.7) | 60 (13.9) |  |
| With two comorbidities of circulatory system diseases |  | 622 (4.8) | 608 (4.8) | 14 (3.2) |  |
| With three comorbidities of circulatory system diseases |  | 208 (1.6) | 204 (1.6) | 4 (0.9) |  |
| With four comorbidities of circulatory system diseases |  | 58 (0.4) | 57 (0.5) | 1 (0.2) |  |
| With five comorbidities of circulatory system diseases |  | 9 (0.1) | 9 (0.1) | 0 (0.0) |  |
| With six comorbidities of circulatory system diseases |  | 1 (0.0) | 1 (0.0) | 0 (0.0) |  |
| **Number of digestive comorbidity (%)** | **digestive_comorbidity_NO** |  |  |  | **0.041** |
| Without comorbidities of digestive system diseases |  | 10980 (84.6) | 10602 (84.5) | 378 (87.7) |  |
| With one comorbidity of digestive system diseases |  | 1529 (11.8) | 1489 (11.9) | 40 (9.3) |  |
| With two comorbidities of digestive system diseases |  | 364 (2.8) | 353 (2.8) | 11 (2.6) |  |
| With three comorbidities of digestive system diseases |  | 78 (0.6) | 77 (0.6) | 1 (0.2) |  |
| With four comorbidities of digestive system diseases |  | 21 (0.2) | 21 (0.2) | 0 (0.0) |  |
| With five comorbidities of digestive system diseases |  | 3 (0.0) | 2 (0.0) | 1 (0.2) |  |
| With six comorbidities of digestive system diseases |  | 1 (0.0) | 1 (0.0) | 0 (0.0) |  |
| **Number of nervous comorbidity (%)** | **nervous_comorbidity_NO** |  |  |  | **0.118** |
| Without comorbidities of nervous system diseases |  | 12003 (92.5) | 11604 (92.5) | 399 (92.6) |  |
| With one comorbidity of nervous system diseases |  | 848 (6.5) | 824 (6.6) | 24 (5.6) |  |
| With two comorbidities of nervous system diseases |  | 105 (0.8) | 97 (0.8) | 8 (1.9) |  |
| With three comorbidities of nervous system diseases |  | 19 (0.1) | 19 (0.2) | 0 (0.0) |  |
| With four comorbidities of nervous system diseases |  | 1 (0.0) | 1 (0.0) | 0 (0.0) |  |
| **Number of endocrine comorbidity (%)** | **endocrine_comorbidity_NO** |  |  |  | **0.855** |
| Without comorbidities of endocrine system diseases |  | 10648 (82.1) | 10295 (82.1) | 353 (81.9) |  |
| With one comorbidity of endocrine system diseases |  | 1887 (14.5) | 1822 (14.5) | 65 (15.1) |  |
| With two comorbidities of endocrine system diseases |  | 370 (2.9) | 360 (2.9) | 10 (2.3) |  |
| With three comorbidities of endocrine system diseases |  | 60 (0.5) | 58 (0.5) | 2 (0.5) |  |
| With four comorbidities of endocrine system diseases |  | 10 (0.1) | 9 (0.1) | 1 (0.2) |  |
| With five comorbidities of endocrine system diseases |  | 1 (0.0) | 1 (0.0) | 0 (0.0) |  |
| **Number of psychiatric comorbidity (%)** | **psychiatric_comorbidity_NO** |  |  |  | **<0.001** |
| Without comorbidities of psychiatric diseases |  | 8232 (63.4) | 7896 (62.9) | 336 (78.0) |  |
| With one comorbidity of psychiatric diseases |  | 4292 (33.1) | 4202 (33.5) | 90 (20.9) |  |
| With two comorbidities of psychiatric diseases |  | 427 (3.3) | 422 (3.4) | 5 (1.2) |  |
| With three comorbidities of psychiatric diseases |  | 25 (0.2) | 25 (0.2) | 0 (0.0) |  |
| ***Prior history variables*** | | |  |  |  |
| **History of allergy (%)** | **history_allergy** |  |  |  | **0.762** |
| No |  | 11159 (86.0) | 10791 (86.0) | 368 (85.4) |  |
| Yes |  | 1817 (14.0) | 1754 (14.0) | 63 (14.6) |  |
| **History of blood transfusion (%)** | **history_blood_transfusion** |  |  |  | **0.521** |
| No |  | 12495 (96.3) | 12077 (96.3) | 418 (97.0) |  |
| Yes |  | 481 (3.7) | 468 (3.7) | 13 (3.0) |  |
| **History of drug use (%)** | **history_drug_use** |  |  |  | **0.054** |
| No |  | 10377 (80.0) | 10049 (80.1) | 328 (76.1) |  |
| Occasionally |  | 632 (4.9) | 612 (4.9) | 20 (4.6) |  |
| Frequently |  | 1967 (15.2) | 1884 (15.0) | 83 (19.3) |  |
| **History of surgery (%)** | **history_surgery** |  |  |  | **0.28** |
| No |  | 8124 (62.6) | 7843 (62.5) | 281 (65.2) |  |
| Yes |  | 4852 (37.4) | 4702 (37.5) | 150 (34.8) |  |
| **History of smoking (%)** | **history_smoking** |  |  |  | **<0.001** |
| No |  | 12304 (94.8) | 11875 (94.7) | 429 (99.5) |  |
| Yes |  | 672 (5.2) | 670 (5.3) | 2 (0.5) |  |
| **History of alcoholism (%)** | **history_alcoholism** |  |  |  | **<0.001** |
| No |  | 12464 (96.1) | 12035 (95.9) | 429 (99.5) |  |
| Yes |  | 512 (3.9) | 510 (4.1) | 2 (0.5) |  |
| ***Vital signs variables*** | | |  |  |  |
| **Physical examination: to record whether a patient has subcutaneous bleeding (%)** | **PE_subcutaneous.bleeding** |  |  |  | **0.732** |
| No |  | 12883 (99.3) | 12454 (99.3) | 429 (99.5) |  |
| Yes |  | 93 (0.7) | 91 (0.7) | 2 (0.5) |  |
| **Physical examination: to record a patient's facial expression (%)** | **PE_expression** |  |  |  | **0.095** |
| Natural |  | 12421 (95.7) | 11996 (95.6) | 425 (98.6) |  |
| Indifferent |  | 184 (1.4) | 183 (1.5) | 1 (0.2) |  |
| Dreadful |  | 1 (0.0) | 1 (0.0) | 0 (0.0) |  |
| Painful |  | 123 (0.9) | 121 (1.0) | 2 (0.5) |  |
| Anxious |  | 244 (1.9) | 241 (1.9) | 3 (0.7) |  |
| Exciting |  | 3 (0.0) | 3 (0.0) | 0 (0.0) |  |
| **Physical examination: to record a patient's sickly look (%)** | **PE_face** |  |  |  | **<0.001** |
| Normal |  | 11646 (89.8) | 11228 (89.5) | 418 (97.0) |  |
| Chronic |  | 1002 (7.7) | 994 (7.9) | 8 (1.9) |  |
| Acute |  | 275 (2.1) | 271 (2.2) | 4 (0.9) |  |
| Other |  | 53 (0.4) | 52 (0.4) | 1 (0.2) |  |
| **Physical examination: to record a patient's nutrition status (%)** | **PE_nutrition** |  |  |  | **0.327** |
| Good |  | 12017 (92.6) | 11613 (92.6) | 404 (93.7) |  |
| Medium |  | 728 (5.6) | 710 (5.7) | 18 (4.2) |  |
| Malnourished |  | 222 (1.7) | 214 (1.7) | 8 (1.9) |  |
| Cachectic |  | 9 (0.1) | 8 (0.1) | 1 (0.2) |  |
| **Physical examination: to record whether a patient cooperates with the examination (%)** | **PE_cooperation** |  |  |  | **0.026** |
| No |  | 12727 (98.1) | 12311 (98.1) | 416 (96.5) |  |
| Yes |  | 249 (1.9) | 234 (1.9) | 15 (3.5) |  |
| **Physical examination: to record a patient's consciousness (%)** | **PE_consciousness** |  |  |  | **0.829** |
| Aware |  | 12914 (99.5) | 12483 (99.5) | 431 (100.0) |  |
| Confused |  | 23 (0.2) | 23 (0.2) | 0 (0.0) |  |
| Somnolence |  | 23 (0.2) | 23 (0.2) | 0 (0.0) |  |
| Stupor |  | 6 (0.0) | 6 (0.0) | 0 (0.0) |  |
| Coma |  | 6 (0.0) | 6 (0.0) | 0 (0.0) |  |
| Delirium |  | 4 (0.0) | 4 (0.0) | 0 (0.0) |  |
| **Physical examination: to record a patient's gait (%)** | **PE_gait** |  |  |  | **<0.001** |
| Normal |  | 12471 (96.1) | 12042 (96.0) | 429 (99.5) |  |
| Abnormal |  | 505 (3.9) | 503 (4.0) | 2 (0.5) |  |
| **Physical examination: to record a patient's body position (%)** | **PE_body.position** |  |  |  | **0.208** |
| Natural |  | 12762 (98.4) | 12333 (98.3) | 429 (99.5) |  |
| Passive |  | 163 (1.3) | 162 (1.3) | 1 (0.2) |  |
| Coercive |  | 22 (0.2) | 22 (0.2) | 0 (0.0) |  |
| Other |  | 29 (0.2) | 28 (0.2) | 1 (0.2) |  |
| **Physical examination: to record a patient's body temperature (median [IQR])** | **PE_body.temperature** | 36.50 [36.40, 36.70] | 36.50 [36.40, 36.70] | 36.50 [36.40, 36.70] | **0.472** |
| **Physical examination: to record a patient's pulse (median [IQR])** | **PE_pulse** | 80.00 [73.00, 88.00] | 80.00 [72.00, 88.00] | 80.00 [75.00, 90.00] | **0.002** |
| **Physical examination: to record a patient's breath (median [IQR])** | **PE_breath** | 20.00 [20.00, 20.00] | 20.00 [20.00, 20.00] | 20.00 [20.00, 20.00] | **0.664** |
| **Physical examination: to record a patient's systolic blood pressure (SBP) (median [IQR])** | **PE_SBP** | 120.00 [111.00, 132.00] | 120.00 [111.00, 132.00] | 120.00 [110.00, 131.00] | **0.269** |
| **Physical examination: to record a patient's diastolic blood pressure (DBP) (median [IQR])** | **PE_DBP** | 77.00 [70.00, 84.00] | 77.00 [70.00, 84.00] | 77.00 [70.00, 84.00] | **0.804** |
| ***Symptom variables*** | | |  |  |  |
| **Whether the patient has a symptom of mood-down (%)** | **mood** |  |  |  | **<0.001** |
| No |  | 4929 (38.0) | 4832 (38.5) | 97 (22.5) |  |
| Yes |  | 8047 (62.0) | 7713 (61.5) | 334 (77.5) |  |
| **Whether the patient has a symptom of bad sleep (%)** | **bad_sleep** |  |  |  | **0.097** |
| No |  | 8332 (64.2) | 8072 (64.3) | 260 (60.3) |  |
| Yes |  | 4644 (35.8) | 4473 (35.7) | 171 (39.7) |  |
| **Whether the patient has a symptom of loss of interest (%)** | **loss_interest** |  |  |  | **0.651** |
| No |  | 11572 (89.2) | 11191 (89.2) | 381 (88.4) |  |
| Yes |  | 1404 (10.8) | 1354 (10.8) | 50 (11.6) |  |
| **Whether the patient has a symptom of flustered (%)** | **flustered** |  |  |  | **0.015** |
| No |  | 11559 (89.1) | 11191 (89.2) | 368 (85.4) |  |
| Yes |  | 1417 (10.9) | 1354 (10.8) | 63 (14.6) |  |
| **Whether the patient has a symptom of worry (%)** | **worry** |  |  |  | **0.225** |
| No |  | 12204 (94.1) | 11805 (94.1) | 399 (92.6) |  |
| Yes |  | 772 (5.9) | 740 (5.9) | 32 (7.4) |  |
| **Whether the patient has a symptom of tension (%)** | **tension** |  |  |  | **0.403** |
| No |  | 12265 (94.5) | 11862 (94.6) | 403 (93.5) |  |
| Yes |  | 711 (5.5) | 683 (5.4) | 28 (6.5) |  |
| **Whether the patient has a symptom of upset (%)** | **upset** |  |  |  | **0.735** |
| No |  | 12051 (92.9) | 11653 (92.9) | 398 (92.3) |  |
| Yes |  | 925 (7.1) | 892 (7.1) | 33 (7.7) |  |
| **Whether the patient has a symptom of headache (%)** | **headache** |  |  |  | **0.456** |
| No |  | 12351 (95.2) | 11937 (95.2) | 414 (96.1) |  |
| Yes |  | 625 (4.8) | 608 (4.8) | 17 (3.9) |  |
| **Whether the patient has a symptom of dizziness (%)** | **dizziness** |  |  |  | **0.003** |
| No |  | 12072 (93.0) | 11655 (92.9) | 417 (96.8) |  |
| Yes |  | 904 (7.0) | 890 (7.1) | 14 (3.2) |  |
| **Whether the patient has a symptom of physical discomfort (%)** | **physical_discomfort** |  |  |  | **0.038** |
| No |  | 12183 (93.9) | 11789 (94.0) | 394 (91.4) |  |
| Yes |  | 793 (6.1) | 756 (6.0) | 37 (8.6) |  |
| **Whether the patient has a symptom of fatigue (%)** | **fatigue** |  |  |  | **0.38** |
| No |  | 12398 (95.5) | 11982 (95.5) | 416 (96.5) |  |
| Yes |  | 578 (4.5) | 563 (4.5) | 15 (3.5) |  |
| **Whether the patient has a symptom of suicide ideation (%)** | **suicide** |  |  |  | **0.205** |
| No |  | 12452 (96.0) | 12044 (96.0) | 408 (94.7) |  |
| Yes |  | 524 (4.0) | 501 (4.0) | 23 (5.3) |  |
| **Whether the patient has a symptom of self-harm (%)** | **self_harm** |  |  |  | **0.955** |
| No |  | 12844 (99.0) | 12418 (99.0) | 426 (98.8) |  |
| Yes |  | 132 (1.0) | 127 (1.0) | 5 (1.2) |  |
| **Whether the patient has a symptom of hallucination (%)** | **hallucination** |  |  |  | **0.355** |
| No |  | 12731 (98.1) | 12311 (98.1) | 420 (97.4) |  |
| Yes |  | 245 (1.9) | 234 (1.9) | 11 (2.6) |  |
| **Whether the patient has a symptom of less activity (%)** | **less_activity** |  |  |  | **0.875** |
| No |  | 12527 (96.5) | 12112 (96.5) | 415 (96.3) |  |
| Yes |  | 449 (3.5) | 433 (3.5) | 16 (3.7) |  |
| **Whether the patient has a symptom of chest tightness (%)** | **chest_tightness** |  |  |  | **0.657** |
| No |  | 12615 (97.2) | 12194 (97.2) | 421 (97.7) |  |
| Yes |  | 361 (2.8) | 351 (2.8) | 10 (2.3) |  |
| **Whether the patient has a symptom of afraid (%)** | **afraid** |  |  |  | **0.007** |
| No |  | 12794 (98.6) | 12376 (98.7) | 418 (97.0) |  |
| Yes |  | 182 (1.4) | 169 (1.3) | 13 (3.0) |  |
| **Whether the patient has a symptom of irritability (%)** | **irritability** |  |  |  | **0.64** |
| No |  | 12808 (98.7) | 12381 (98.7) | 427 (99.1) |  |
| Yes |  | 168 (1.3) | 164 (1.3) | 4 (0.9) |  |
| **Whether the patient has a symptom of fidget (%)** | **fidget** |  |  |  | **0.152** |
| No |  | 12817 (98.8) | 12395 (98.8) | 422 (97.9) |  |
| Yes |  | 159 (1.2) | 150 (1.2) | 9 (2.1) |  |
| **Whether the patient has a symptom of slow response (%)** | **slow_response** |  |  |  | **0.125** |
| No |  | 12832 (98.9) | 12402 (98.9) | 430 (99.8) |  |
| Yes |  | 144 (1.1) | 143 (1.1) | 1 (0.2) |  |
| **Whether the patient has a recurrence of symptoms (%)** | **relapse** |  |  |  | **<0.001** |
| No |  | 10727 (82.7) | 10418 (83.0) | 309 (71.7) |  |
| Yes |  | 2249 (17.3) | 2127 (17.0) | 122 (28.3) |  |
| **Whether the patient has a worsen of symptoms (%)** | **symp_worsen** |  |  |  | **0.291** |
| No |  | 8008 (61.7) | 7753 (61.8) | 255 (59.2) |  |
| Yes |  | 4968 (38.3) | 4792 (38.2) | 176 (40.8) |  |
| **Number of core symptoms** **(including mood-down and loss of interest) for each patient (%)** | **core_symp** |  |  |  | **<0.001** |
| Without core symptoms |  | 4799 (37.0) | 4708 (37.5) | 91 (21.1) |  |
| With one core symptom |  | 6903 (53.2) | 6607 (52.7) | 296 (68.7) |  |
| With two core symptoms |  | 1274 (9.8) | 1230 (9.8) | 44 (10.2) |  |
| **Number of psychological symptoms (including** **worry, tension, upset, suicide ideation, self-harm, hallucination, less activity, afraid, irritability, fidget, and slow response) for each patient (%)** | **psy_symp** |  |  |  | **0.008** |
| Without psychological symptoms |  | 9467 (73.0) | 9166 (73.1) | 301 (69.8) |  |
| With one psychological symptom |  | 2670 (20.6) | 2580 (20.6) | 90 (20.9) |  |
| With two psychological symptoms |  | 779 (6.0) | 743 (5.9) | 36 (8.4) |  |
| With three psychological symptoms |  | 57 (0.4) | 54 (0.4) | 3 (0.7) |  |
| With four psychological symptoms |  | 3 (0.0) | 2 (0.0) | 1 (0.2) |  |
| **Number of physical symptoms** **(including flustered, bad sleep, headache, dizziness, physical discomfort, fatigue, and chest tightness) for each patient (%)** | **phy_symp** |  |  |  | **0.743** |
| Without physical symptoms |  | 6052 (46.6) | 5858 (46.7) | 194 (45.0) |  |
| With one physical symptom |  | 4927 (38.0) | 4762 (38.0) | 165 (38.3) |  |
| With two physical symptoms |  | 1632 (12.6) | 1577 (12.6) | 55 (12.8) |  |
| With three physical symptoms |  | 330 (2.5) | 314 (2.5) | 16 (3.7) |  |
| With four physical symptoms |  | 34 (0.3) | 33 (0.3) | 1 (0.2) |  |
| With five physical symptoms |  | 1 (0.0) | 1 (0.0) | 0 (0.0) |  |
| **Combination of key symptoms for each patient (%)** | **symptom** |  |  |  | **<0.001** |
| Without any key words (mood-down, bad sleep, etc.) of symptoms below |  | 1835 (14.1) | 1801 (14.4) | 34 (7.9) |  |
| Mainly mood-related symptoms, accompanied by other symptoms |  | 8047 (62.0) | 7713 (61.5) | 334 (77.5) |  |
| Mainly sleep-related symptoms, accompanied by other symptoms (not including mood-related) |  | 802 (6.2) | 778 (6.2) | 24 (5.6) |  |
| Mainly loss of interest-related symptoms, accompanied by other symptoms (not including mood, sleep-related) |  | 65 (0.5) | 63 (0.5) | 2 (0.5) |  |
| Mainly flustered-related symptoms, accompanied by other symptoms (not including mood, sleep, interest-related) |  | 195 (1.5) | 191 (1.5) | 4 (0.9) |  |
| Mainly worry-related symptoms, accompanied by other symptoms (not including mood, sleep, interest, flustered-related) |  | 67 (0.5) | 62 (0.5) | 5 (1.2) |  |
| Mainly tension-related symptoms, accompanied by other symptoms (not including mood, sleep, interest, flustered, worry-related) |  | 33 (0.3) | 30 (0.2) | 3 (0.7) |  |
| Mainly upset-related symptoms, accompanied by other symptoms (not including mood, sleep, interest, flustered, worry, tension-related) |  | 42 (0.3) | 40 (0.3) | 2 (0.5) |  |
| Mainly headache-related symptoms, accompanied by other symptoms (not including mood, sleep, interest, flustered, worry, tension, upset-related) |  | 289 (2.2) | 286 (2.3) | 3 (0.7) |  |
| Mainly dizziness-related symptoms, accompanied by other symptoms (not including mood, sleep, interest, flustered, worry, tension, upset, headache-related) |  | 335 (2.6) | 335 (2.7) | 0 (0.0) |  |
| Mainly physical discomfort-related symptoms, accompanied by other symptoms (not including mood, sleep, interest, flustered, worry, tension, upset, headache, dizziness-related) |  | 88 (0.7) | 87 (0.7) | 1 (0.2) |  |
| Mainly fatigue-related symptoms, accompanied by other symptoms (not including mood, sleep, interest, flustered, worry, tension, upset, headache, dizziness, physical discomfort-related) |  | 188 (1.4) | 188 (1.5) | 0 (0.0) |  |
| Mainly suicide and self-harm related symptoms, accompanied by other symptoms (not including mood, sleep, interest, flustered, worry, tension, upset, headache, dizziness, physical discomfort, fatigue-related) |  | 36 (0.3) | 35 (0.3) | 1 (0.2) |  |
| Mainly hallucination-related symptoms, accompanied by other symptoms (not including mood, sleep, interest, flustered, worry, tension, upset, headache, dizziness, physical discomfort, fatigue, suicide, and self-harm related) |  | 44 (0.3) | 44 (0.4) | 0 (0.0) |  |
| Mainly less activity-related symptoms, accompanied by other symptoms (not including mood, sleep, interest, flustered, worry, tension, upset, headache, dizziness, physical discomfort, fatigue, suicide and self-harm, hallucination related) |  | 112 (0.9) | 111 (0.9) | 1 (0.2) |  |
| Mainly chest tightness-related symptoms, accompanied by other symptoms (not including mood, sleep, interest, flustered, worry, tension, upset, headache, dizziness, physical discomfort, fatigue, suicide and self-harm, hallucination, less activity related) |  | 90 (0.7) | 89 (0.7) | 1 (0.2) |  |
| Mainly slow response, afraid, fidget, and irritability related symptoms, accompanied by other symptoms (not including mood, sleep, interest, flustered, worry, tension, upset, headache, dizziness, physical discomfort, fatigue, suicide and self-harm, hallucination, less activity, chest tightness related) |  | 86 (0.7) | 85 (0.7) | 1 (0.2) |  |
| Mainly relapse of symptoms |  | 160 (1.2) | 153 (1.2) | 7 (1.6) |  |
| Mainly worsen of symptoms |  | 462 (3.6) | 454 (3.6) | 8 (1.9) |  |
| **Severity score of all symptoms (median [IQR])** | **sym_final_score** | 0.01 [0.00, 0.06] | 0.01 [0.00, 0.06] | 0.01 [0.00, 0.06] | **0.036** |
| ***Treatment-related variables*** | | |  |  |  |
| **Type of antidepressants used for each patient (%)** | **ADP_type** |  |  |  | **<0.001** |
| Without any ADP drugs used during this admission |  | 1625 (12.5) | 1596 (12.7) | 29 (6.7) |  |
| With one type of ADP drugs used during this admission |  | 9225 (71.1) | 8939 (71.3) | 286 (66.4) |  |
| With two types of ADP drugs used during this admission |  | 1870 (14.4) | 1767 (14.1) | 103 (23.9) |  |
| With three types of ADP drugs used during this admission |  | 231 (1.8) | 219 (1.7) | 12 (2.8) |  |
| With four types of ADP drugs used during this admission |  | 23 (0.2) | 22 (0.2) | 1 (0.2) |  |
| With six types of ADP drugs used during this admission |  | 2 (0.0) | 2 (0.0) | 0 (0.0) |  |
| **Type of antipsychotics used for each patient (%)** | **AP_type** |  |  |  | **<0.001** |
| Without any AP drugs used during this admission |  | 5153 (39.7) | 5064 (40.4) | 89 (20.6) |  |
| With one type of AP drugs used during this admission |  | 5480 (42.2) | 5265 (42.0) | 215 (49.9) |  |
| With two types of AP drugs used during this admission |  | 1794 (13.8) | 1703 (13.6) | 91 (21.1) |  |
| With three types of AP drugs used during this admission |  | 432 (3.3) | 406 (3.2) | 26 (6.0) |  |
| With four types of AP drugs used during this admission |  | 99 (0.8) | 90 (0.7) | 9 (2.1) |  |
| With five types of AP drugs used during this admission |  | 13 (0.1) | 12 (0.1) | 1 (0.2) |  |
| With six types of AP drugs used during this admission |  | 3 (0.0) | 3 (0.0) | 0 (0.0) |  |
| With seven types of AP drugs used during this admission |  | 2 (0.0) | 2 (0.0) | 0 (0.0) |  |
| **Type of anxiolytics used for each patient (%)** | **AA_type** |  |  |  | **<0.001** |
| Without any AA drugs used during this admission |  | 1945 (15.0) | 1911 (15.2) | 34 (7.9) |  |
| With one type of AA drugs used during this admission |  | 5500 (42.4) | 5344 (42.6) | 156 (36.2) |  |
| With two types of AA drugs used during this admission |  | 3669 (28.3) | 3534 (28.2) | 135 (31.3) |  |
| With three types of AA drugs used during this admission |  | 1452 (11.2) | 1378 (11.0) | 74 (17.2) |  |
| With four types of AA drugs used during this admission |  | 348 (2.7) | 320 (2.6) | 28 (6.5) |  |
| With five types of AA drugs used during this admission |  | 50 (0.4) | 46 (0.4) | 4 (0.9) |  |
| With six types of AA drugs used during this admission |  | 10 (0.1) | 10 (0.1) | 0 (0.0) |  |
| With seven types of AA drugs used during this admission |  | 2 (0.0) | 2 (0.0) | 0 (0.0) |  |
| **Type of mood stabilizers used for each patient (%)** | **MSB_type** |  |  |  | **0.052** |
| Without any MSB drugs used during this admission |  | 11679 (90.0) | 11308 (90.1) | 371 (86.1) |  |
| With one type of MSB drugs used during this admission |  | 1150 (8.9) | 1099 (8.8) | 51 (11.8) |  |
| With two types of MSB drugs used during this admission |  | 142 (1.1) | 133 (1.1) | 9 (2.1) |  |
| With three types of MSB drugs used during this admission |  | 4 (0.0) | 4 (0.0) | 0 (0.0) |  |
| With four types of MSB drugs used during this admission |  | 1 (0.0) | 1 (0.0) | 0 (0.0) |  |
| **Type of anti-side effects drugs used for each patient (%)** | **ASE_type** |  |  |  | **0.01** |
| Without any ASE drugs used during this admission |  | 8276 (63.8) | 8026 (64.0) | 250 (58.0) |  |
| With one type of ASE drugs used during this admission |  | 2733 (21.1) | 2634 (21.0) | 99 (23.0) |  |
| With two types of ASE drugs used during this admission |  | 1330 (10.2) | 1279 (10.2) | 51 (11.8) |  |
| With three types of ASE drugs used during this admission |  | 464 (3.6) | 446 (3.6) | 18 (4.2) |  |
| With four types of ASE drugs used during this admission |  | 151 (1.2) | 138 (1.1) | 13 (3.0) |  |
| With five types of ASE drugs used during this admission |  | 17 (0.1) | 17 (0.1) | 0 (0.0) |  |
| With six types of ASE drugs used during this admission |  | 4 (0.0) | 4 (0.0) | 0 (0.0) |  |
| With seven types of ASE drugs used during this admission |  | 1 (0.0) | 1 (0.0) | 0 (0.0) |  |
| **Type of new hypnotics used for each patient (%)** | **HYP_type** |  |  |  | **0.014** |
| Without any HYP drugs used during this admission |  | 12253 (94.4) | 11858 (94.5) | 395 (91.6) |  |
| With one type of HYP drugs used during this admission |  | 723 (5.6) | 687 (5.5) | 36 (8.4) |  |
| **Type of β receptor blockers used for each patient (%)** | **OT_type** |  |  |  | **0.001** |
| Without any OT drugs used during this admission |  | 10592 (81.6) | 10269 (81.9) | 323 (74.9) |  |
| With one type of OT drugs used during this admission |  | 2296 (17.7) | 2193 (17.5) | 103 (23.9) |  |
| With two types of OT drugs used during this admission |  | 88 (0.7) | 83 (0.7) | 5 (1.2) |  |
| **Type of hormonal drugs used for each patient (%)** | **T3_type** |  |  |  | **0.051** |
| Without any T3 drugs used during this admission |  | 12616 (97.2) | 12204 (97.3) | 412 (95.6) |  |
| With one type of T3 drugs used during this admission |  | 360 (2.8) | 341 (2.7) | 19 (4.4) |  |
| **Type of Chinese patent medicines used for each patient (%)** | **CM_type** |  |  |  | **<0.001** |
| Without any CM drugs used during this admission |  | 11160 (86.0) | 10822 (86.3) | 338 (78.4) |  |
| With one type of CM drugs used during this admission |  | 1816 (14.0) | 1723 (13.7) | 93 (21.6) |  |
| **Type of physiotherapies used for each patient (%)** | **PHY_type** |  |  |  | **<0.001** |
| Without any physiotherapies used during this admission |  | 7129 (54.9) | 6945 (55.4) | 184 (42.7) |  |
| With one type of physiotherapies used during this admission |  | 4878 (37.6) | 4671 (37.2) | 207 (48.0) |  |
| With two types of physiotherapies used during this admission |  | 816 (6.3) | 783 (6.2) | 33 (7.7) |  |
| With three types of physiotherapies used during this admission |  | 143 (1.1) | 136 (1.1) | 7 (1.6) |  |
| With four types of physiotherapies used during this admission |  | 10 (0.1) | 10 (0.1) | 0 (0.0) |  |
| **Type of psychotherapies used for each patient (%)** | **PSY_type** |  |  |  | **0.007** |
| Without any psychotherapies used during this admission |  | 6322 (48.7) | 6140 (48.9) | 182 (42.2) |  |
| With one type of psychotherapies used during this admission |  | 6654 (51.3) | 6405 (51.1) | 249 (57.8) |  |
| **Combination of all therapy types for each patient (%)** | **combo_treat** |  |  |  | **<0.001** |
| Without any anti-depression therapy |  | 429 (3.3) | 420 (3.3) | 9 (2.1) |  |
| Mainly the combination of ADP, AP, AA, MSB, and ASE drugs, accompanied by other therapies |  | 350 (2.7) | 319 (2.5) | 31 (7.2) |  |
| Mainly the combination of ADP, AP, AA, and MSB drugs, accompanied by other therapies except for ASE drugs |  | 553 (4.3) | 534 (4.3) | 19 (4.4) |  |
| Mainly the combination of ADP, AP, AA, and ASE drugs, accompanied by other therapies except for MSB drugs |  | 2474 (19.1) | 2351 (18.7) | 123 (28.5) |  |
| Mainly the combination of ADP, AP, AA, and HYP drugs, accompanied by other therapies except for MSB and ASE drugs |  | 226 (1.7) | 221 (1.8) | 5 (1.2) |  |
| Mainly the combination of ADP, AP, AA, and CM drugs, accompanied by other therapies except for MSB, ASE and HYP drugs |  | 509 (3.9) | 482 (3.8) | 27 (6.3) |  |
| Mainly the combination of ADP, AP, AA, and OT drugs, accompanied by other therapies except for MSB, ASE, HYP and CM drugs |  | 504 (3.9) | 483 (3.9) | 21 (4.9) |  |
| Mainly the combination of ADP, AP and AA drugs, accompanied by other therapies except for MSB, ASE, HYP, CM and OT drugs |  | 2345 (18.1) | 2257 (18.0) | 88 (20.4) |  |
| Mainly the combination of ADP and AP drugs, accompanied by other therapies except for AA drugs |  | 543 (4.2) | 526 (4.2) | 17 (3.9) |  |
| Mainly the combination of ADP and AA drugs, accompanied by other therapies except for AP drugs |  | 3140 (24.2) | 3075 (24.5) | 65 (15.1) |  |
| Mainly the ADP drugs, accompanied by other therapies except for AP and AA drugs |  | 707 (5.4) | 701 (5.6) | 6 (1.4) |  |
| Mainly the combination of AP and AA drugs, accompanied by other therapies except for ADP drugs |  | 272 (2.1) | 262 (2.1) | 10 (2.3) |  |
| Mainly the AA drugs, accompanied by other therapies except for ADP and AP drugs |  | 658 (5.1) | 650 (5.2) | 8 (1.9) |  |
| Mainly the AP drugs, accompanied by other therapies except for ADP and AA drugs |  | 47 (0.4) | 46 (0.4) | 1 (0.2) |  |
| Mainly the ASE drugs, accompanied by other therapies except for ADP, AP, and AA drugs |  | 160 (1.2) | 159 (1.3) | 1 (0.2) |  |
| Mainly the MSB, HYP, CM, OT and T3 drugs, accompanied by other therapies except for ADP, AP, AA, and ASE drugs |  | 41 (0.3) | 41 (0.3) | 0 (0.0) |  |
| Mainly the physiotherapies and psychotherapies, without any drugs |  | 18 (0.1) | 18 (0.1) | 0 (0.0) |  |
| **Combination of drug types first used for over three days in the whole treatment path for each patient (%)** | **main_drug** |  |  |  | **<0.001** |
| Without any drugs used |  | 916 (7.1) | 900 (7.2) | 16 (3.7) |  |
| Mainly the AA drugs, accompanied by other drugs except for ADP and AP drugs |  | 758 (5.8) | 739 (5.9) | 19 (4.4) |  |
| Mainly the combination of ADP and AA drugs, accompanied by other drugs except for AP drugs |  | 4138 (31.9) | 4004 (31.9) | 134 (31.1) |  |
| Mainly the combination of AP and AA drugs, accompanied by other drugs except for ADP drugs |  | 299 (2.3) | 288 (2.3) | 11 (2.6) |  |
| Mainly the combination of ADP, AP, and AA drugs, accompanied by other drugs |  | 4045 (31.2) | 3858 (30.8) | 187 (43.4) |  |
| Mainly the combination of ADP and AP drugs, accompanied by other drugs except for AA drugs |  | 1063 (8.2) | 1025 (8.2) | 38 (8.8) |  |
| Mainly the ADP drugs, accompanied by other drugs except for AP and AA drugs |  | 1010 (7.8) | 994 (7.9) | 16 (3.7) |  |
| Mainly the AP drugs, accompanied by other drugs except for ADP and AA drugs |  | 196 (1.5) | 190 (1.5) | 6 (1.4) |  |
| Mainly the ASE drugs, accompanied by other drugs except for ADP, AP, and AA drugs |  | 406 (3.1) | 404 (3.2) | 2 (0.5) |  |
| Other |  | 145 (1.1) | 143 (1.1) | 2 (0.5) |  |
| **Sum of medical orders prescribed for each patient (median [IQR])** | **order_SUM** | 511.00 [373.00, 712.00] | 508.00 [372.00, 703.00] | 648.00 [398.00, 953.00] | **<0.001** |
| **Sum of antidepressant orders prescribed for each patient (median [IQR])** | **ADP_SUM** | 19.00 [10.00, 28.00] | 19.00 [10.00, 28.00] | 24.00 [14.00, 36.00] | **<0.001** |
| **Number of orders of amitriptyline hydrochloride tablets prescribed for each patient (median [IQR])** | **AMTL** | 0.00 [0.00, 0.00] | 0.00 [0.00, 0.00] | 0.00 [0.00, 0.00] | **0.223** |
| **Number of orders of escitalopram oxalate tablets prescribed for each patient (median [IQR])** | **ASXTPL** | 0.00 [0.00, 0.00] | 0.00 [0.00, 0.00] | 0.00 [0.00, 0.00] | **0.279** |
| **Number of orders of duloxetine hydrochloride enteric-coated capsules prescribed for each patient (median [IQR])** | **DLXT** | 0.00 [0.00, 0.00] | 0.00 [0.00, 0.00] | 0.00 [0.00, 0.00] | **0.373** |
| **Number of orders of fluoxetine hydrochloride dispersible tablets prescribed for each patient (median [IQR])** | **FXT** | 0.00 [0.00, 0.00] | 0.00 [0.00, 0.00] | 0.00 [0.00, 0.00] | **<0.001** |
| **Number of orders of clomipramine hydrochloride tablets prescribed for each patient (median [IQR])** | **LMPM** | 0.00 [0.00, 0.00] | 0.00 [0.00, 0.00] | 0.00 [0.00, 0.00] | **0.835** |
| **Number of orders of paroxetine hydrochloride tablets prescribed for each patient (median [IQR])** | **PLXT** | 0.00 [0.00, 0.00] | 0.00 [0.00, 0.00] | 0.00 [0.00, 0.00] | **0.742** |
| **Number of orders of sertraline hydrochloride tablets prescribed for each patient (median [IQR])** | **SQL** | 0.00 [0.00, 4.00] | 0.00 [0.00, 4.00] | 0.00 [0.00, 8.00] | **0.126** |
| **Number of orders of venlafaxine hydrochloride capsules prescribed for each patient (median [IQR])** | **WLFX** | 0.00 [0.00, 0.00] | 0.00 [0.00, 0.00] | 0.00 [0.00, 0.00] | **0.161** |
| **Number of orders of citalopram hydrobromide tablets prescribed for each patient (median [IQR])** | **XTPL** | 0.00 [0.00, 0.00] | 0.00 [0.00, 0.00] | 0.00 [0.00, 0.00] | **0.367** |
| **Number of orders of doxepine hydrochloride tablets prescribed for each patient (median [IQR])** | **YSDSPP** | 0.00 [0.00, 0.00] | 0.00 [0.00, 0.00] | 0.00 [0.00, 0.00] | **0.28** |
| **Number of orders of venlafaxine hydrochloride sustained-release capsules prescribed for each patient (median [IQR])** | **YSWLFX** | 0.00 [0.00, 3.00] | 0.00 [0.00, 2.00] | 0.00 [0.00, 12.00] | **<0.001** |
| **Sum of antipsychotic orders prescribed for each patient (median [IQR])** | **AP_SUM** | 11.00 [0.00, 22.00] | 10.00 [0.00, 21.00] | 19.00 [4.00, 32.00] | **<0.001** |
| **Number of orders of olanzapine tablets prescribed for each patient (median [IQR])** | **ADP** | 0.00 [0.00, 12.00] | 0.00 [0.00, 12.00] | 0.00 [0.00, 18.00] | **<0.001** |
| **Number of orders of aripiprazole orally disintegrating tablets prescribed for each patient (median [IQR])** | **ALPZKBP** | 0.00 [0.00, 0.00] | 0.00 [0.00, 0.00] | 0.00 [0.00, 0.00] | **0.637** |
| **Number of orders of quetiapine fumarate tablets prescribed for each patient (median [IQR])** | **FMSZLP** | 0.00 [0.00, 0.00] | 0.00 [0.00, 0.00] | 0.00 [0.00, 0.00] | **0.002** |
| **Number of orders of haloperidol for injection prescribed for each patient (median [IQR])** | **FPDCZSY** | 0.00 [0.00, 0.00] | 0.00 [0.00, 0.00] | 0.00 [0.00, 0.00] | **0.47** |
| **Number of orders of tiapride hydrochloride tablets prescribed for each patient (median [IQR])** | **LBL** | 0.00 [0.00, 0.00] | 0.00 [0.00, 0.00] | 0.00 [0.00, 0.00] | **0.675** |
| **Number of orders of clozapine tablets prescribed for each patient (median [IQR])** | **LDP** | 0.00 [0.00, 0.00] | 0.00 [0.00, 0.00] | 0.00 [0.00, 0.00] | **0.001** |
| **Number of orders of risperidone tablets prescribed for each patient (median [IQR])** | **LPT** | 0.00 [0.00, 0.00] | 0.00 [0.00, 0.00] | 0.00 [0.00, 0.00] | **0.061** |
| **Number of orders of sulpiride tablets prescribed for each patient (median [IQR])** | **SBL** | 0.00 [0.00, 0.00] | 0.00 [0.00, 0.00] | 0.00 [0.00, 0.00] | **0.04** |
| **Number of orders of sulpiride tablets (from another manufacture) prescribed for each patient (median [IQR])** | **SBLP** | 0.00 [0.00, 0.00] | 0.00 [0.00, 0.00] | 0.00 [0.00, 0.00] | **<0.001** |
| **Number of orders of sulpiride for injection prescribed for each patient (median [IQR])** | **SBLZSY** | 0.00 [0.00, 0.00] | 0.00 [0.00, 0.00] | 0.00 [0.00, 0.00] | **0.532** |
| **Number of orders of quetiapine fumarate tablets (from another manufacture) prescribed for each patient (median [IQR])** | **ZLP** | 0.00 [0.00, 0.00] | 0.00 [0.00, 0.00] | 0.00 [0.00, 0.00] | **<0.001** |
| **Sum of anxiolytic orders prescribed for each patient (median [IQR])** | **AA_SUM** | 22.00 [7.00, 38.00] | 22.00 [6.00, 37.00] | 30.00 [16.00, 49.00] | **<0.001** |
| **Number of orders of alprazolam tablets prescribed for each patient (median [IQR])** | **APZL** | 2.00 [0.00, 19.00] | 2.00 [0.00, 19.00] | 8.00 [0.00, 30.00] | **<0.001** |
| **Number of orders of estazolam tablets prescribed for each patient (median [IQR])** | **ASZL** | 0.00 [0.00, 0.00] | 0.00 [0.00, 0.00] | 0.00 [0.00, 0.00] | **0.513** |
| **Number of orders of diazepam tablets prescribed for each patient (median [IQR])** | **DXP** | 0.00 [0.00, 0.00] | 0.00 [0.00, 0.00] | 0.00 [0.00, 0.00] | **0.854** |
| **Number of orders of diazepam for injection (from another manufacture) prescribed for each patient (median [IQR])** | **DXPZSY** | 0.00 [0.00, 0.00] | 0.00 [0.00, 0.00] | 0.00 [0.00, 0.00] | **0.839** |
| **Number of orders of lorazepam tablets prescribed for each patient (median [IQR])** | **LLXZ** | 0.00 [0.00, 0.00] | 0.00 [0.00, 0.00] | 0.00 [0.00, 1.00] | **<0.001** |
| **Number of orders of clonazepam tablets prescribed for each patient (median [IQR])** | **LXXP** | 0.00 [0.00, 16.00] | 0.00 [0.00, 16.00] | 0.00 [0.00, 18.00] | **0.021** |
| **Number of orders of clonazepam for injection prescribed for each patient (median [IQR])** | **LXXPZSY** | 0.00 [0.00, 0.00] | 0.00 [0.00, 0.00] | 0.00 [0.00, 0.00] | **0.11** |
| **Number of orders of midazolam for injection prescribed for each patient (median [IQR])** | **MDZLZSY** | 0.00 [0.00, 0.00] | 0.00 [0.00, 0.00] | 0.00 [0.00, 0.00] | **0.026** |
| **Number of orders of tandospirone citrate capsules prescribed for each patient (median [IQR])** | **TDLTJL** | 0.00 [0.00, 0.00] | 0.00 [0.00, 0.00] | 0.00 [0.00, 0.00] | **<0.001** |
| **Number of orders of ezopiclone tablets prescribed for each patient (median [IQR])** | **YZPKL** | 0.00 [0.00, 0.00] | 0.00 [0.00, 0.00] | 0.00 [0.00, 0.00] | **<0.001** |
| **Sum of mood stabilizer orders prescribed for each patient (median [IQR])** | **MSB_SUM** | 0.00 [0.00, 0.00] | 0.00 [0.00, 0.00] | 0.00 [0.00, 0.00] | **0.005** |
| **Number of orders of sodium valproate sustained-release tablets prescribed for each patient (median [IQR])** | **BWSN** | 0.00 [0.00, 0.00] | 0.00 [0.00, 0.00] | 0.00 [0.00, 0.00] | **0.173** |
| **Number of orders of lamotrigine tablets prescribed for each patient (median [IQR])** | **LMSZ** | 0.00 [0.00, 0.00] | 0.00 [0.00, 0.00] | 0.00 [0.00, 0.00] | **0.001** |
| **Number of orders of topiramate capsules prescribed for each patient (median [IQR])** | **TBZ** | 0.00 [0.00, 0.00] | 0.00 [0.00, 0.00] | 0.00 [0.00, 0.00] | **0.167** |
| **Number of orders of lithium carbonate tablets prescribed for each patient (median [IQR])** | **TSZ** | 0.00 [0.00, 0.00] | 0.00 [0.00, 0.00] | 0.00 [0.00, 0.00] | **<0.001** |
| **Sum of anti-side effects drugs orders prescribed for each patient (median [IQR])** | **ASE_SUM** | 0.00 [0.00, 7.00] | 0.00 [0.00, 7.00] | 0.00 [0.00, 10.50] | **0.002** |
| **Number of orders of aspirin enteric-coated tablets prescribed for each patient (median [IQR])** | **ASPL** | 0.00 [0.00, 0.00] | 0.00 [0.00, 0.00] | 0.00 [0.00, 0.00] | **0.037** |
| **Number of orders of atorvastatin calcium tablets prescribed for each patient (median [IQR])** | **ATFTTG** | 0.00 [0.00, 0.00] | 0.00 [0.00, 0.00] | 0.00 [0.00, 0.00] | **0.018** |
| **Number of orders of benhexol hydrochloride tablets prescribed for each patient (median [IQR])** | **BHS** | 0.00 [0.00, 0.00] | 0.00 [0.00, 0.00] | 0.00 [0.00, 0.00] | **<0.001** |
| **Number of orders of bisacodyl enteric-coated tablets prescribed for each patient (median [IQR])** | **BSKD** | 0.00 [0.00, 0.00] | 0.00 [0.00, 0.00] | 0.00 [0.00, 0.00] | **<0.001** |
| **Number of orders of polyethylene glycol prescribed for each patient (median [IQR])** | **JYEC** | 0.00 [0.00, 0.00] | 0.00 [0.00, 0.00] | 0.00 [0.00, 0.00] | **0.189** |
| **Number of orders of glycerine enema prescribed for each patient (median [IQR])** | **KSL** | 0.00 [0.00, 0.00] | 0.00 [0.00, 0.00] | 0.00 [0.00, 0.00] | **0.074** |
| **Number of orders of maren maru prescribed for each patient (median [IQR])** | **MR** | 0.00 [0.00, 0.00] | 0.00 [0.00, 0.00] | 0.00 [0.00, 0.00] | **0.144** |
| **Number of orders of mosapride citrate tablets prescribed for each patient (median [IQR])** | **MSBL** | 0.00 [0.00, 0.00] | 0.00 [0.00, 0.00] | 0.00 [0.00, 0.00] | **0.384** |
| **Number of orders of zopiclone tartrate tablets prescribed for each patient (median [IQR])** | **HYP_ZZT** | 0.00 [0.00, 0.00] | 0.00 [0.00, 0.00] | 0.00 [0.00, 0.00] | **0.009** |
| **Sum of β receptor blocker orders prescribed for each patient (median [IQR])** | **OT_SUM** | 0.00 [0.00, 0.00] | 0.00 [0.00, 0.00] | 0.00 [0.00, 0.50] | **<0.001** |
| **Number of orders of metoprolol succinate sustained-release tablets prescribed for each patient (median [IQR])** | **HPSMTLE** | 0.00 [0.00, 0.00] | 0.00 [0.00, 0.00] | 0.00 [0.00, 0.00] | **0.364** |
| **Number of orders of metoprolol tartrate tablets prescribed for each patient (median [IQR])** | **JSSMTLE** | 0.00 [0.00, 0.00] | 0.00 [0.00, 0.00] | 0.00 [0.00, 0.00] | **0.759** |
| **Number of orders of propranolol hydrochloride tablets prescribed for each patient (median [IQR])** | **PNLE** | 0.00 [0.00, 0.00] | 0.00 [0.00, 0.00] | 0.00 [0.00, 0.00] | **<0.001** |
| **Sum of hormonal drugs orders prescribed for each patient (median [IQR])** | **T3_SUM** | 0.00 [0.00, 0.00] | 0.00 [0.00, 0.00] | 0.00 [0.00, 0.00] | **0.035** |
| **Sum of Chinese patent medicines orders prescribed for each patient (median [IQR])** | **CM_SUM** | 0.00 [0.00, 0.00] | 0.00 [0.00, 0.00] | 0.00 [0.00, 0.00] | **<0.001** |
| **Sum of physiotherapy orders prescribed for each patient (median [IQR])** | **PHY_SUM** | 0.00 [0.00, 28.00] | 0.00 [0.00, 28.00] | 8.00 [0.00, 42.00] | **<0.001** |
| **Number of orders of multi-parameter biofeedback therapy prescribed for each patient (median [IQR])** | **MPBT** | 0.00 [0.00, 0.00] | 0.00 [0.00, 0.00] | 0.00 [0.00, 0.00] | **0.216** |
| **Number of orders of modified electroconvulsive therapy prescribed for each patient (median [IQR])** | **ME** | 0.00 [0.00, 0.00] | 0.00 [0.00, 0.00] | 0.00 [0.00, 0.00] | **<0.001** |
| **Number of orders of transcranial magnetic stimulation therapy prescribed for each patient (median [IQR])** | **TMS** | 0.00 [0.00, 0.00] | 0.00 [0.00, 0.00] | 0.00 [0.00, 0.00] | **0.166** |
| **Number of orders of electroencephalographic (EEG) biofeedback therapy prescribed for each patient (median [IQR])** | **EEG** | 0.00 [0.00, 24.00] | 0.00 [0.00, 24.00] | 0.00 [0.00, 28.00] | **<0.001** |
| **Sum of psychotherapy orders prescribed for each patient (median [IQR])** | **PSY_SUM** | 1.00 [0.00, 4.00] | 1.00 [0.00, 4.00] | 1.00 [0.00, 4.00] | **0.022** |

**2.2 Predictive performance of ML models**

To avoid favorable train-test splits in the data, performance estimates from four different test sets for each prediction-modeling cohort were calculated and presented in the following Table S7 and Figure S1-S5.

Table S7: Performance of the prediction models of 30-day, 60-day, 90-day, 180-day, and 365-day psychiatric readmission.

| **Performance of testing data** | **Days of follow-up** | **Multiple train-test splits** | **AUC (90% CI)** | **P-value**  **(Determined by DeLong’s test)** | **Threshold**  **(Determined by Youden index)** | **Sensitivity** | **Specificity** | **PPV** | **NPV** |
| --- | --- | --- | --- | --- | --- | --- | --- | --- | --- |
| ***SVM*** | 30 days | The initial split | 0.802 (0.745-0.858) | / | 0.500 | 0.754 | 0.770 | 0.096 | 0.990 |
|  |  | The second split | 0.690 (0.630-0.751) | 0.008 | 0.493 | 0.543 | 0.737 | 0.069 | 0.978 |
|  |  | The third split | 0.726 (0.665-0.786) | 0.067 | 0.547 | 0.580 | 0.771 | 0.102 | 0.980 |
|  |  | The fourth split | 0.710 (0.631-0.790) | 0.066 | 0.492 | 0.674 | 0.685 | 0.051 | 0.989 |
|  | 60 days | The initial split | 0.766 (0.710-0.822) | / | 0.500 | 0.613 | 0.833 | 0.140 | 0.980 |
|  |  | The second split | 0694 (0.633-0.755) | 0.081 | 0.535 | 0.595 | 0.746 | 0.092 | 0.977 |
|  |  | The third split | 0.707 (0.656-0.759) | 0.119 | 0.519 | 0.685 | 0.680 | 0.107 | 0.975 |
|  |  | The fourth split | 0.714 (0.649-0.778) | 0.216 | 0.566 | 0.533 | 0.792 | 0.096 | 0.976 |
|  | 90 days | The initial split | 0.763 (0.712-0.814) | / | 0.504 | 0.647 | 0.760 | 0.132 | 0.975 |
|  |  | The second split | 0.710 (0.658-0.762) | 0.155 | 0.560 | 0.546 | 0.796 | 0.119 | 0.972 |
|  |  | The third split | 0.692 (0.641-0.743) | 0.054 | 0.587 | 0.412 | 0.866 | 0.166 | 0.958 |
|  |  | The fourth split | 0.702 (0.647-0.757) | 0.106 | 0.487 | 0.753 | 0.578 | 0.087 | 0.977 |
|  | 180 days | The initial split | 0.712 (0.655-0.768) | / | 0.498 | 0.493 | 0.814 | 0.184 | 0.950 |
|  |  | The second split | 0.683 (0.640-0.726) | 0.437 | 0.509 | 0.697 | 0.577 | 0.110 | 0.960 |
|  |  | The third split | 0.706 (0.664-0.747) | 0.867 | 0.474 | 0.757 | 0.561 | 0.130 | 0.964 |
|  |  | The fourth split | 0.686 (0.639-0.733) | 0.497 | 0.586 | 0.496 | 0.791 | 0.116 | 0.969 |
|  | 365 days | The initial split | 0.699 (0.664-0.735) | / | 0.422 | 0.827 | 0.476 | 0.145 | 0.962 |
|  |  | The second split | 0.724 (0.690-0.75) | 0.322 | 0.459 | 0.78 | 0.58 | 0.200 | 0.951 |
|  |  | The third split | 0.688 (0.648-0.728) | 0.684 | 0.562 | 0.512 | 0.767 | 0.218 | 0.925 |
|  |  | The fourth split | 0.634 (0.593-0.675) | 0.018 | 0.443 | 0.813 | 0.407 | 0.147 | 0.945 |
| ***Xgboost*** | 30 days | The initial split | 0.792 (0.735-0.850) | / | 0.513 | 0.738 | 0.727 | 0.080 | 0.988 |
|  |  | The second split | 0.774 (0.715-0.834) | 0.673 | 0.517 | 0.714 | 0.739 | 0.089 | 0.986 |
|  |  | The third split | 0.768 (0.706-0.830) | 0.576 | 0.639 | 0.609 | 0.845 | 0.117 | 0.983 |
|  |  | The fourth split | 0.781 (0.712-0.851) | 0.811 | 0.379 | 0.907 | 0.517 | 0.061 | 0.986 |
|  | 60 days | The initial split | 0.771 (0.719-0.823) | / | 0.435 | 0.867 | 0.560 | 0.081 | 0.990 |
|  |  | The second split | 0.752 (0.703-0.802) | 0.614 | 0.554 | 0.667 | 0.786 | 0.119 | 0.982 |
|  |  | The third split | 0.752 (0.703-0.802) | 0.614 | 0.452 | 0.820 | 0.596 | 0.102 | 0.983 |
|  |  | The fourth split | 0.748 (0.688-0.808) | 0.566 | 0.456 | 0.767 | 0.598 | 0.073 | 0.984 |
|  | 90 days | The initial split | 0.773 (0.723-0.824) | / | 0.574 | 0.635 | 0.801 | 0.152 | 0.975 |
|  |  | The second split | 0.737 (0.684-0.790) | 0.334 | 0.611 | 0.505 | 0.872 | 0.166 | 0.972 |
|  |  | The third split | 0.741 (0.691-0.791) | 0.377 | 0.510 | 0.676 | 0.704 | 0.129 | 0.971 |
|  |  | The fourth split | 0.744 (0.689-0.799) | 0.448 | 0.478 | 0.753 | 0.629 | 0.098 | 0.979 |
|  | 180 days | The initial split | 0.702 (0.644-0.760) | / | 0.451 | 0.707 | 0.593 | 0.129 | 0.960 |
|  |  | The second split | 0.705 (0.662-0.749) | 0.918 | 0.497 | 0.669 | 0.629 | 0.154 | 0.955 |
|  |  | The third split | 0.735 (0.695-0.775) | 0.355 | 0.499 | 0.684 | 0.661 | 0.134 | 0.971 |
|  |  | The fourth split | 0.702 (0.658-0.747) | 0.984 | 0.461 | 0.798 | 0.515 | 0.109 | 0.978 |
|  | 365 days | The initial split | 0.703 (0.667-0.740) | / | 0.495 | 0.658 | 0.639 | 0.163 | 0.962 |
|  |  | The second split | 0.743 (0.709-0.778) | 0.117 | 0.499 | 0.69 | 0.675 | 0.223 | 0.942 |
|  |  | The third split | 0.708 (0.671-0.746) | 0.845 | 0.485 | 0.741 | 0.597 | 0.189 | 0.948 |
|  |  | The fourth split | 0.669 (0.629-0.708) | 0.208 | 0.491 | 0.795 | 0.473 | 0.160 | 0.948 |
| ***Logistic regression*** | 30 days | The initial split | 0.697 (0.630-0.764) | / | 0.602 | 0.557 | 0.758 | 0.069 | 0.981 |
|  |  | The second split | 0.714 (0.659-0.769) | 0.702 | 0.388 | 0.871 | 0.462 | 0.055 | 0.990 |
|  |  | The third split | 0.731 (0.667-0.795) | 0.473 | 0.572 | 0.638 | 0.752 | 0.087 | 0.977 |
|  |  | The fourth split | 0.692 (0.615-0.769) | 0.923 | 0.515 | 0.698 | 0.649 | 0.051 | 0.984 |
|  | 60 days | The initial split | 0.701 (0.641-0.760) | / | 0.614 | 0.480 | 0.830 | 0.111 | 0.973 |
|  |  | The second split | 0.692 (0.637-0.748) | 0.837 | 0.588 | 0.536 | 0.756 | 0.087 | 0.974 |
|  |  | The third split | 0.675 (0.619-0.730) | 0.530 | 0.456 | 0.730 | 0.561 | 0.085 | 0.974 |
|  |  | The fourth split | 0.698 (0.631-0.765) | 0.948 | 0.681 | 0.45 | 0.845 | 0.107 | 0.974 |
|  | 90 days | The initial split | 0.732 (0.676-0.788) | / | 0.560 | 0.635 | 0.758 | 0.129 | 0.974 |
|  |  | The second split | 0.708 (0.656-0.759) | 0.534 | 0.512 | 0.670 | 0.659 | 0.090 | 0.975 |
|  |  | The third split | 0.684 (0.629-0.739) | 0.235 | 0.530 | 0.578 | 0.720 | 0.118 | 0.964 |
|  |  | The fourth split | 0.685 (0.627-0.744) | 0.262 | 0.573 | 0.571 | 0.748 | 0.108 | 0.970 |
|  | 180 days | The initial split | 0.669 (0.615-0.723) | / | 0.396 | 0.813 | 0.479 | 0.117 | 0.968 |
|  |  | The second split | 0.664 (0.620-0.708) | 0.885 | 0.527 | 0.570 | 0.665 | 0.11 | 0.972 |
|  |  | The third split | 0.697 (0.653-0.741) | 0.430 | 0.406 | 0.853 | 0.457 | 0.114 | 0.983 |
|  |  | The fourth split | 0.672 (0.622-0.722) | 0.929 | 0.592 | 0.538 | 0.743 | 0.104 | 0.966 |
|  | 365 days | The initial split | 0.667 (0.629-0.704) | / | 0.394 | 0.796 | 0.443 | 0.133 | 0.953 |
|  |  | The second split | 0.731 (0.697-0.765) | 0.012 | 0.440 | 0.82 | 0.527 | 0.190 | 0.960 |
|  |  | The third split | 0.676 (0.635-0.717) | 0.741 | 0.455 | 0.735 | 0.529 | 0.165 | 0.940 |
|  |  | The fourth split | 0.626 (0.585-0.667) | 0.154 | 0.486 | 0.687 | 0.508 | 0.149 | 0.928 |
| ***Random forest*** | 30 days | The initial split | 0.814 (0.758-0.870) | / | 0.515 | 0.738 | 0.784 | 0.099 | 0.989 |
|  |  | The second split | 0.766 (0.706-0.826) | 0.254 | 0.536 | 0.629 | 0.793 | 0.098 | 0.983 |
|  |  | The third split | 0.780 (0.723-0.837) | 0.411 | 0.614 | 0.536 | 0.889 | 0.089 | 0.989 |
|  |  | The fourth split | 0.765 (0.696-0.835) | 0.290 | 0.503 | 0.674 | 0.738 | 0.054 | 0.991 |
|  | 60 days | The initial split | 0.780 (0.728-0.833) | / | 0.506 | 0.733 | 0.720 | 0.104 | 0.984 |
|  |  | The second split | 0.773 (0.722-0.824) | 0.841 | 0.487 | 0.774 | 0.671 | 0.093 | 0.986 |
|  |  | The third split | 0.766 (0.715-0.817) | 0.706 | 0.490 | 0.798 | 0.630 | 0.104 | 0.985 |
|  |  | The fourth split | 0.736 (0.675-0.798) | 0.285 | 0.458 | 0.800 | 0.545 | 0.068 | 0.985 |
|  | 90 days | The initial split | 0.798 (0.750-0.846) | / | 0.530 | 0.765 | 0.754 | 0.149 | 0.983 |
|  |  | The second split | 0.749 (0.696-0.801) | 0.172 | 0.542 | 0.619 | 0.777 | 0.123 | 0.976 |
|  |  | The third split | 0.758 (0.711-0.804) | 0.234 | 0.522 | 0.676 | 0.717 | 0.134 | 0.972 |
|  |  | The fourth split | 0.737 (0.680-0.794) | 0.106 | 0.492 | 0.753 | 0.636 | 0.100 | 0.980 |
|  | 180 days | The initial split | 0.740 (0.687-0.794) | / | 0.449 | 0.827 | 0.562 | 0.138 | 0.974 |
|  |  | The second split | 0.710 (0.667-0.753) | 0.382 | 0.527 | 0.606 | 0.711 | 0.144 | 0.961 |
|  |  | The third split | 0.766 (0.727-0.806) | 0.442 | 0.485 | 0.809 | 0.607 | 0.145 | 0.955 |
|  |  | The fourth split | 0.698 (0.652-0.743) | 0.235 | 0.484 | 0.723 | 0.573 | 0.116 | 0.972 |
|  | 365 days | The initial split | 0.711 (0.676-0.747) | / | 0.533 | 0.592 | 0.720 | 0.185 | 0.943 |
|  |  | The second split | 0.729 (0.694-0.764) | 0.492 | 0.543 | 0.585 | 0.742 | 0.234 | 0.930 |
|  |  | The third split | 0.699 (0.659-0.738) | 0.639 | 0.522 | 0.653 | 0.671 | 0.192 | 0.935 |
|  |  | The fourth split | 0.640 (0.599-0681) | 0.011 | 0.448 | 0.892 | 0.313 | 0.140 | 0.958 |

Figure S**1: ROC curves for the other three train-test splits of the 30-day cohort**


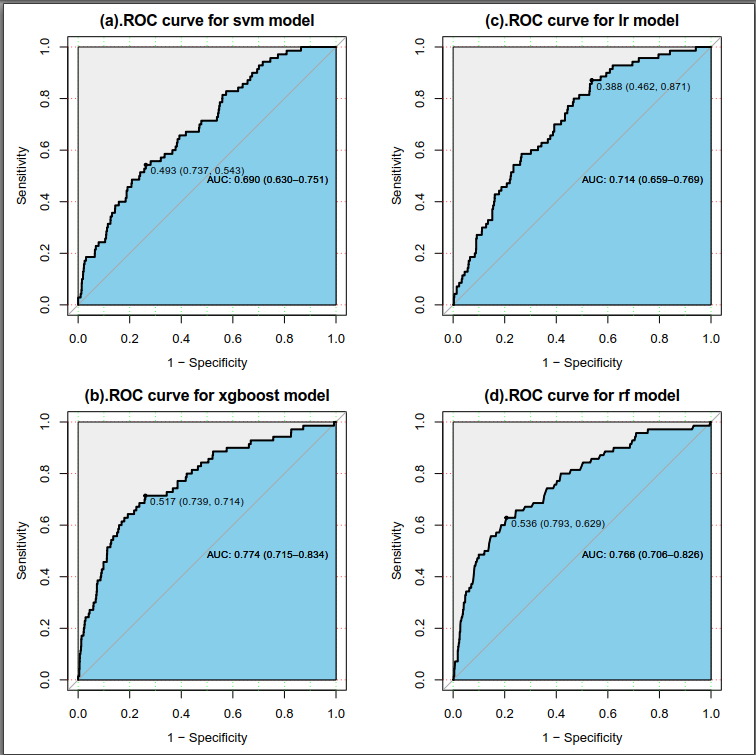

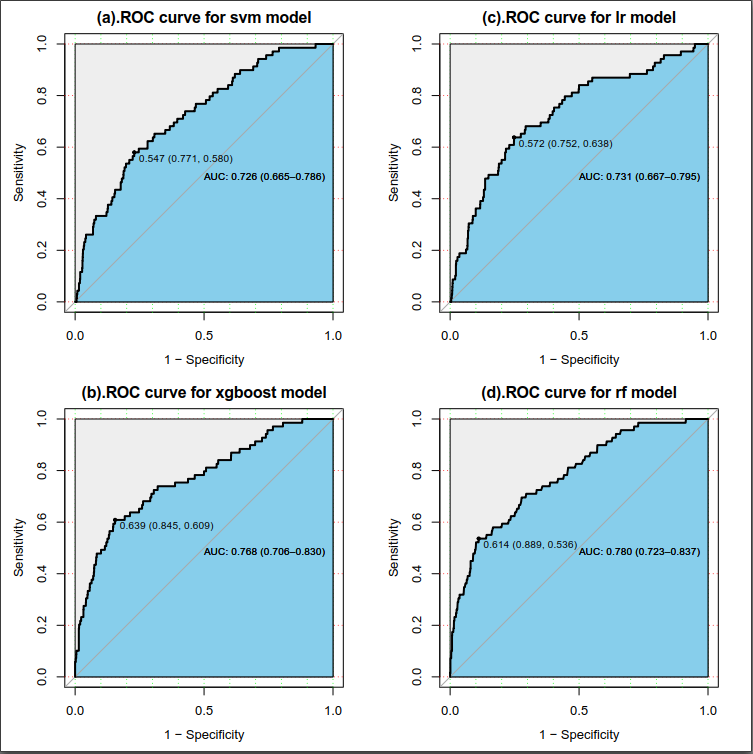

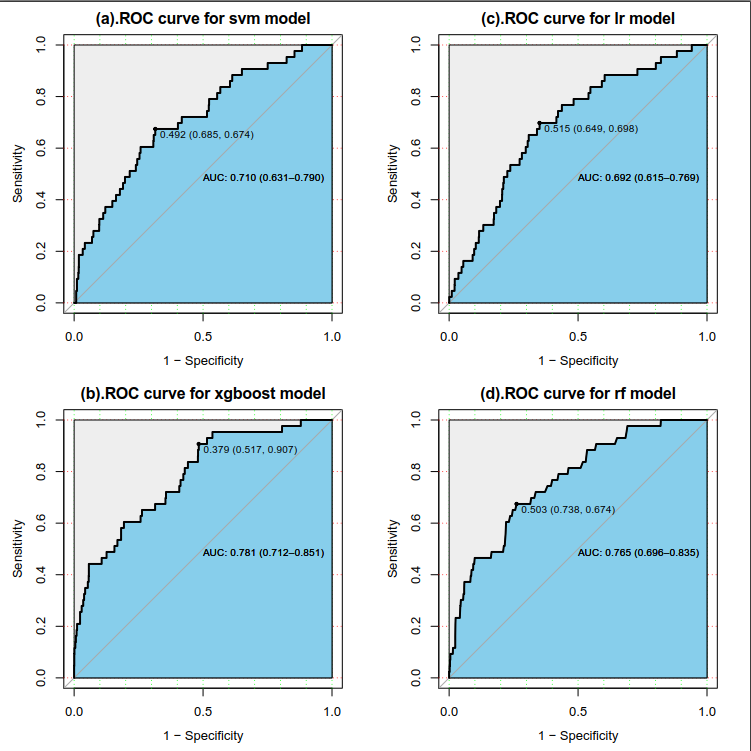


**(A) The second split (B) The third split (C) The fourth split**

Figure S**2: ROC curves for the other three train-test splits of the 60-day cohort**


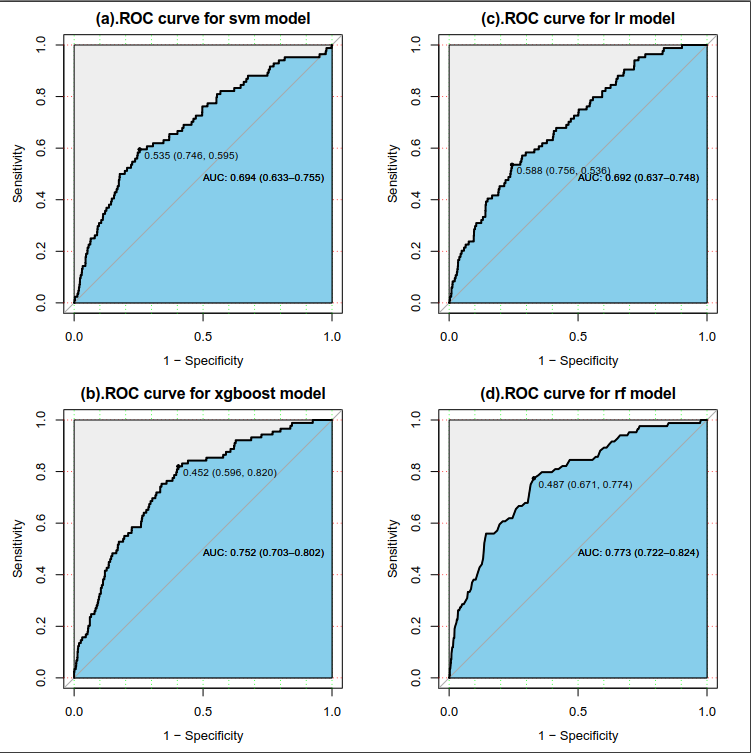

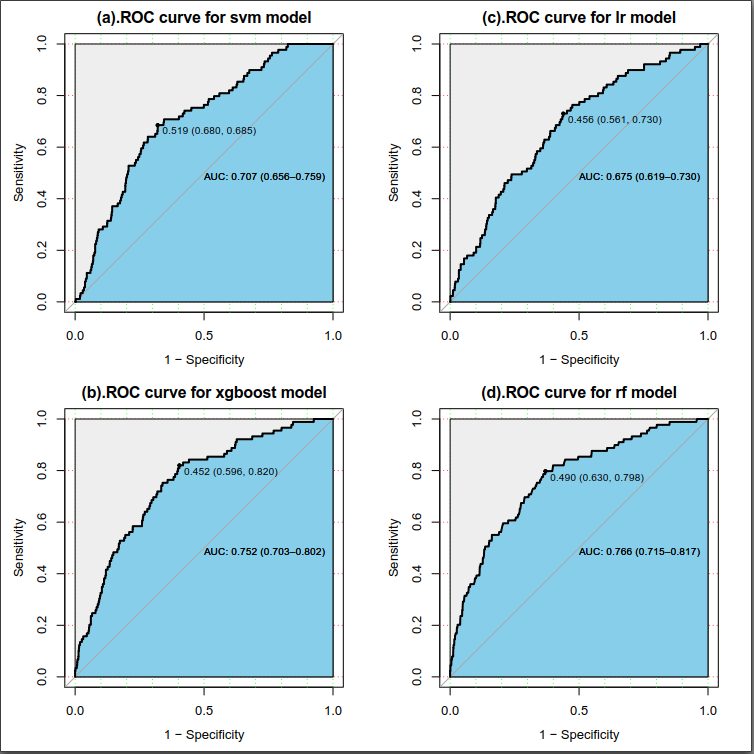

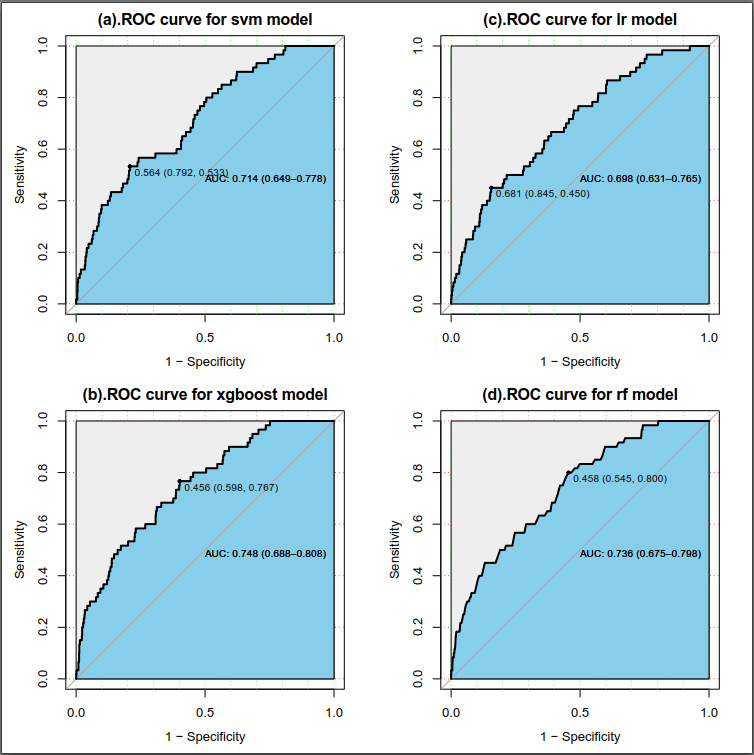


**(A) The second split (B) The third split (C) The fourth split**

Figure S**3: ROC curves for the other three train-test splits of the 90-day cohort**


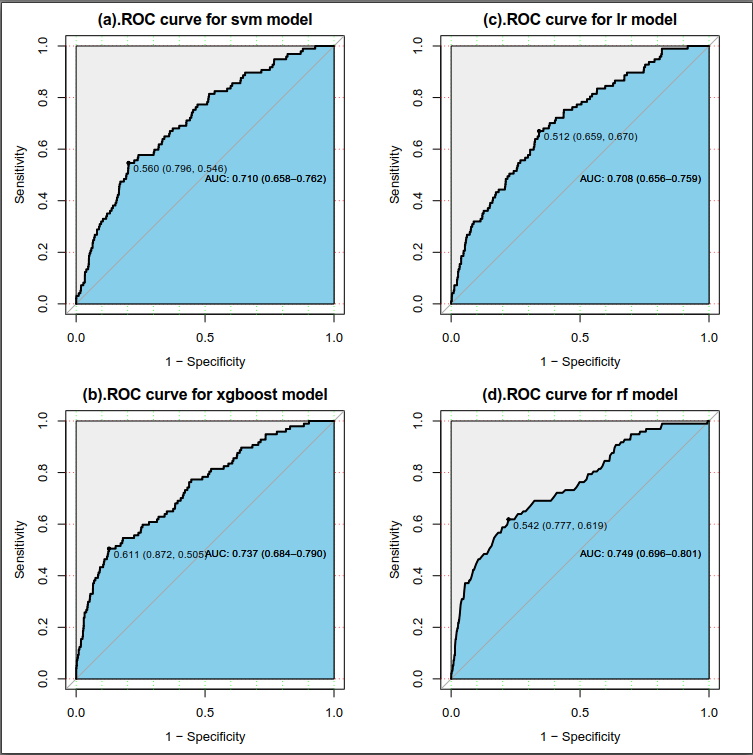

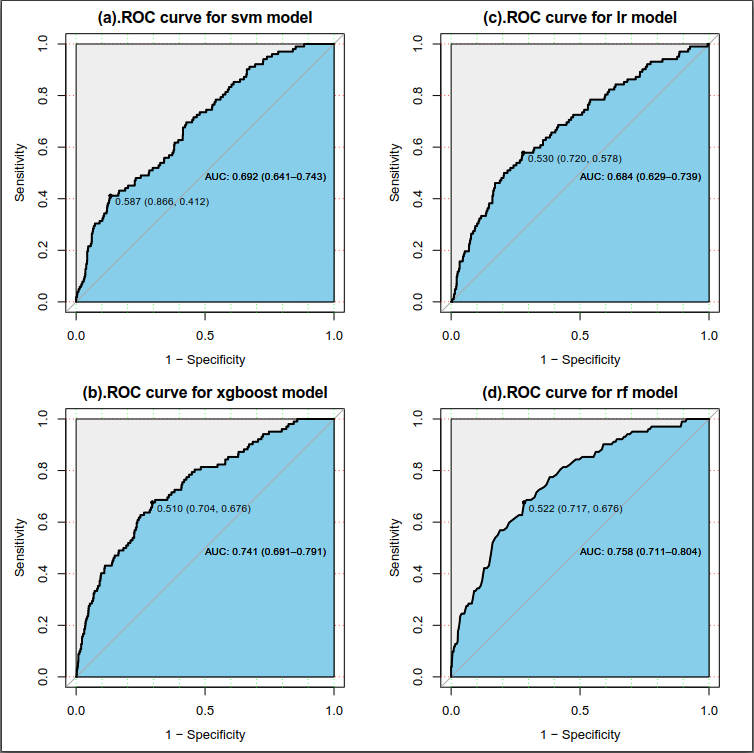

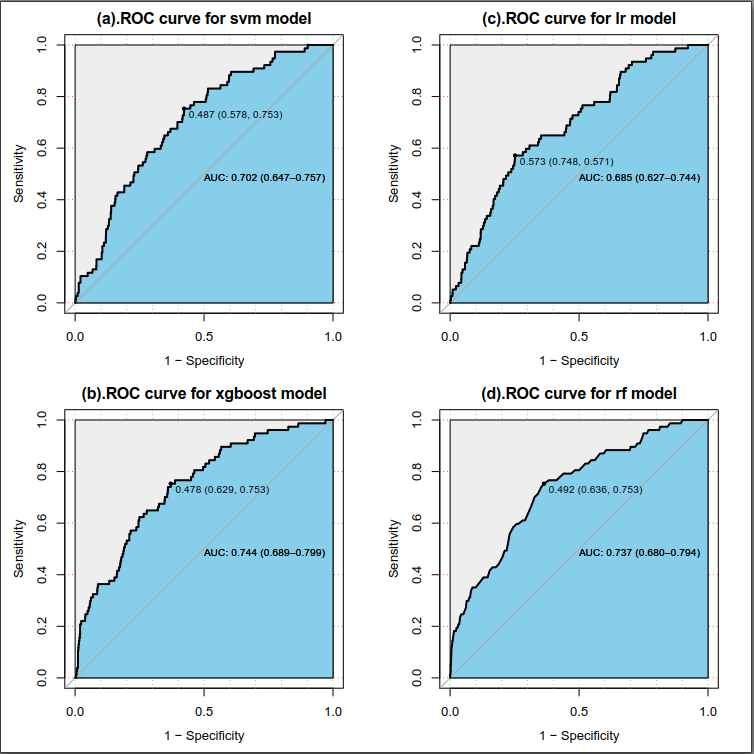


**(A) The second split (B) The third split (C) The fourth split**

Figure S**4: ROC curves for the other three train-test splits of the 180-day cohort**


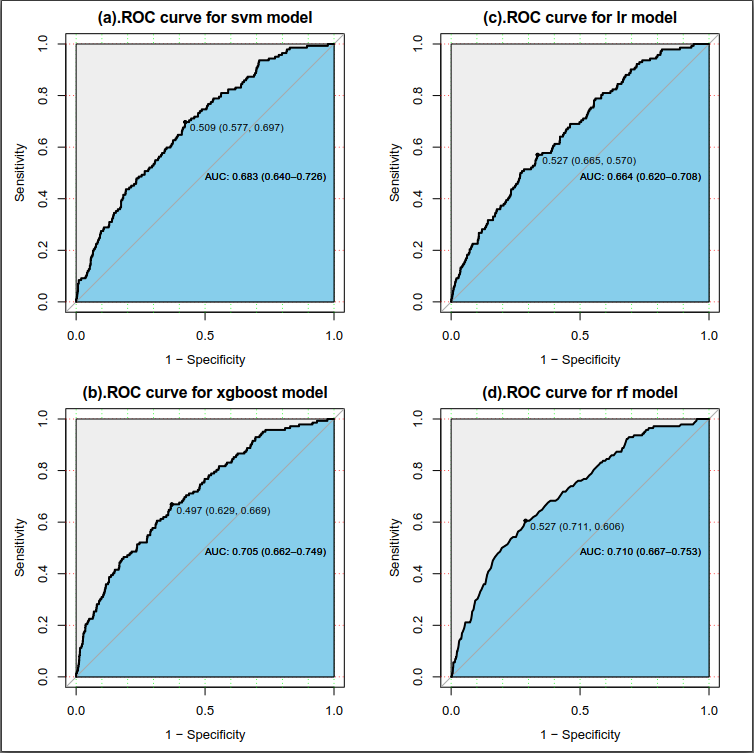

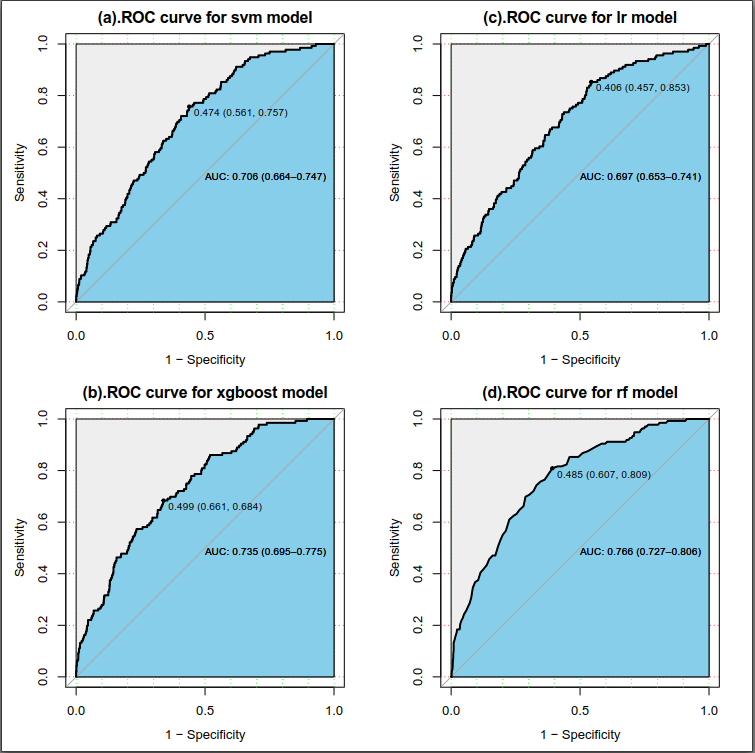

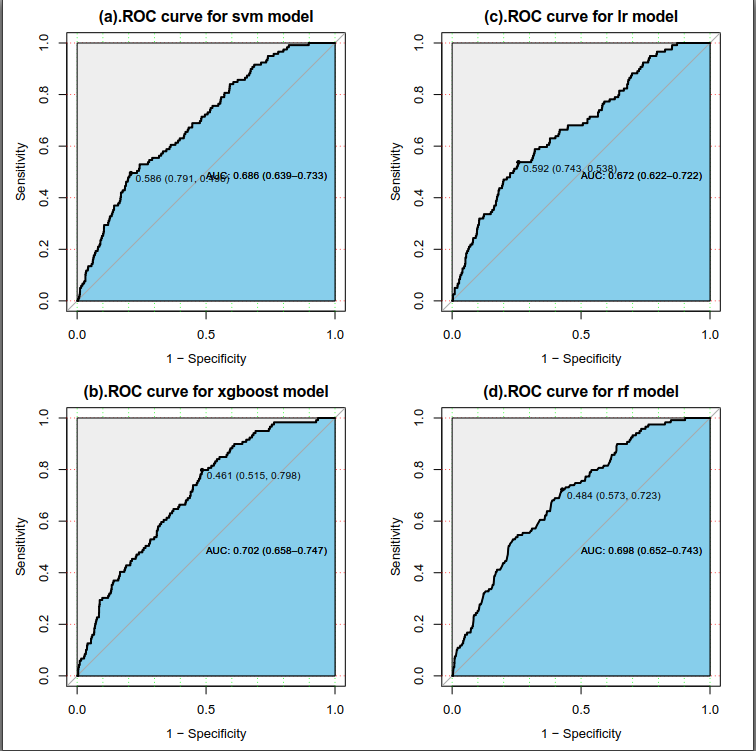


**(A) The second split (B) The third split (C) The fourth split**

Figure S**5: ROC curves for the other three train-test splits of the 365-day cohort**


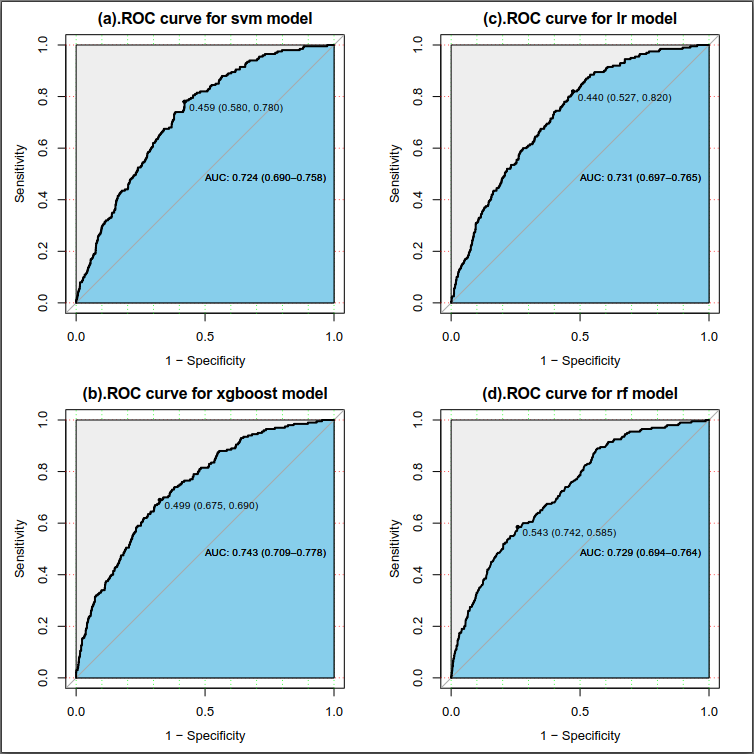

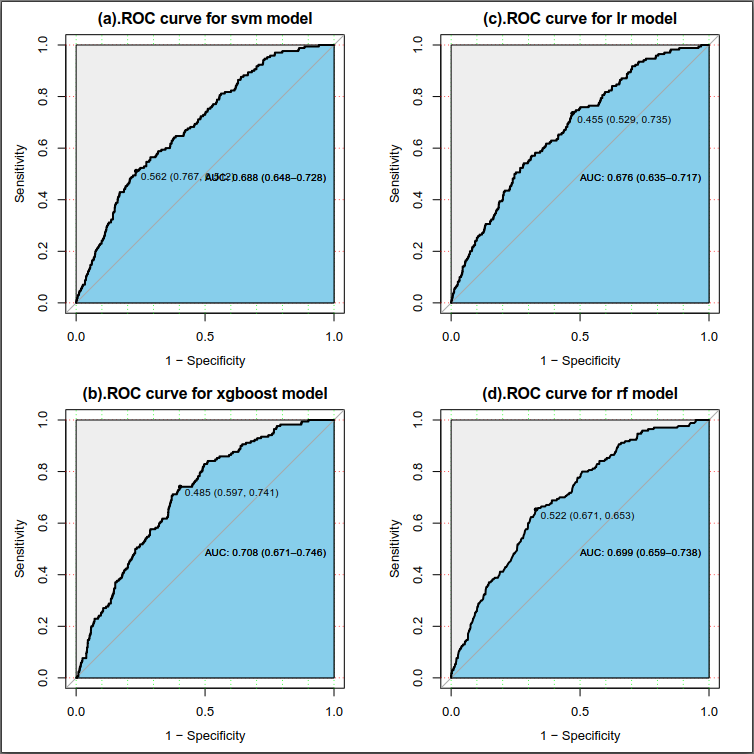

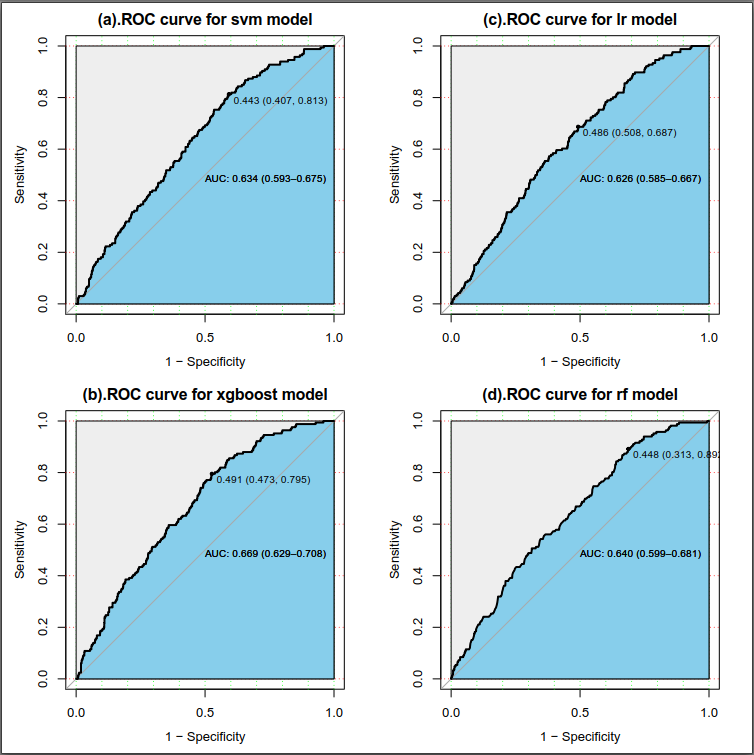


**(A) The second split (B) The third split (C) The fourth split**

**2.3 Important features**

When considering the relative importance of all features on the predictions for the holdout test dataset at individual patient level, results of the 60-day, 90-day, 180-day, and 365-day cohorts were shown in Figure S6, Figure S7, Figure S8, and Figure S9.


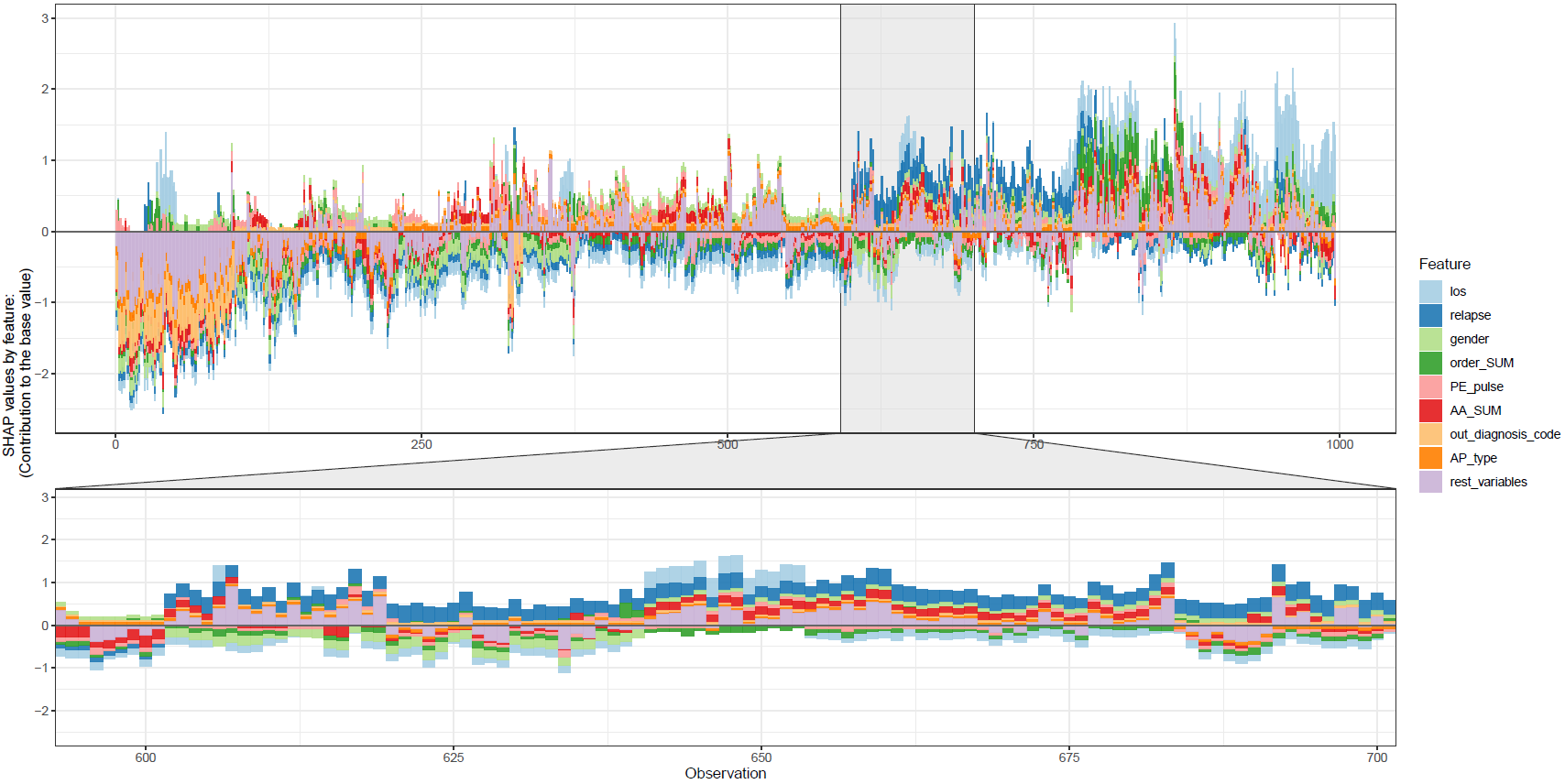


Figure S6 Force plot of all features at individual patient level for the holdout test dataset of the 60-day cohort


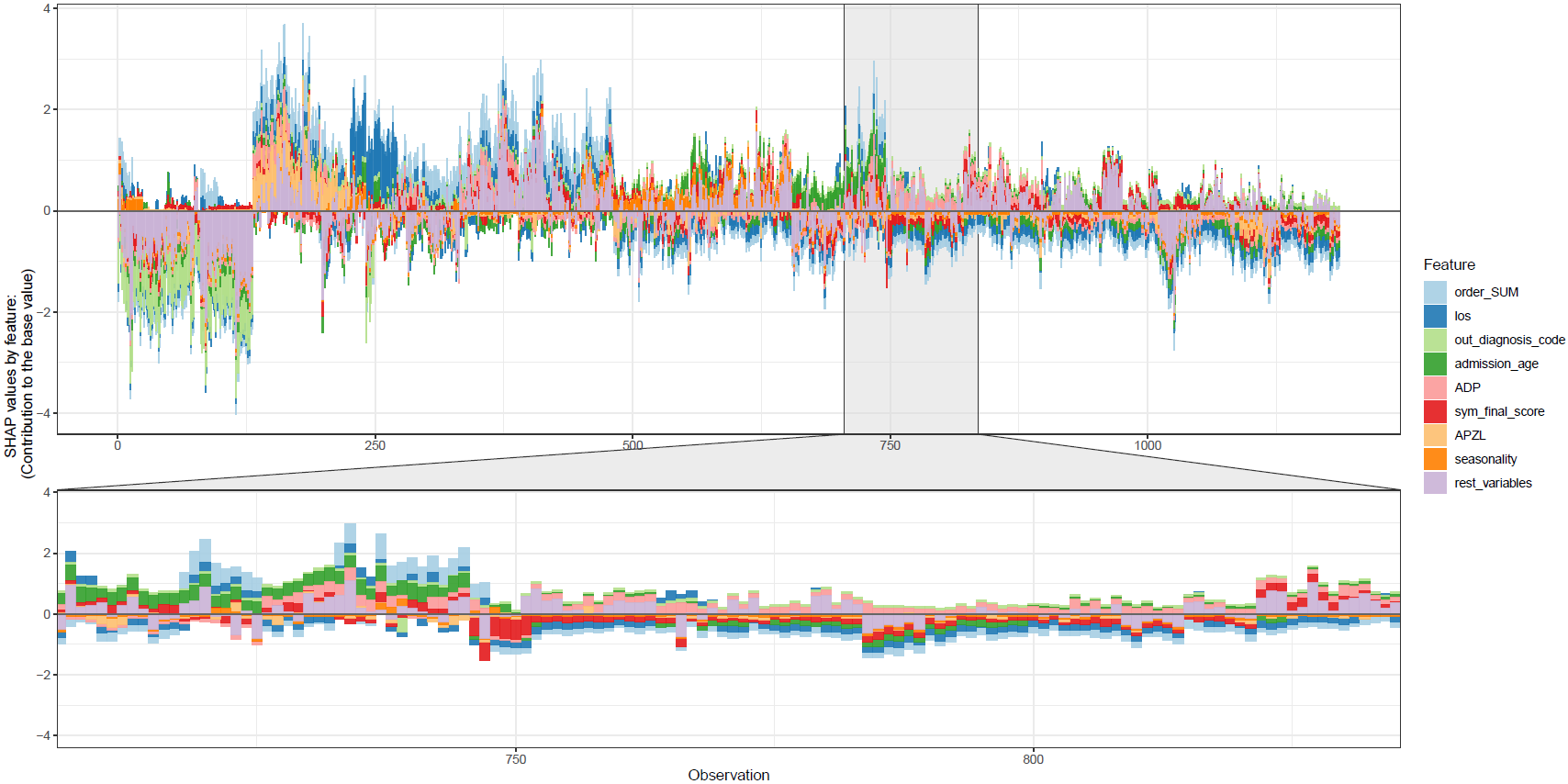


Figure S7 Force plot of all features at individual patient level for the holdout test dataset of the 90-day cohort


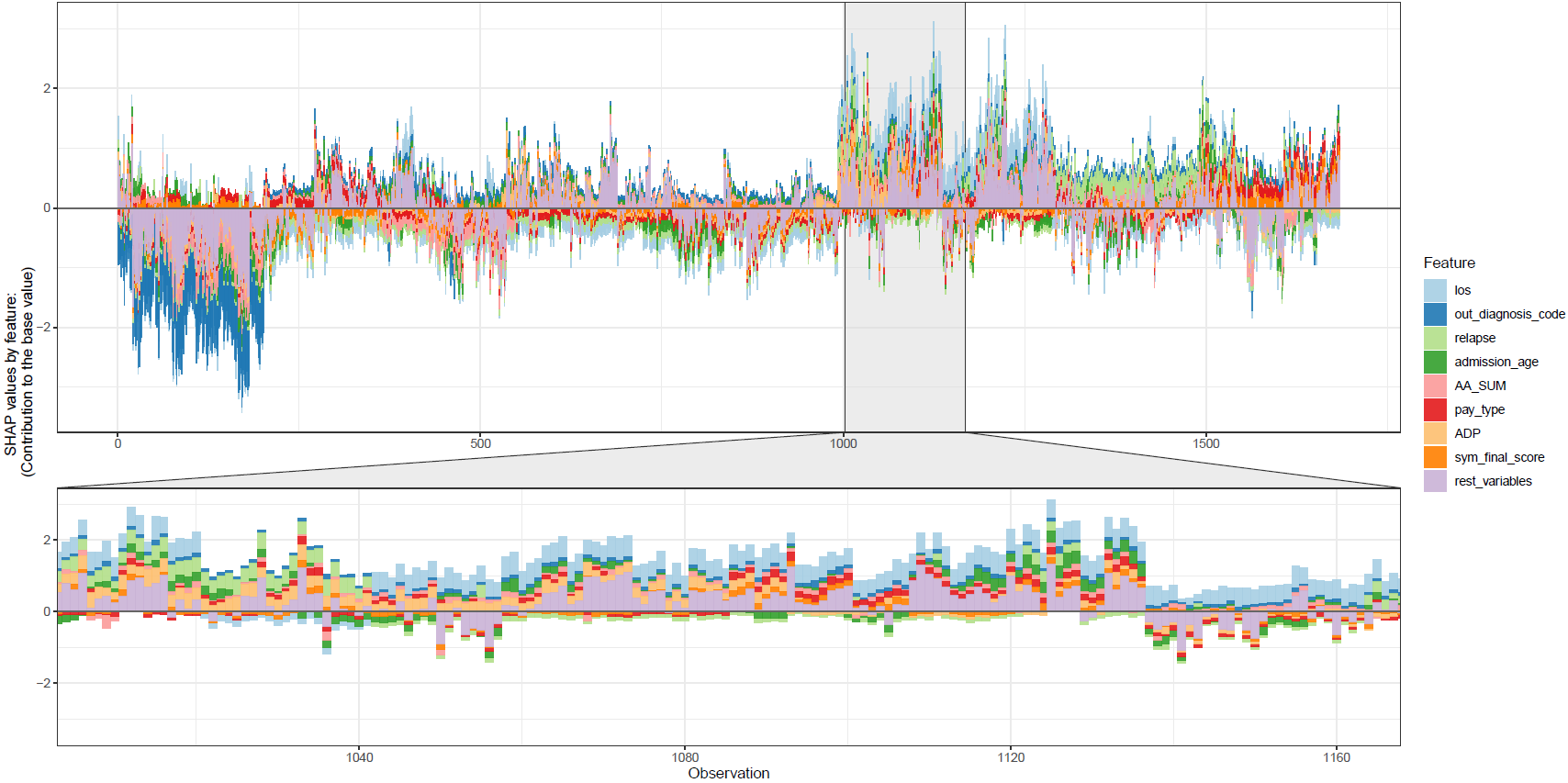


Figure S8 Force plot of all features at individual patient level for the holdout test dataset of the 180-day cohort


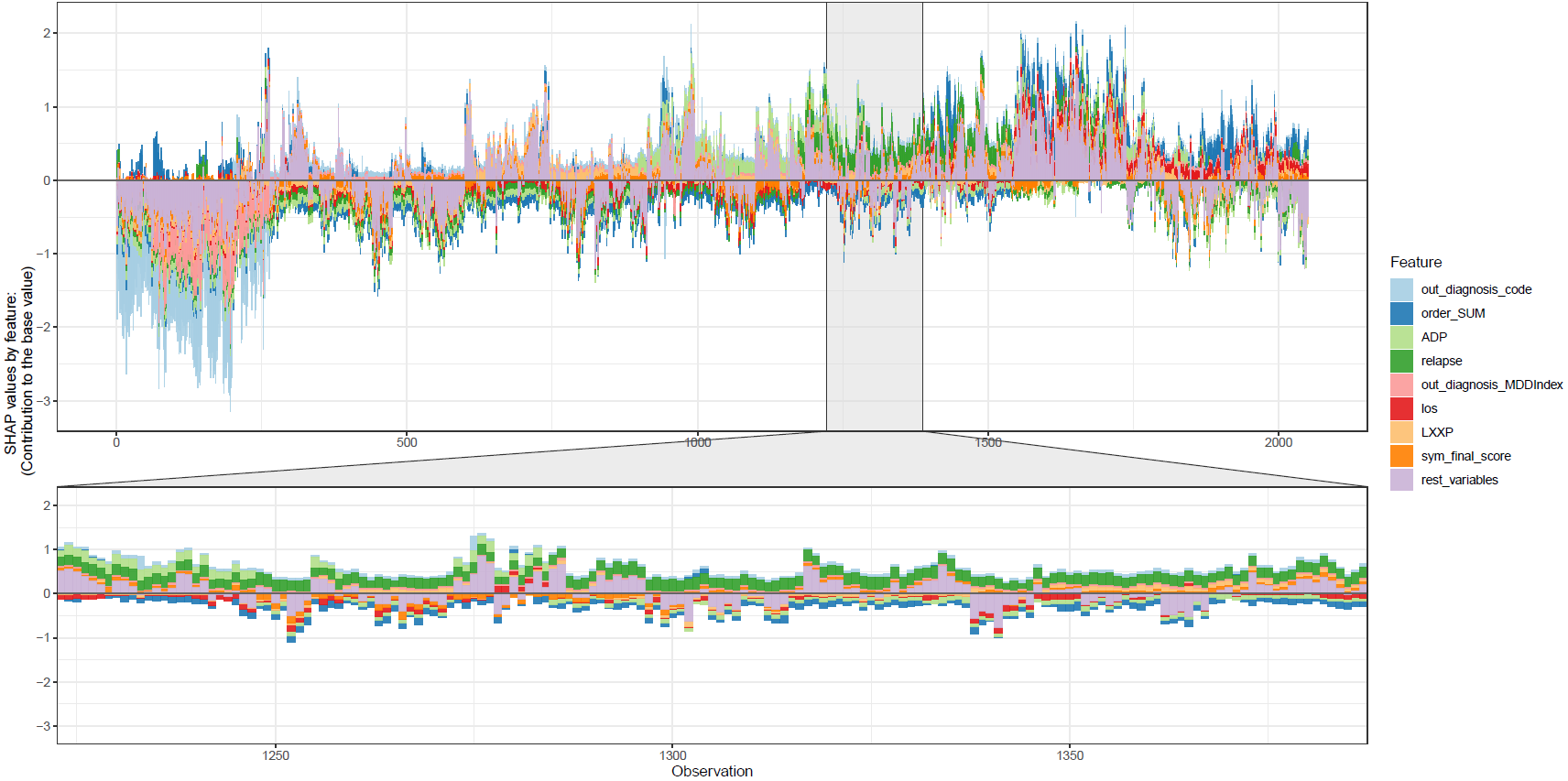


Figure S9 Force plot of all features at individual patient level for the holdout test dataset of the 365-day cohort

**2.4 Feature interactions**

When considering the graphical explanation of how 24 top important features interact, results of the 60-day, 90-day, 180-day, and 365-day cohorts were shown in Figure S10, Figure S11, Figure S12, and Figure S13.


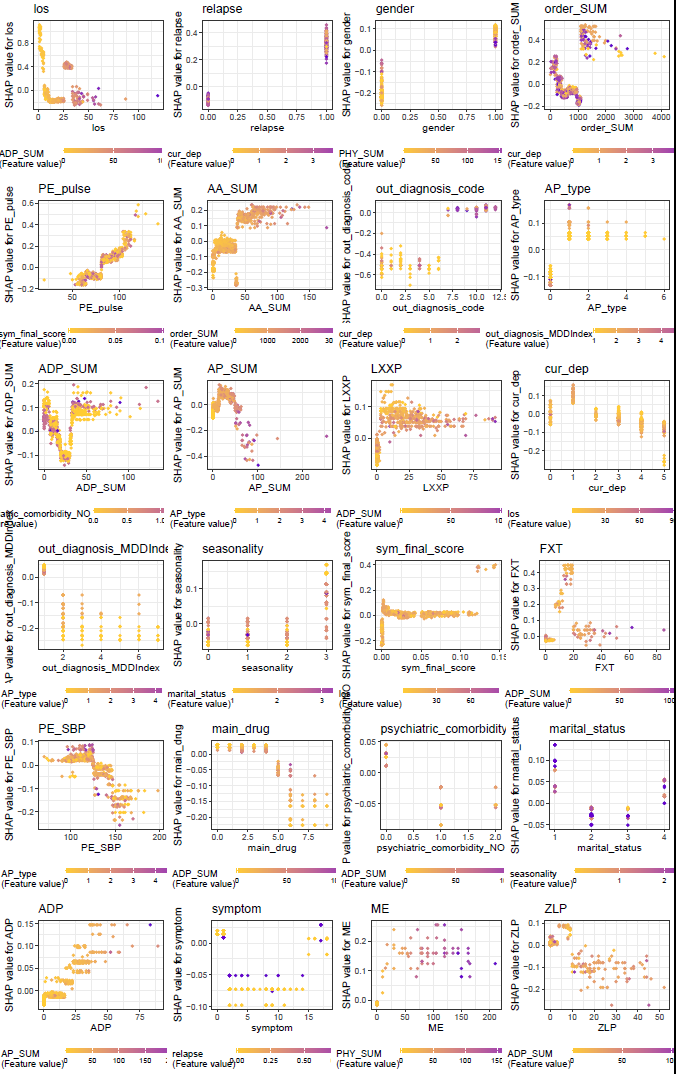


Figure S10 Feature interactions of 24 top important features of the 60-day cohort


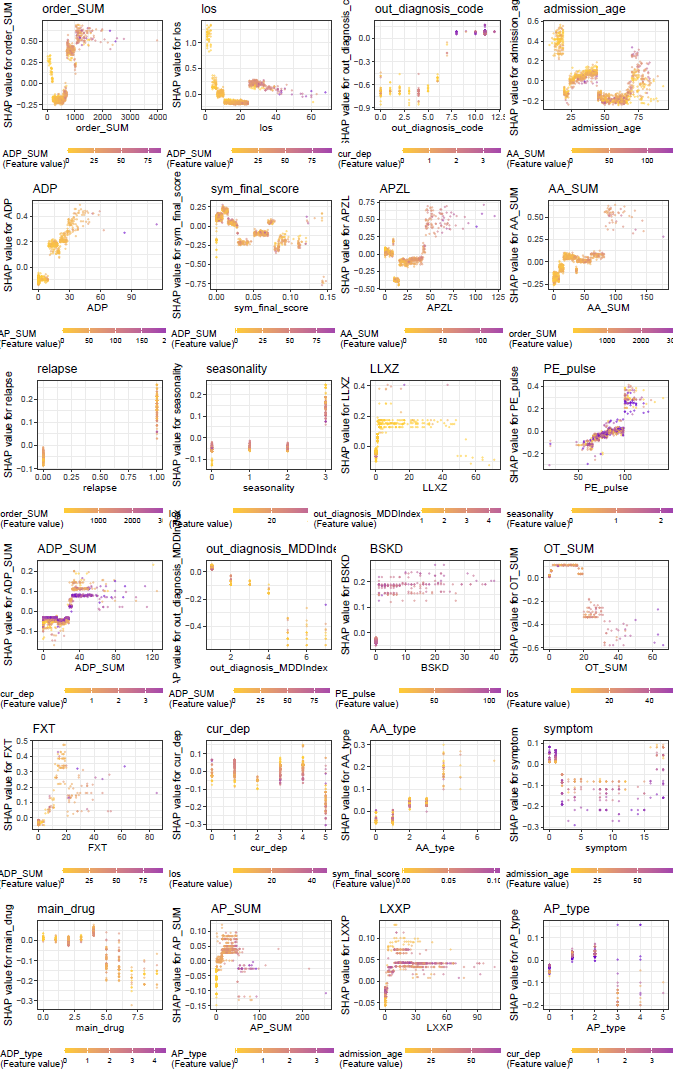


Figure S11 Feature interactions of 24 top important features of the 90-day cohort


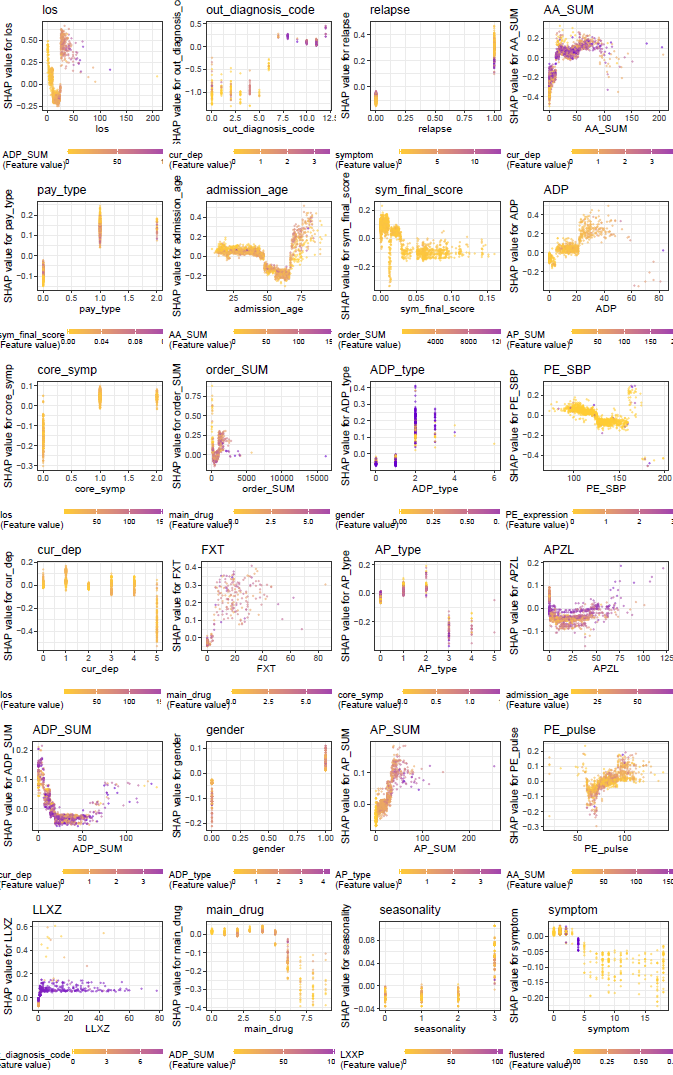


Figure S12 Feature interactions of 24 top important features of the 180-day cohort


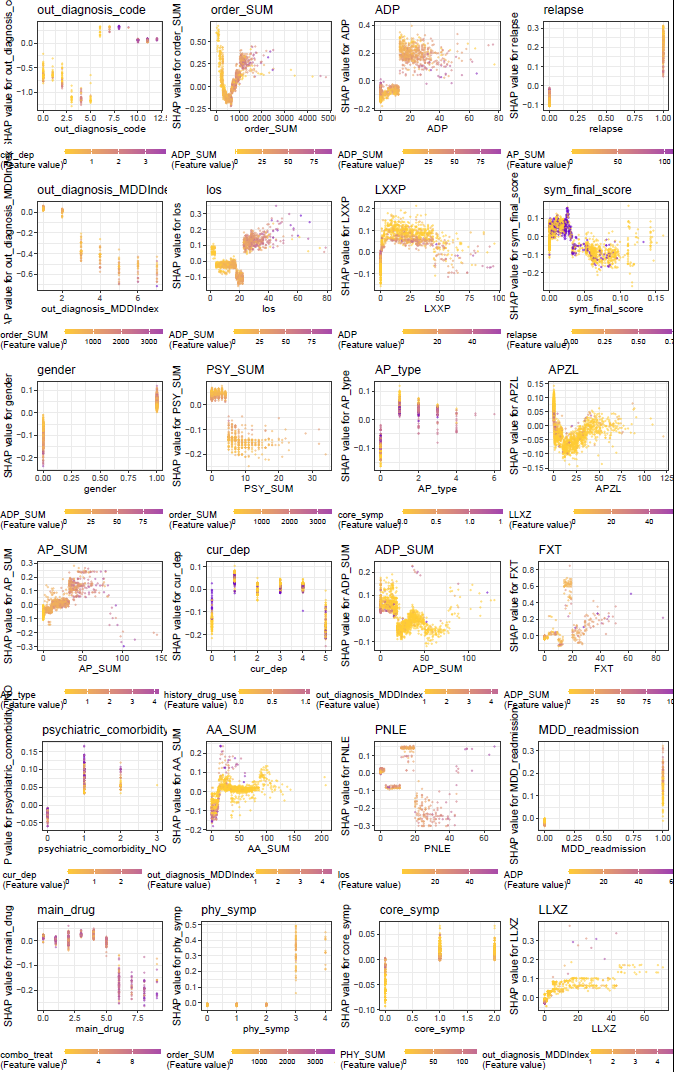


Figure S13 Feature interactions of 24 top important features of the 365-day cohort

**2.5 Individualized comparison on readmission and non-readmission**

Patients who are readmitted within 30 days are probably quite different from patients who are readmitted after 30 days. We also compared the difference between individuals readmitted and non-readmitted within a particular follow-up time (see the following Figures for example).


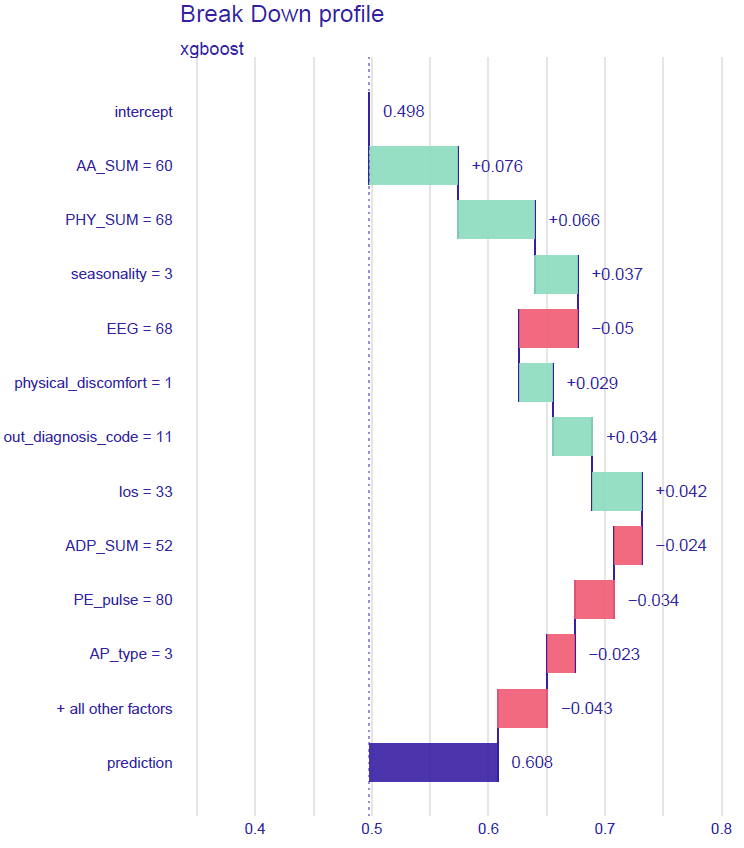

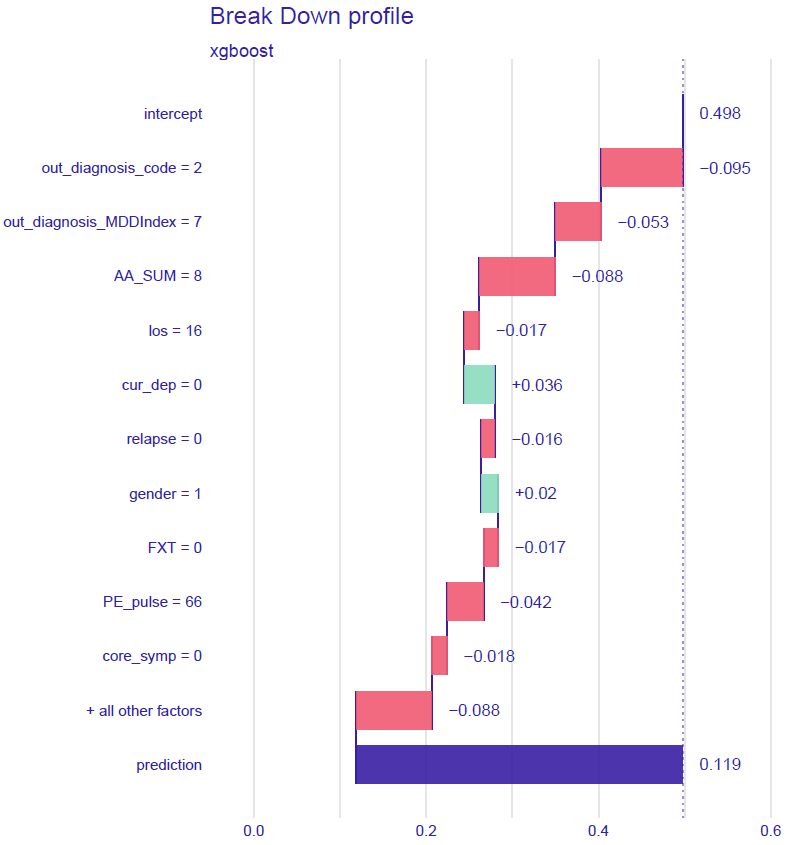


Figure S**14 (A) A readmitted patient in the 30-day cohort (B) A non-readmitted patient in the 30-day cohort**


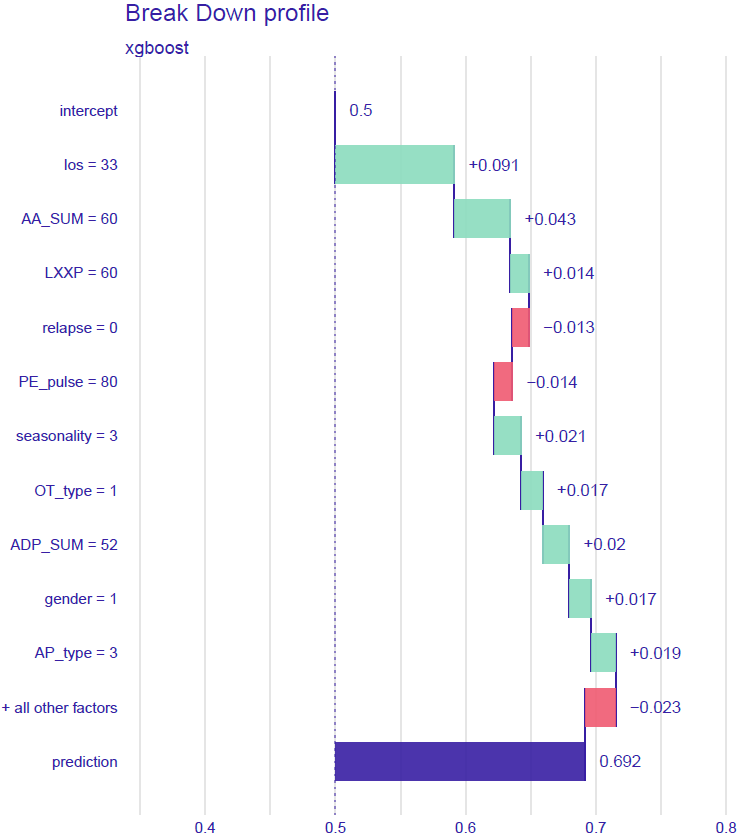

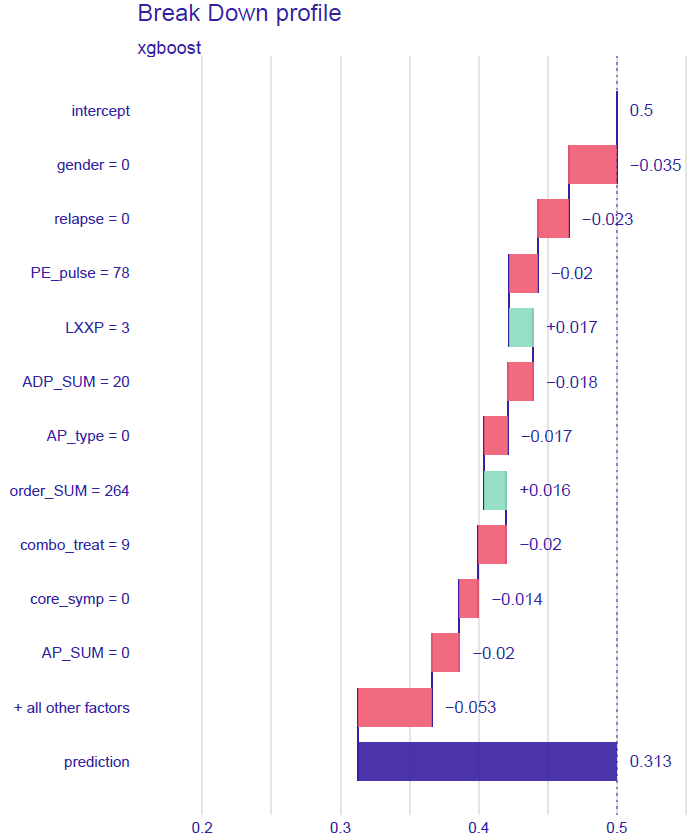


Figure S**14 (C) A readmitted patient in the 60-day cohort (D) A non-readmitted patient in the 60-day cohort**


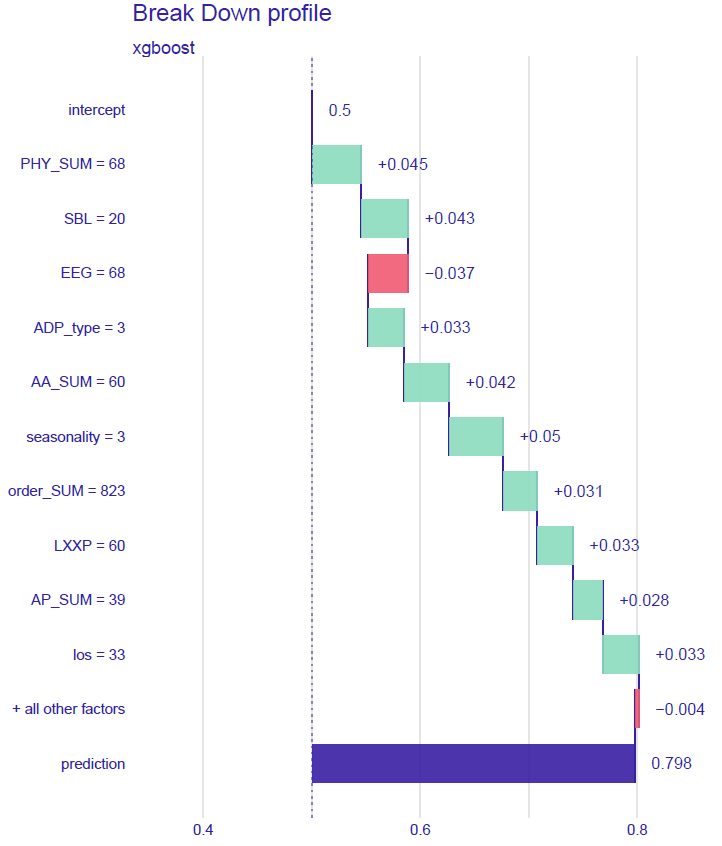

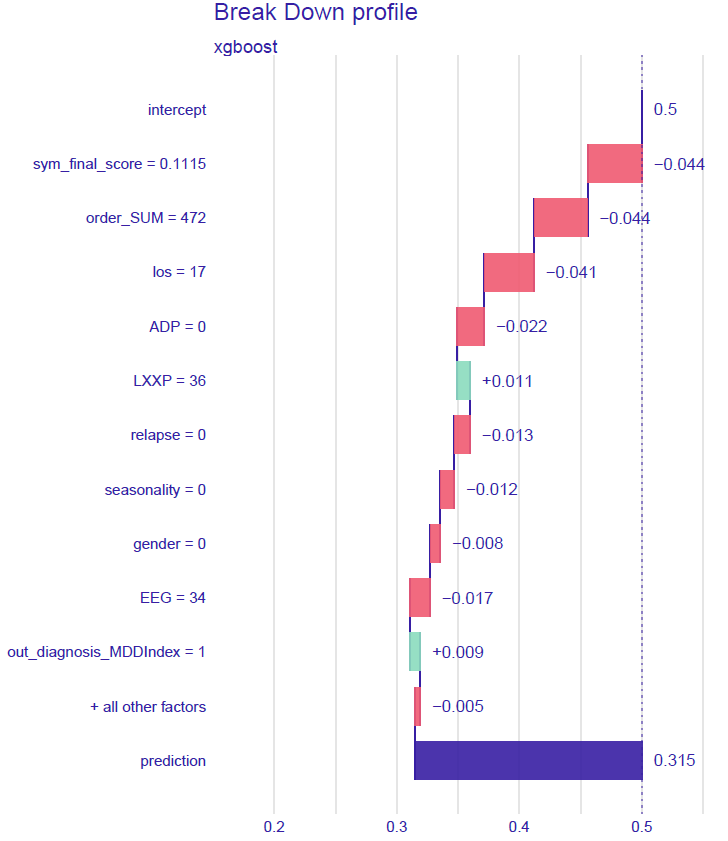


Figure S**14 (E) A readmitted patient in the 90-day cohort**  **(F) A non-readmitted patient in the 90-day cohort**


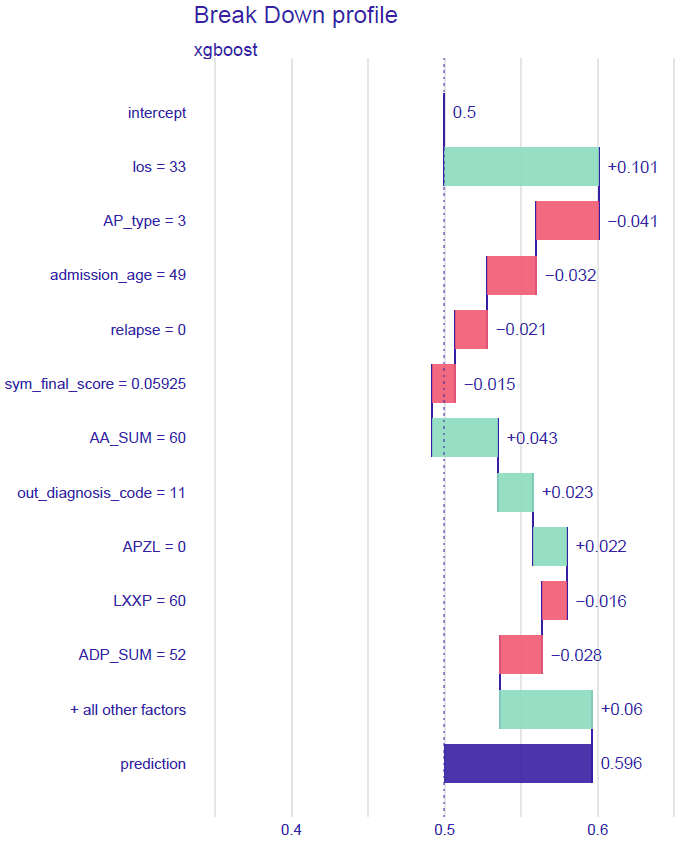

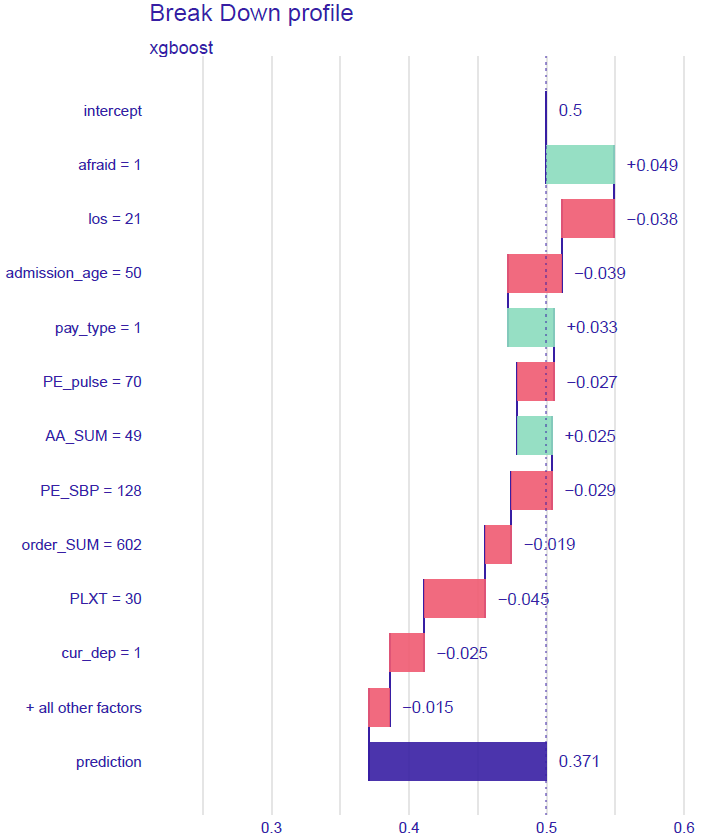


Figure S**14 (G) A readmitted patient in the 180-day cohort**  **(H) A non-readmitted patient in the 180-day cohort**


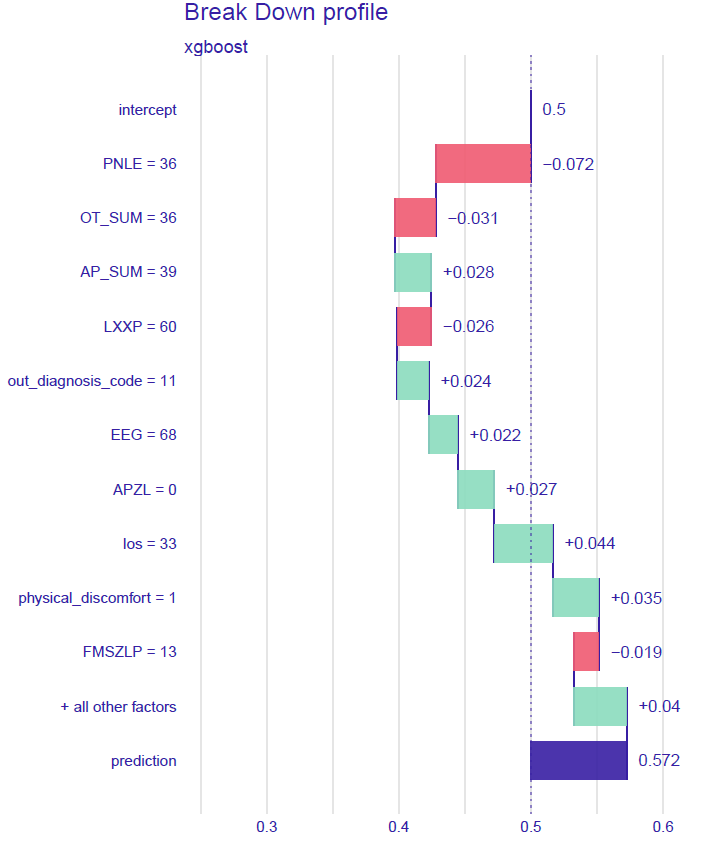

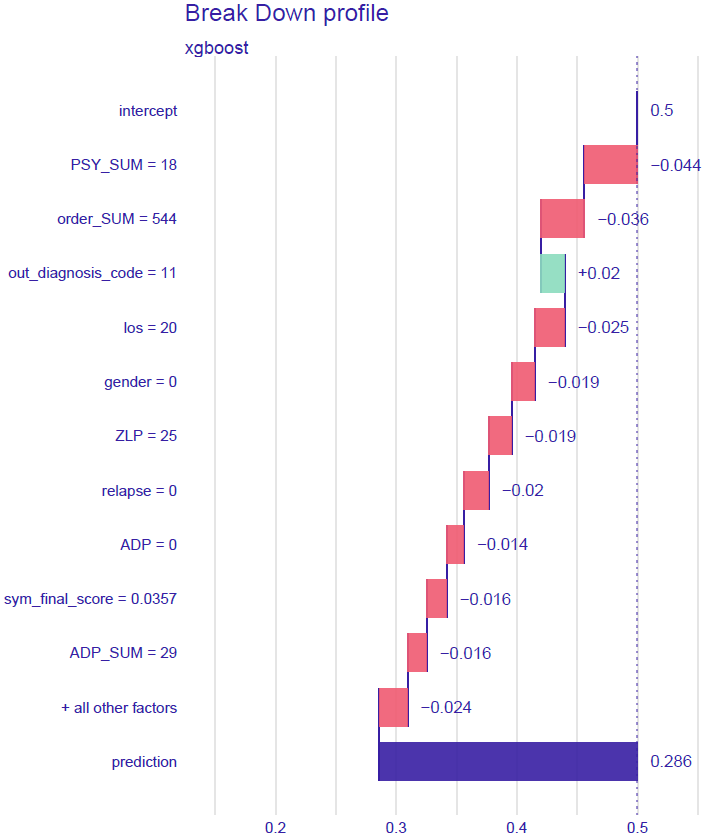


Figure S**14 (I) A readmitted patient in the 365-day cohort**  **(J) A non-readmitted patient in the 365-day cohort**
